# Supplementary material for: A comprehensive computational study of amino acid interactions in membrane proteins
Source: Sci Rep. 2019 Aug 19;9:12043. doi: 10.1038/s41598-019-48541-2 (PMC6700154; doi:10.1038/s41598-019-48541-2)
Supplement: Supplementary file 1 — Supplementary Information [file 41598_2019_48541_MOESM1_ESM.pdf]

Supplementary Material

`

A comprehensive computational study of amino acid interactions in membrane proteins

Mame Ndew Mbaye, Qingzhen Hou, Sankar Basu, Fabian Teheux,
Fabrizio Pucci, Marianne Rooman

- **Figure S1**. Relative frequencies of the 20 amino acids in the datasets $\mathcal{D}^{EM}$, $\mathcal{D}^{TM}$ and $\mathcal{D}^{GL}$, as a function of the solvent accessibility of the residues.
- **Figure S2**. Relative frequencies of negatively and positively charged residues in the datasets $\mathcal{D}^{EM}$ and $\mathcal{D}^{TM}$ as a function of the distance with respect to the water-membrane interfaces.
- **Figure S3.** Statistical "sds" residue-residue potentials as a function of the distance, derived from the datasets $\mathcal{D}^{EM}$, $\mathcal{D}^{TM}$ and $\mathcal{D}^{GL}$. The numerical values are given in the file **TableS3.csv** (compressed in SupplementaryInfo.zip).
- **Figure S4.** Statistical "sds" potentials between amino acid groups as a function of the distance, derived from the datasets $\mathcal{D}^{EM}$, $\mathcal{D}^{TM}$ and $\mathcal{D}^{GL}$. The numerical values are given in the file **TableS4.csv** (compressed in SupplementaryInfo.zip).
- **Figure S5.** Statistical "sds" residue-residue potentials as a function of the distance, derived from the dataset $\mathcal{D}^{TM}$, or separately from α-helical and β-barrel transmembrane regions. The numerical values are given in the file **TableS5.csv** (compressed in SupplementaryInfo.zip).
- **Figure S6.** Statistical "sds" potentials between amino acid groups as a function of the distance, derived from the dataset $\mathcal{D}^{TM}$, or separately from α-helical and β-barrel transmembrane regions. The numerical values are given in file **TableS6.csv** (compressed in SupplementaryInfo.zip).
- **Table S1.** Membrane protein dataset (file **TableS1.xlsx** in SupplementaryInfo.zip).
- **Table S2**. Transmembrane protein segments in the dataset (file **TableS2.xlsx** in SupplementaryInfo.zip).

Figure S1. Relative frequencies of the 20 amino acids in the datasets $\mathcal{D}^{EM}$, $\mathcal{D}^{GL}$, $\mathcal{D}^{TM}$, as a function of the solvent accessibility of the residues: 0-20% (blue), 20-50% (orange), and 50-100% (grey).

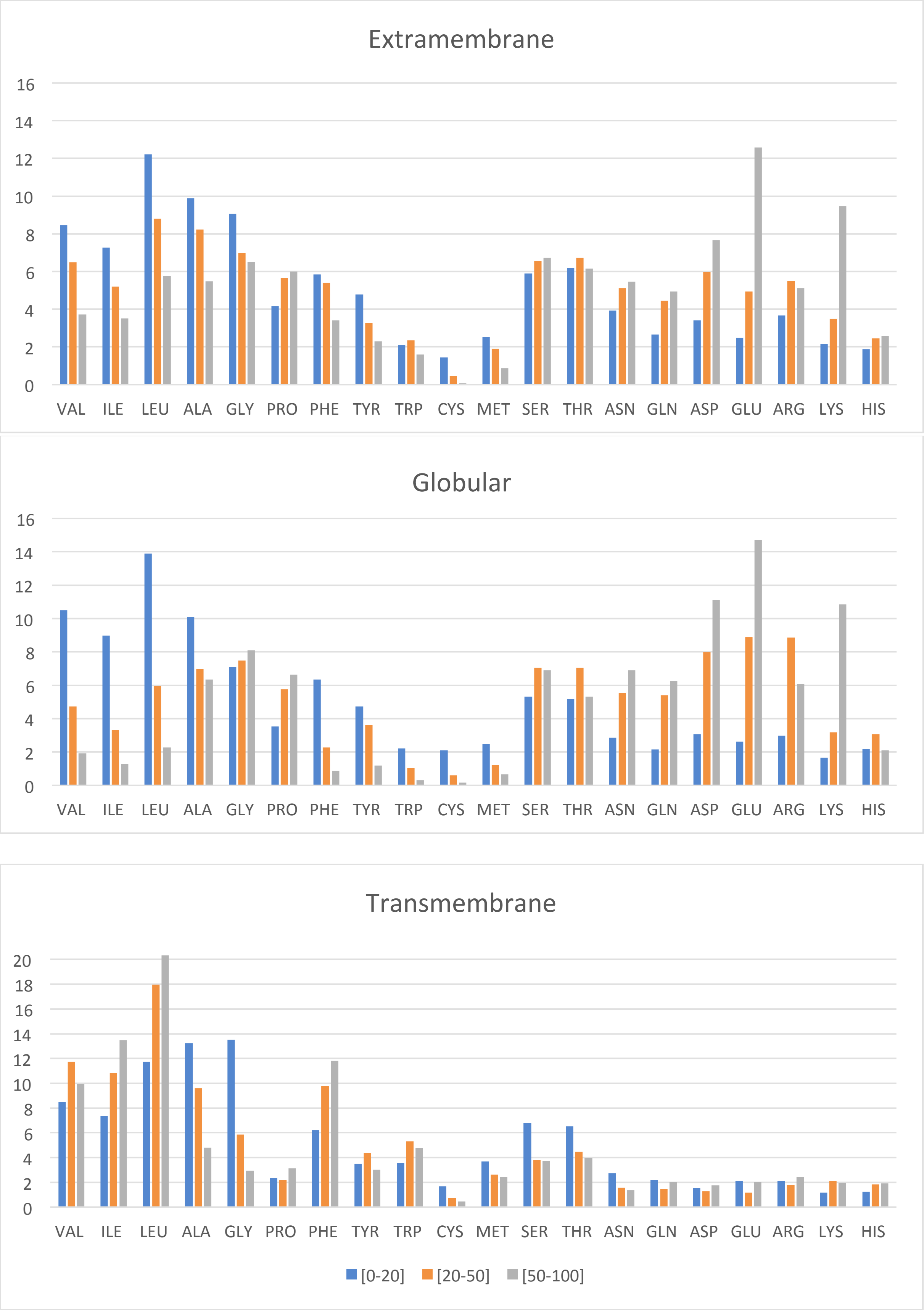

Figure S2. Relative frequencies of negatively and positively charged residues in the datasets $\mathcal{D}^{EM}$ and $\mathcal{D}^{TM}$ as a function of the distance with respect to the water-membrane interfaces. Darker blue indicates higher frequencies and lighter blue lower frequencies. The straight lines represent the water-membrane interfaces. The extracellular side is directed upward and the intracellular side downward. In the second row, the frequencies are computed from membrane proteins whose transmembrane domain is α-helical, and in the third row, they are computed from proteins whose transmembrane domain has a β-barrel conformation.

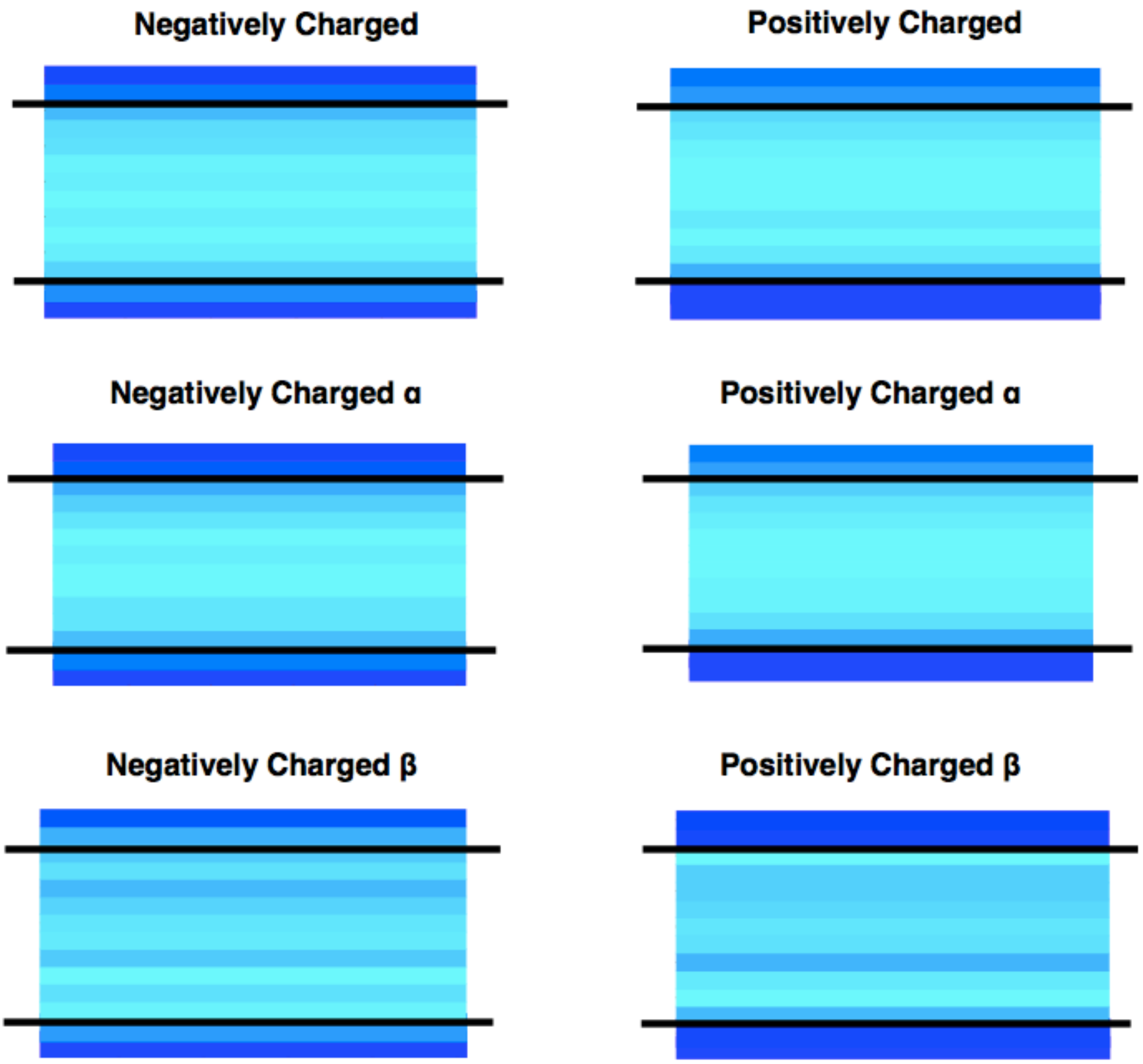

Figure S3. Statistical "sds" residue-residue potentials as a function of the distance (in Å), derived from the datasets $\mathcal{D}^{EM}$ (blue lines), $\mathcal{D}^{TM}$ (red lines), and $\mathcal{D}^{GL}$ (black lines). The numerical values are given in the Supplementary file Figure_S3.csv.

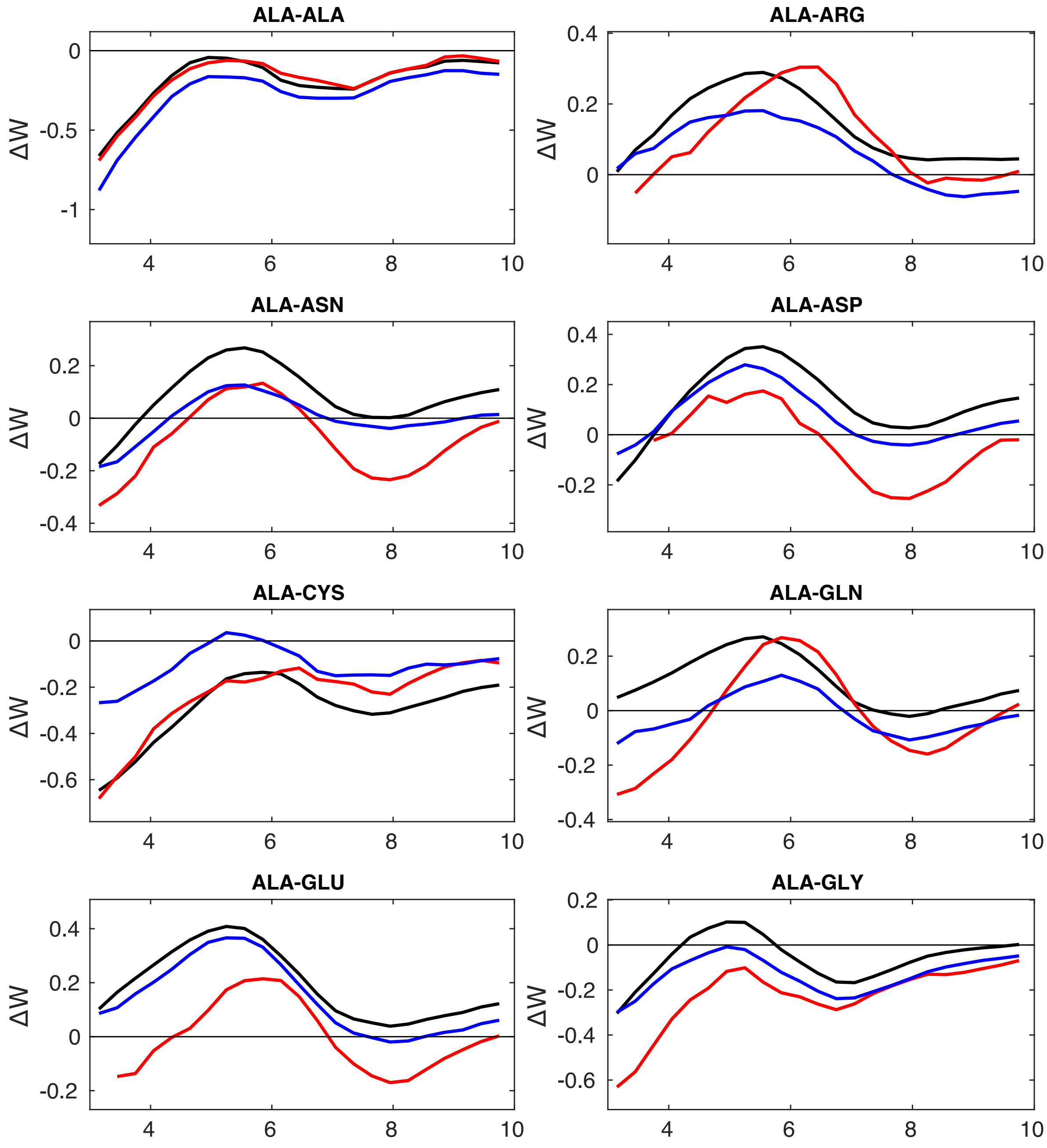

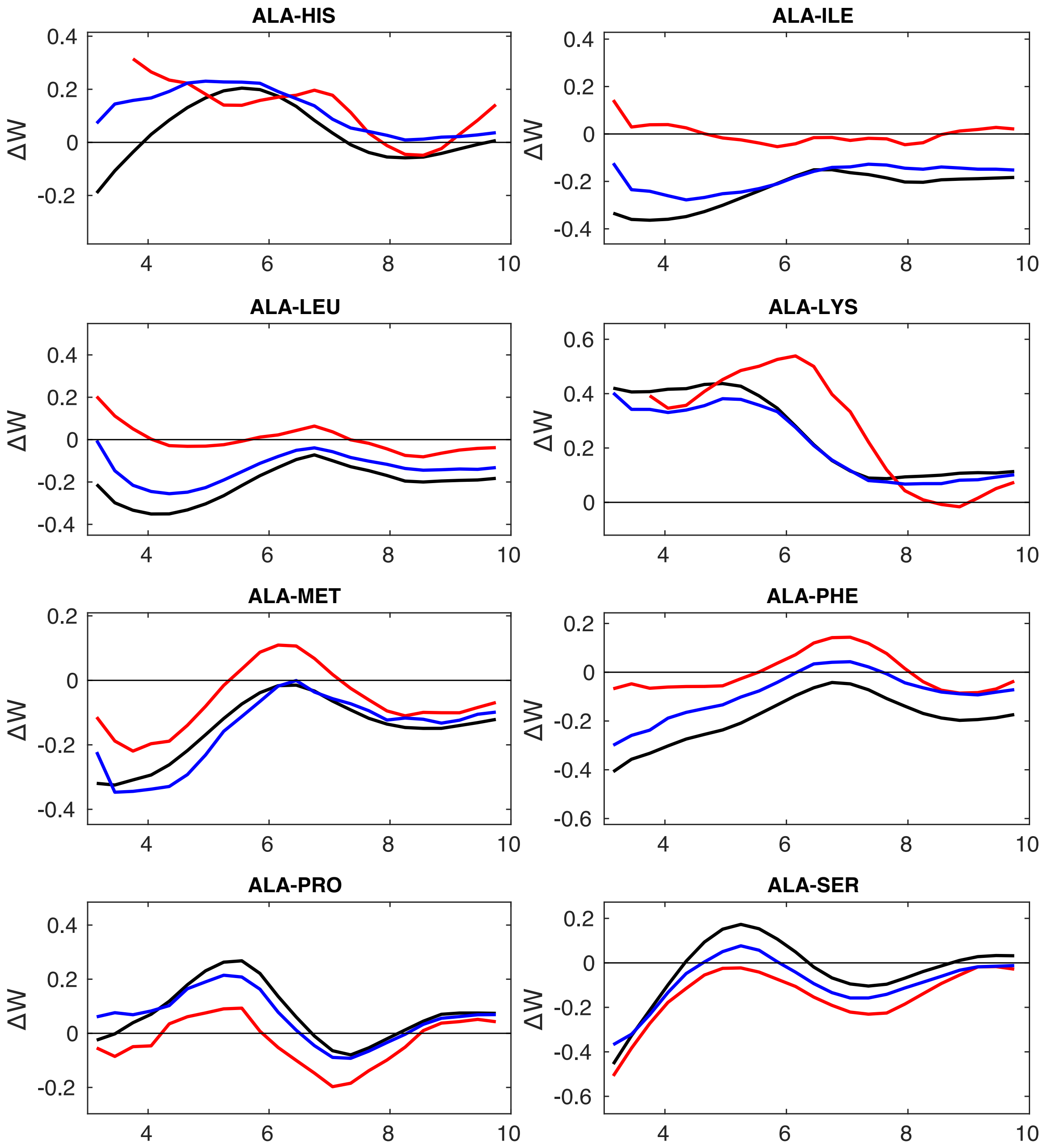
ALA-HIS
ALA-ILE
ALA-LEU
ALA-LYS
ALA-MET
ALA-PHE
ALA-PRO
ALA-SER
ΔW

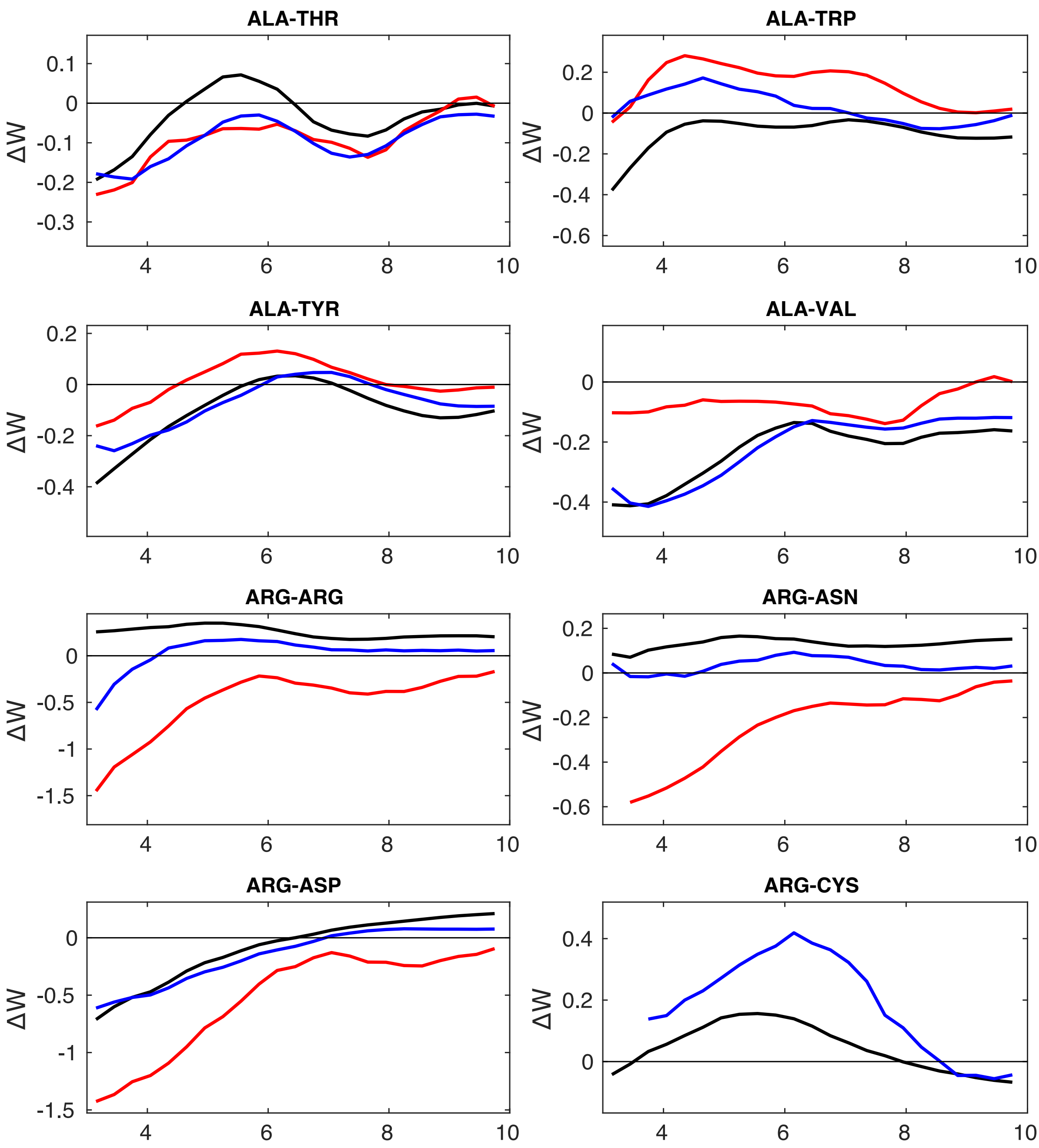
ALA-THR
ALA-TRP
ALA-TYR
ALA-VAL
ARG-ARG
ARG-ASN
ARG-ASP
ARG-CYS
ΔW
0.1
0
-0.1
-0.2
-0.3
0.2
-0.4
-0.6
-0.5
-1
-1.5
4
6
8
10

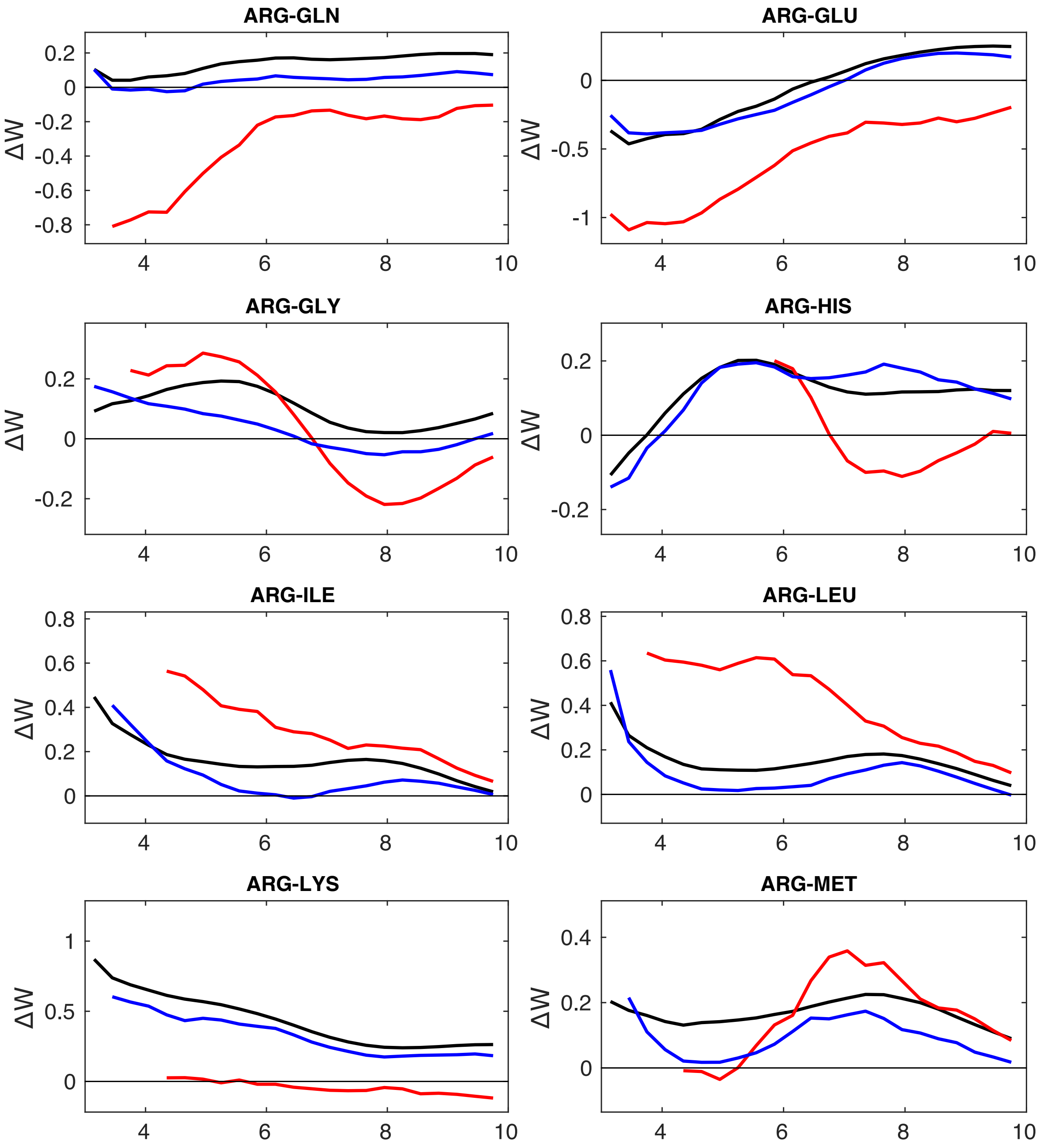
ARG-GLN
ARG-GLU
ARG-GLY
ARG-HIS
ARG-ILE
ARG-LEU
ARG-LYS
ARG-MET
ΔW
4
6
8
10

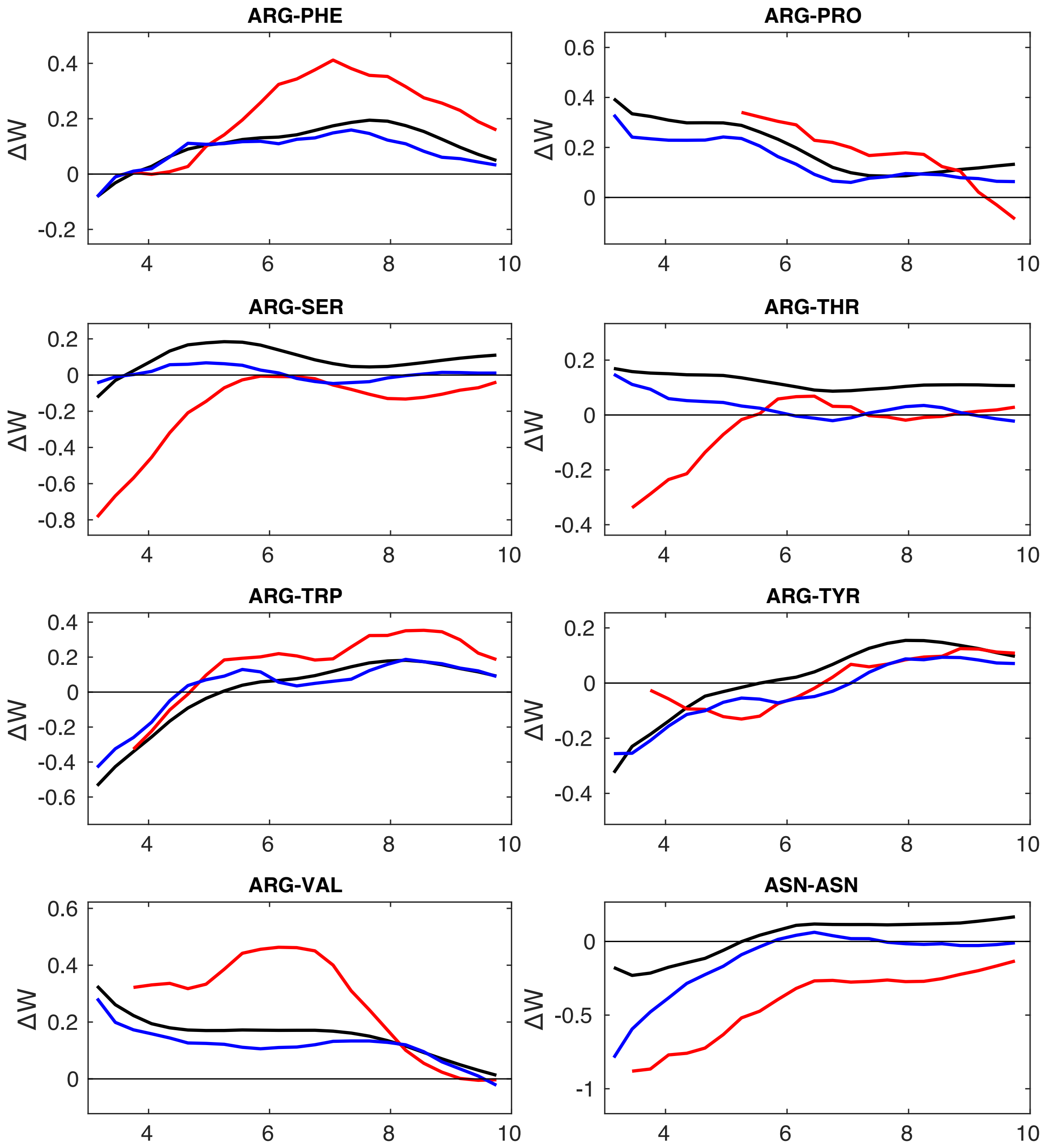
ARG-PHE
ARG-PRO
ARG-SER
ARG-THR
ARG-TRP
ARG-TYR
ARG-VAL
ASN-ASN
ΔW
4
6
8
10

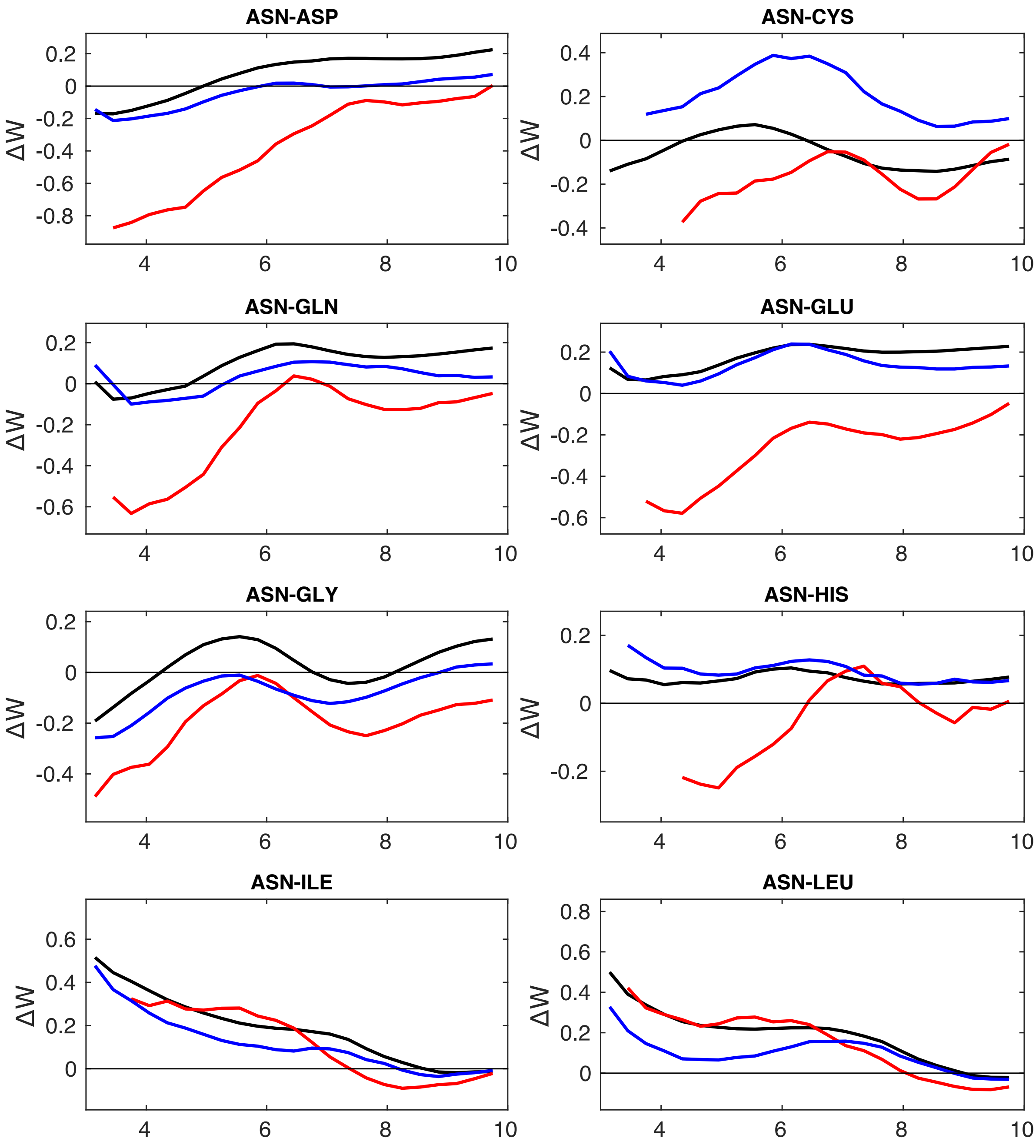
ASN-ASP
ASN-CYS
ASN-GLN
ASN-GLU
ASN-GLY
ASN-HIS
ASN-ILE
ASN-LEU
ΔW

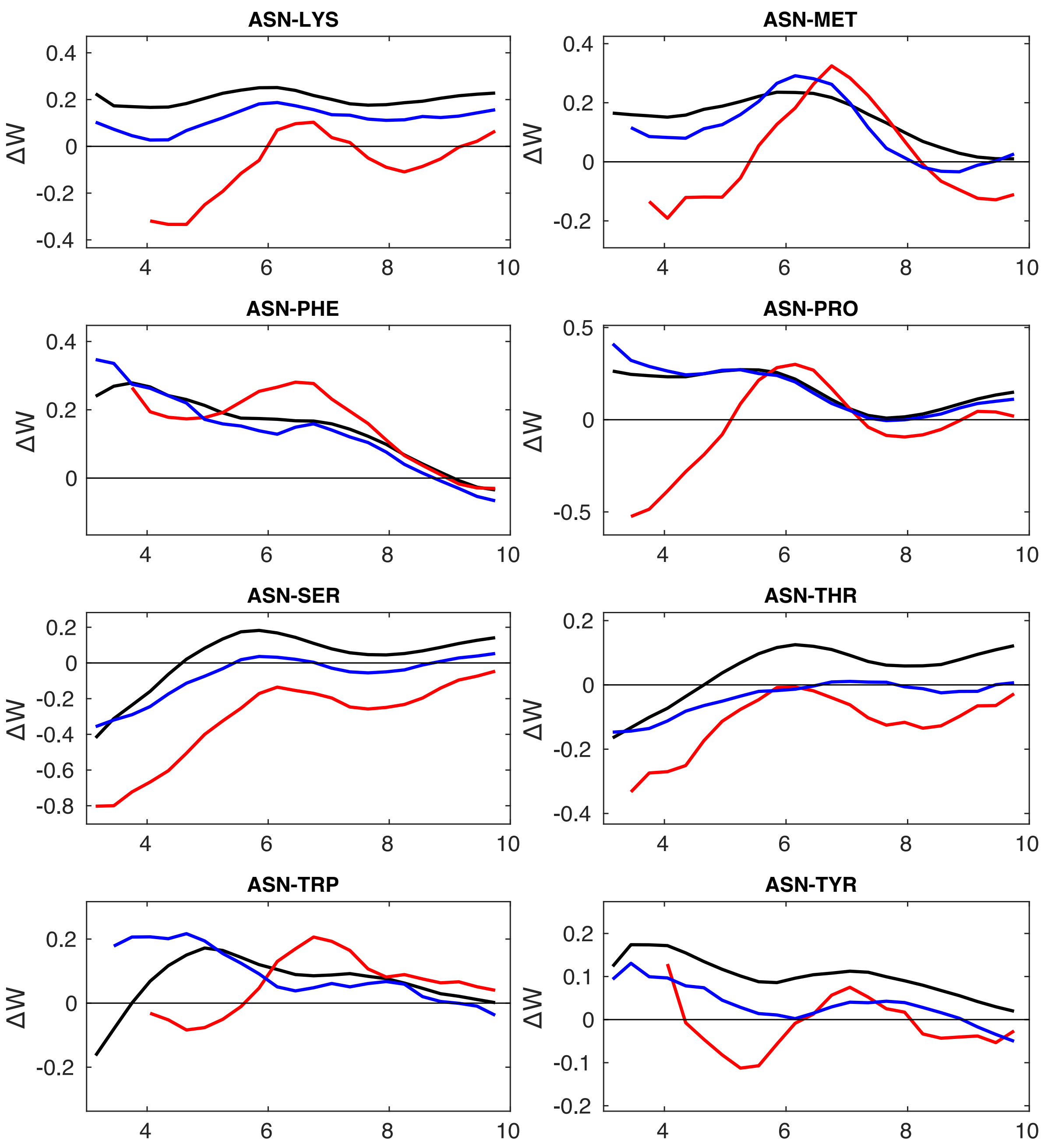
ASN-LYS
ASN-MET
ASN-PHE
ASN-PRO
ASN-SER
ASN-THR
ASN-TRP
ASN-TYR
ΔW
4
6
8
10

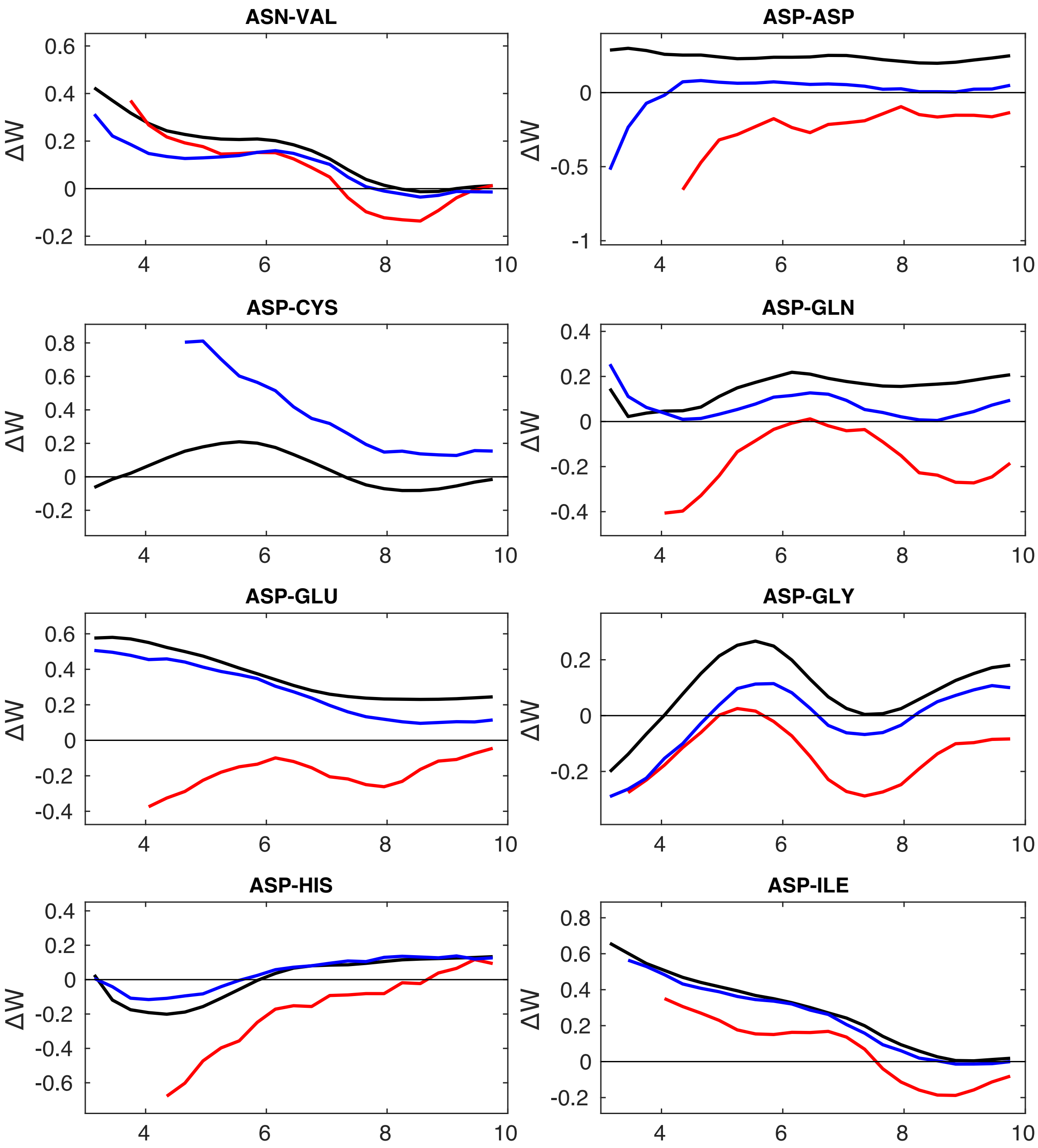
ASN-VAL
ASP-ASP
ASP-CYS
ASP-GLN
ASP-GLU
ASP-GLY
ASP-HIS
ASP-ILE
ΔW

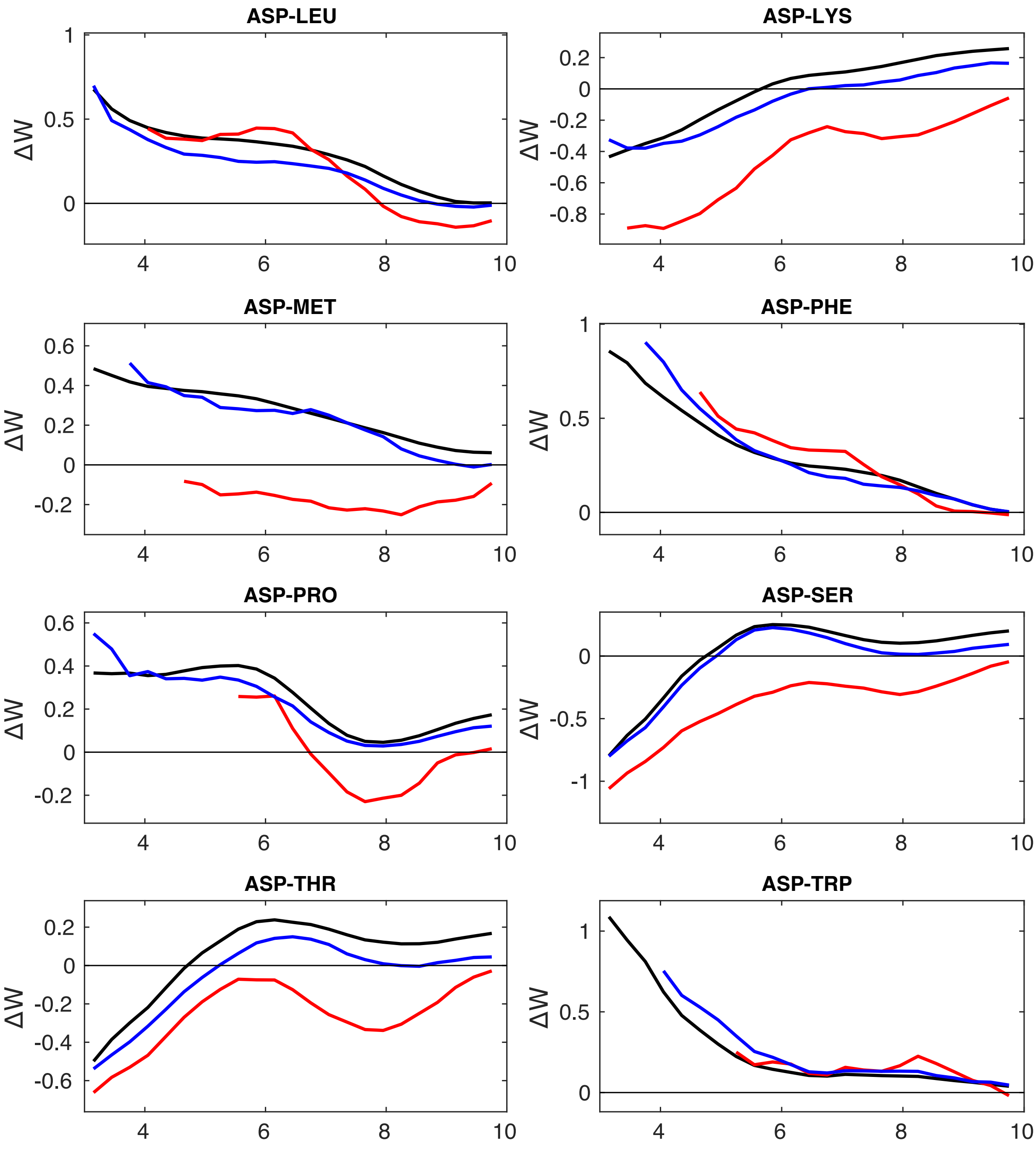
ASP-LEU
ASP-LYS
ASP-MET
ASP-PHE
ASP-PRO
ASP-SER
ASP-THR
ASP-TRP
ΔW

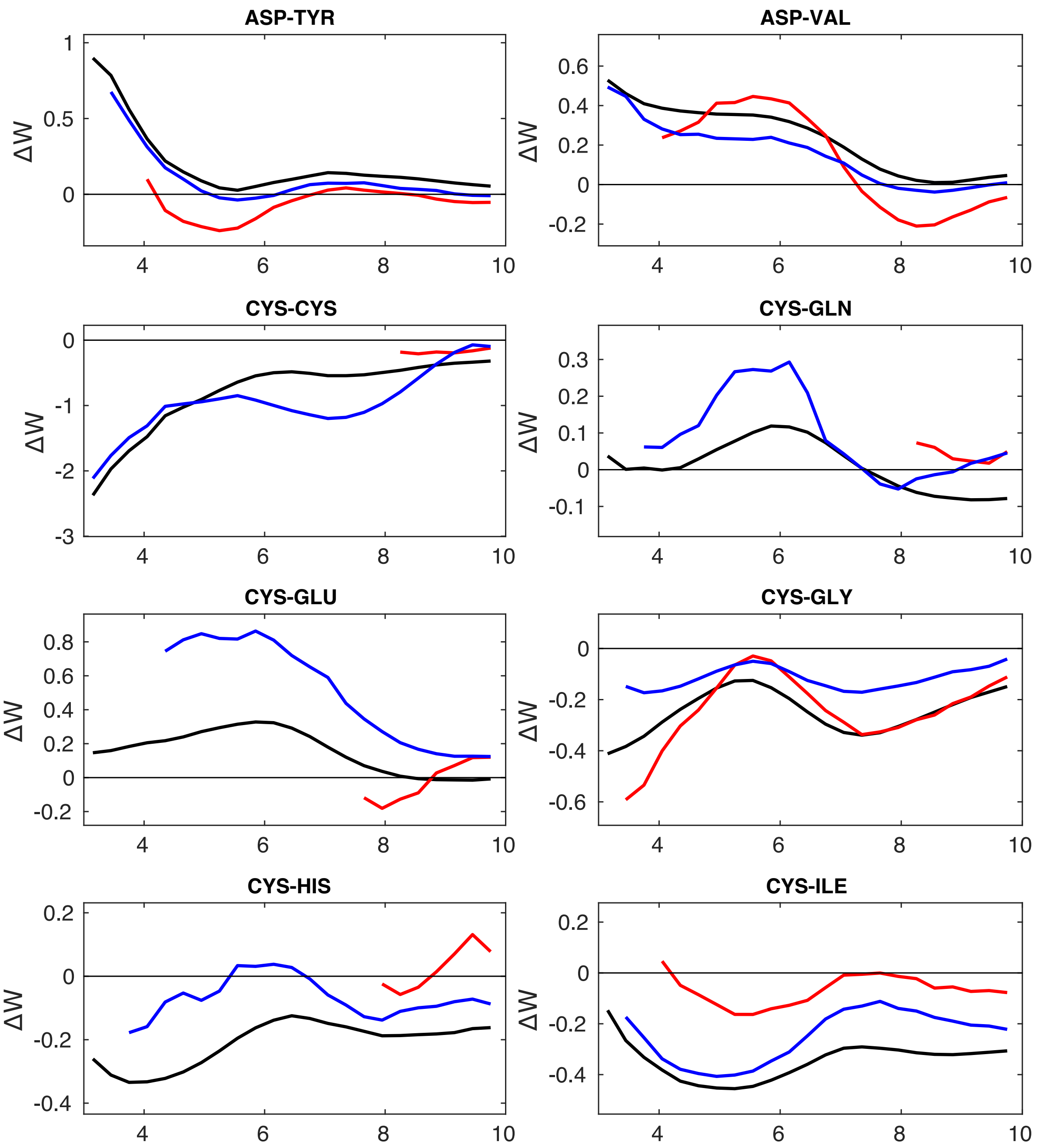

ASP-TYR
ASP-VAL
CYS-CYS
CYS-GLN
CYS-GLU
CYS-GLY
CYS-HIS
CYS-ILE
ΔW

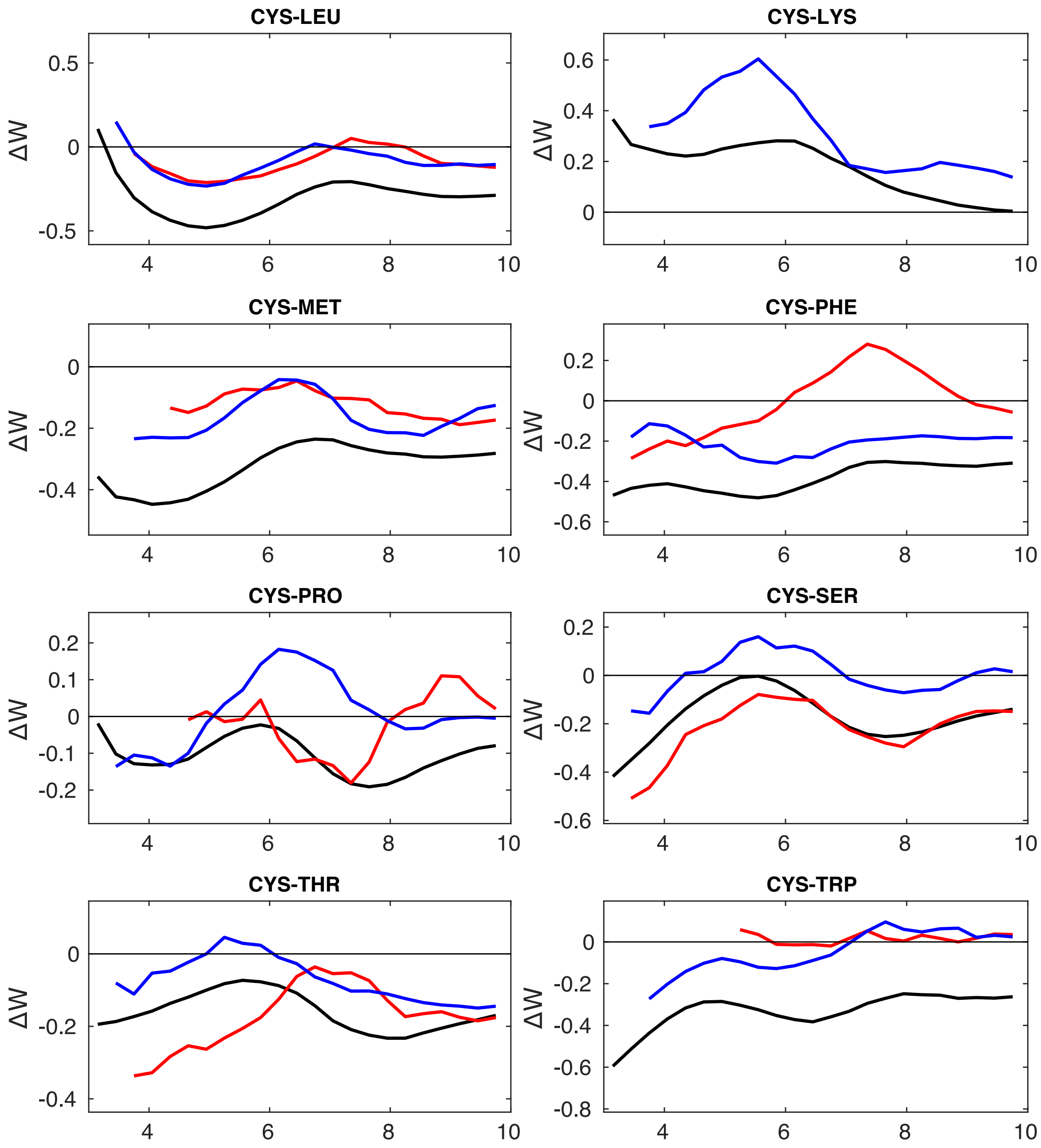
CYS-LEU
CYS-LYS
CYS-MET
CYS-PHE
CYS-PRO
CYS-SER
CYS-THR
CYS-TRP
ΔW

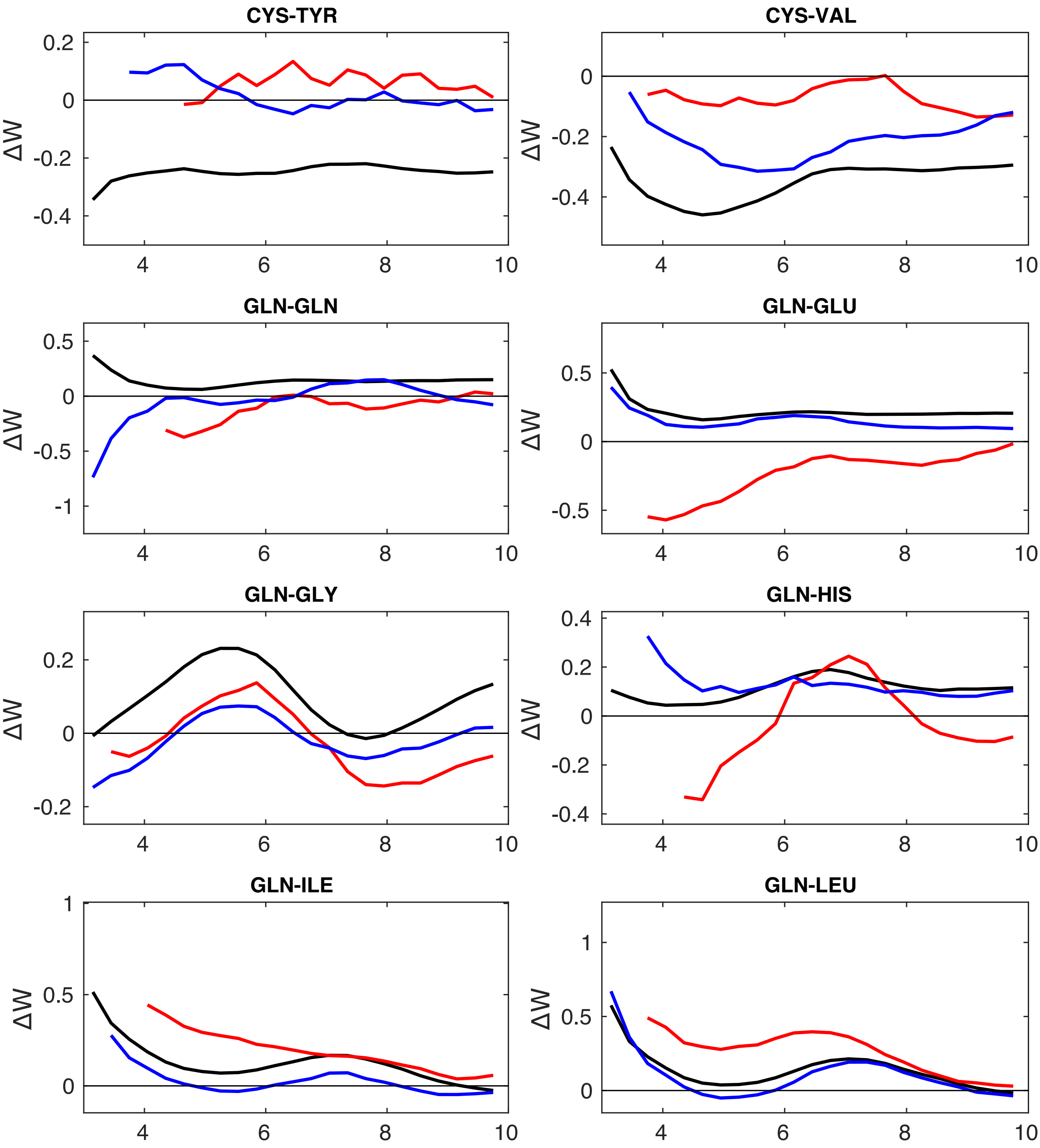
CYS-TYR
CYS-VAL
GLN-GLN
GLN-GLU
GLN-GLY
GLN-HIS
GLN-ILE
GLN-LEU
ΔW
4
6
8
10

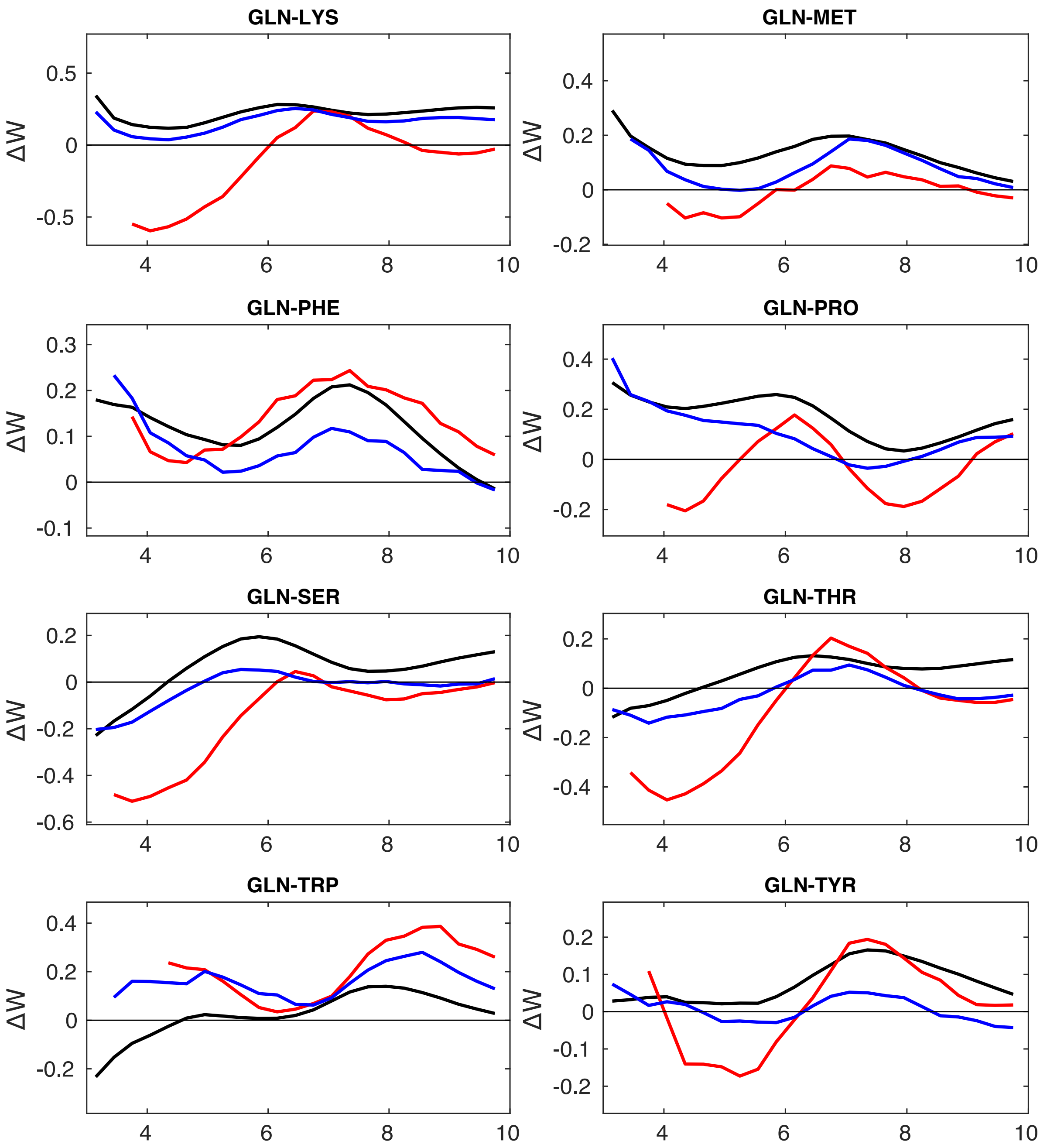
GLN-LYS
GLN-MET
GLN-PHE
GLN-PRO
GLN-SER
GLN-THR
GLN-TRP
GLN-TYR
ΔW

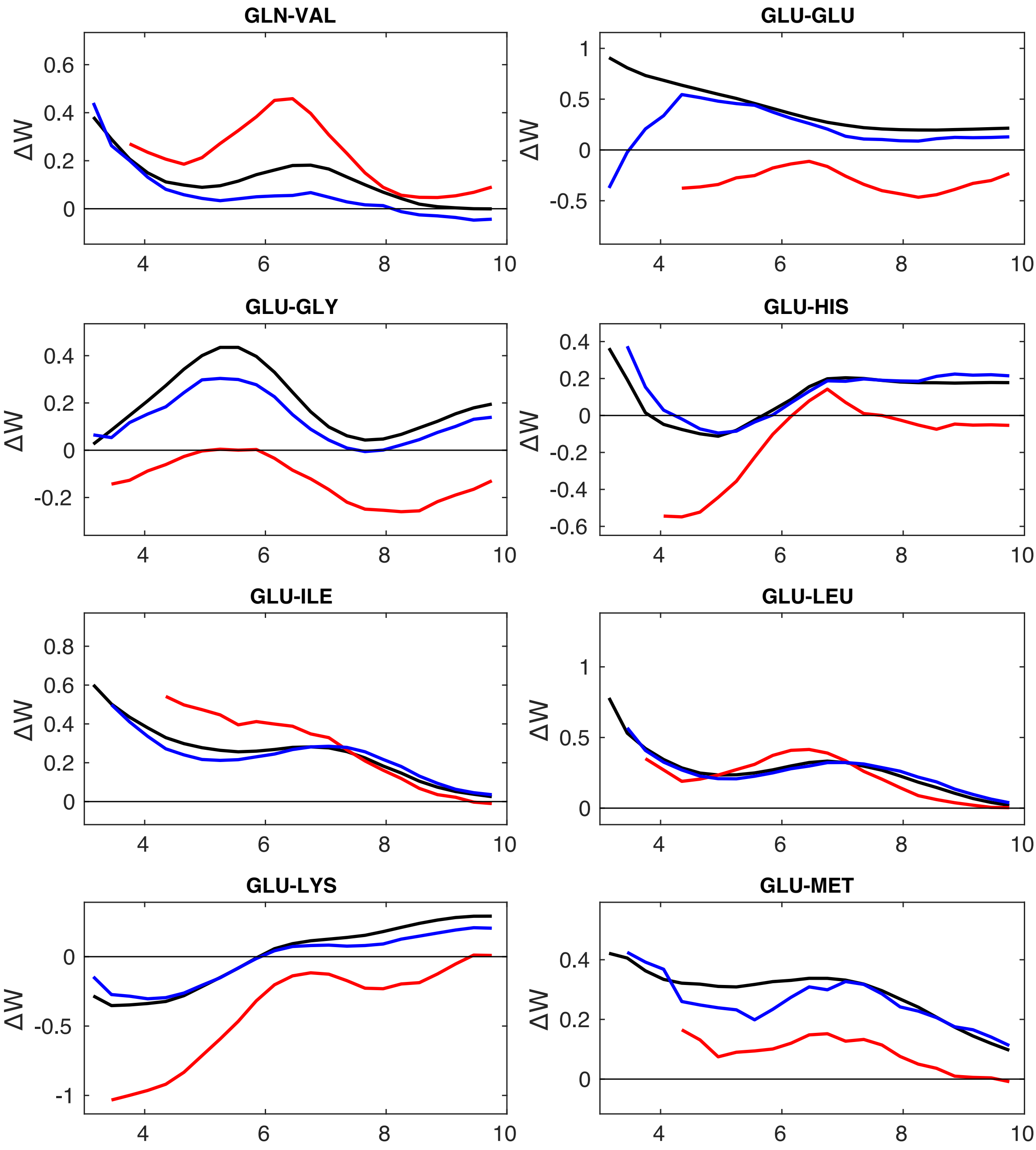

GLN-VAL
GLU-GLU
GLU-GLY
GLU-HIS
GLU-ILE
GLU-LEU
GLU-LYS
GLU-MET
ΔW

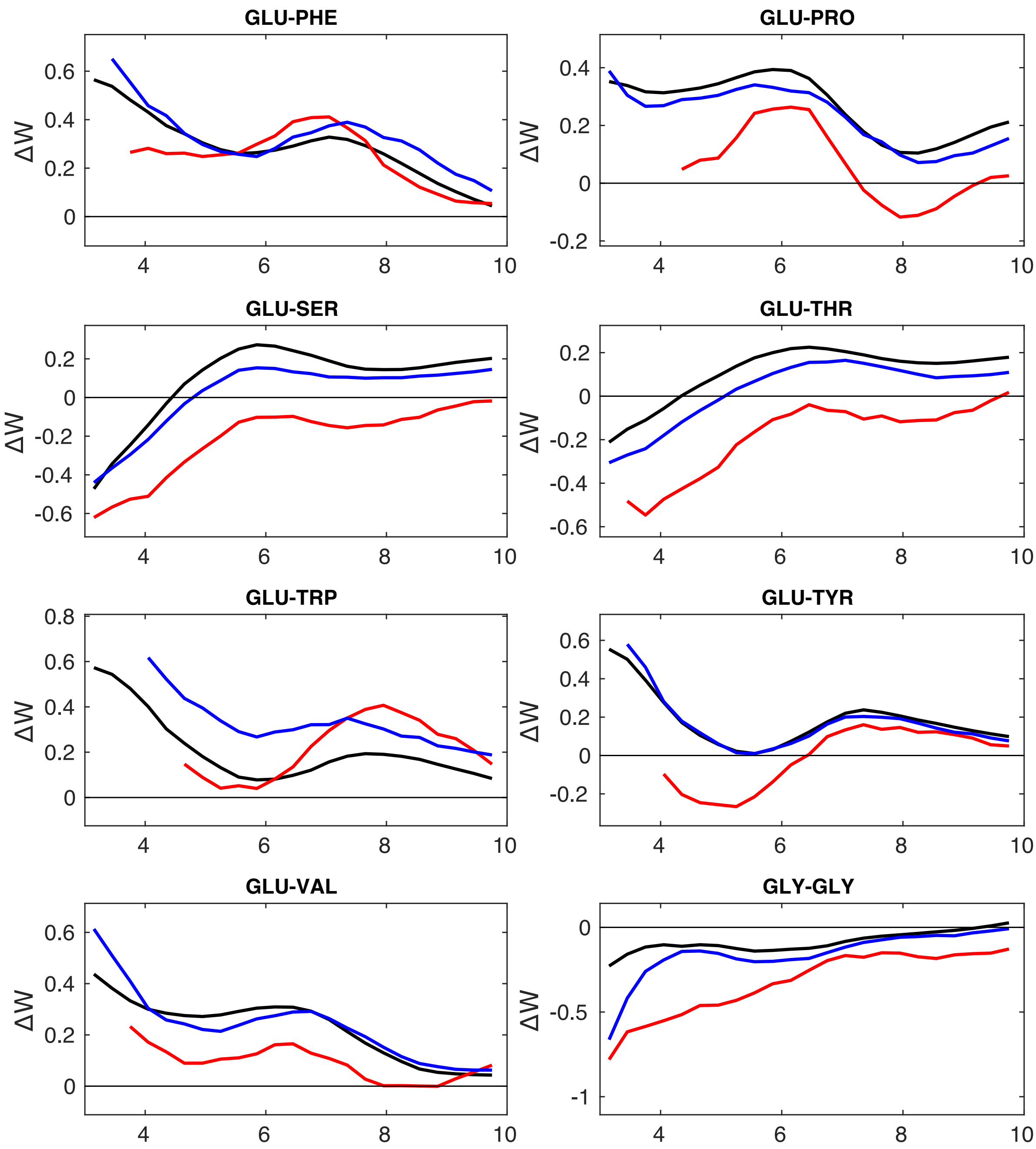
GLU-PHE
GLU-PRO
GLU-SER
GLU-THR
GLU-TRP
GLU-TYR
GLU-VAL
GLY-GLY
ΔW

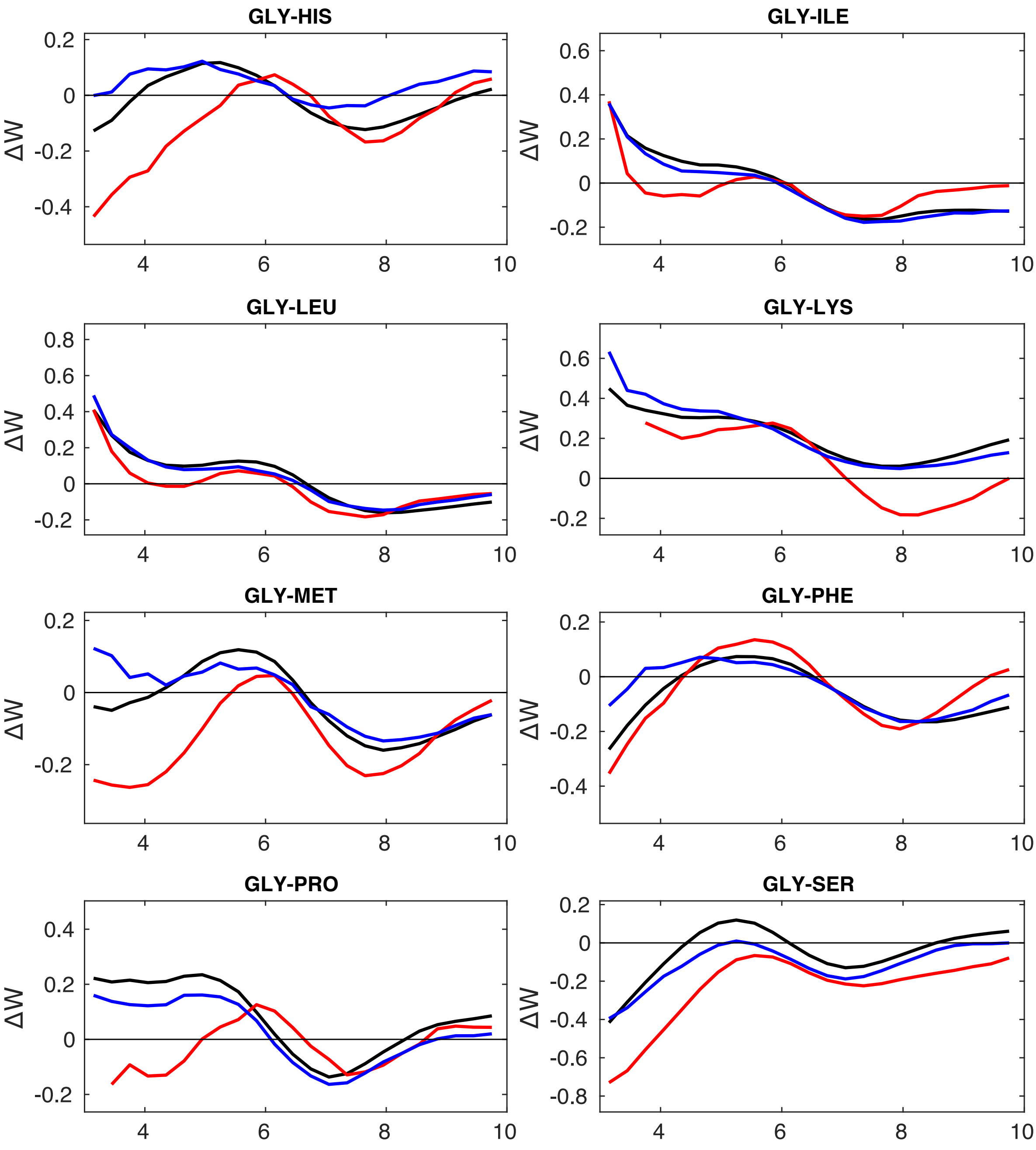
GLY-HIS
GLY-ILE
GLY-LEU
GLY-LYS
GLY-MET
GLY-PHE
GLY-PRO
GLY-SER
ΔW
4
6
8
10

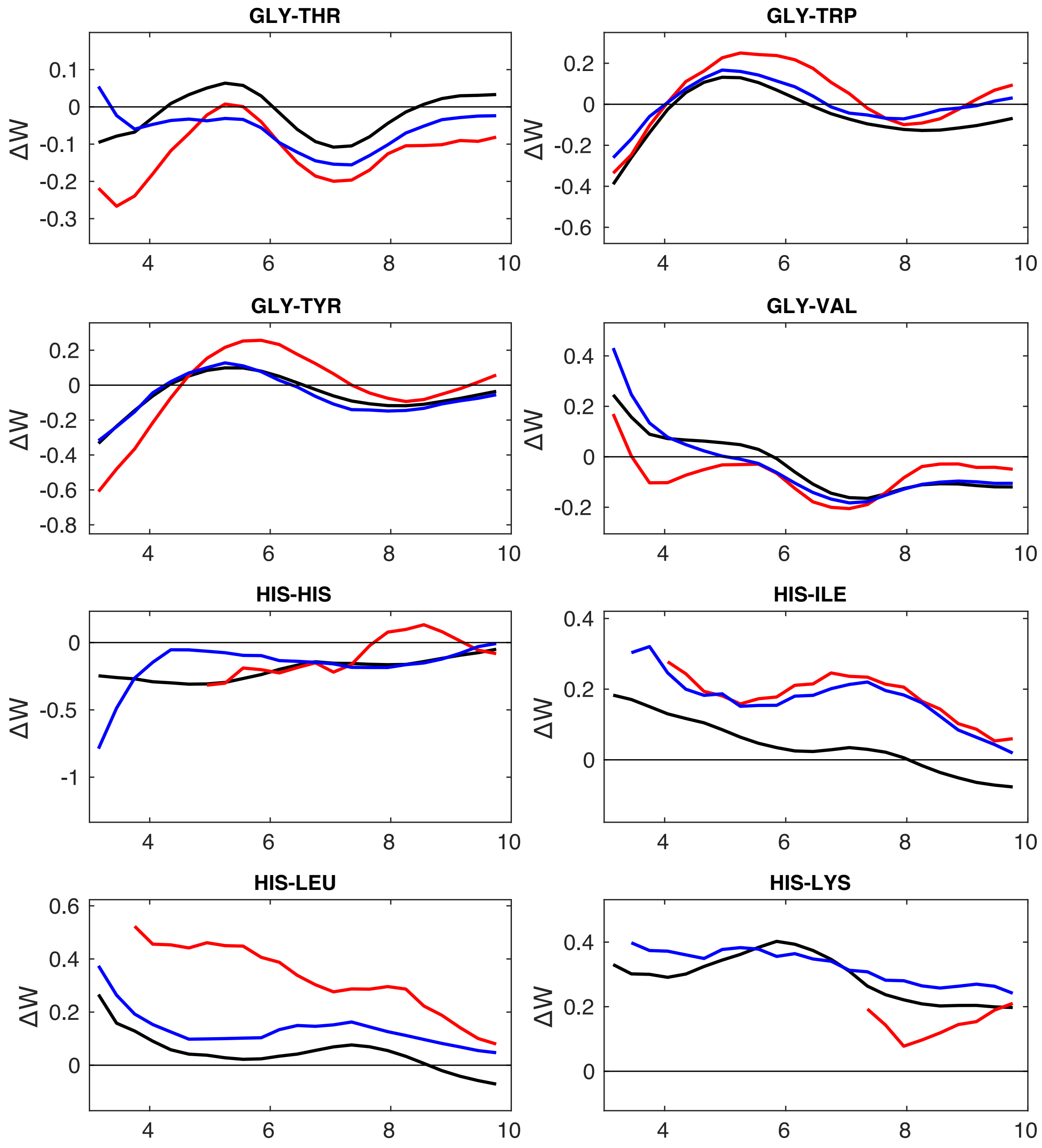
GLY-THR
GLY-TRP
GLY-TYR
GLY-VAL
HIS-HIS
HIS-ILE
HIS-LEU
HIS-LYS
ΔW

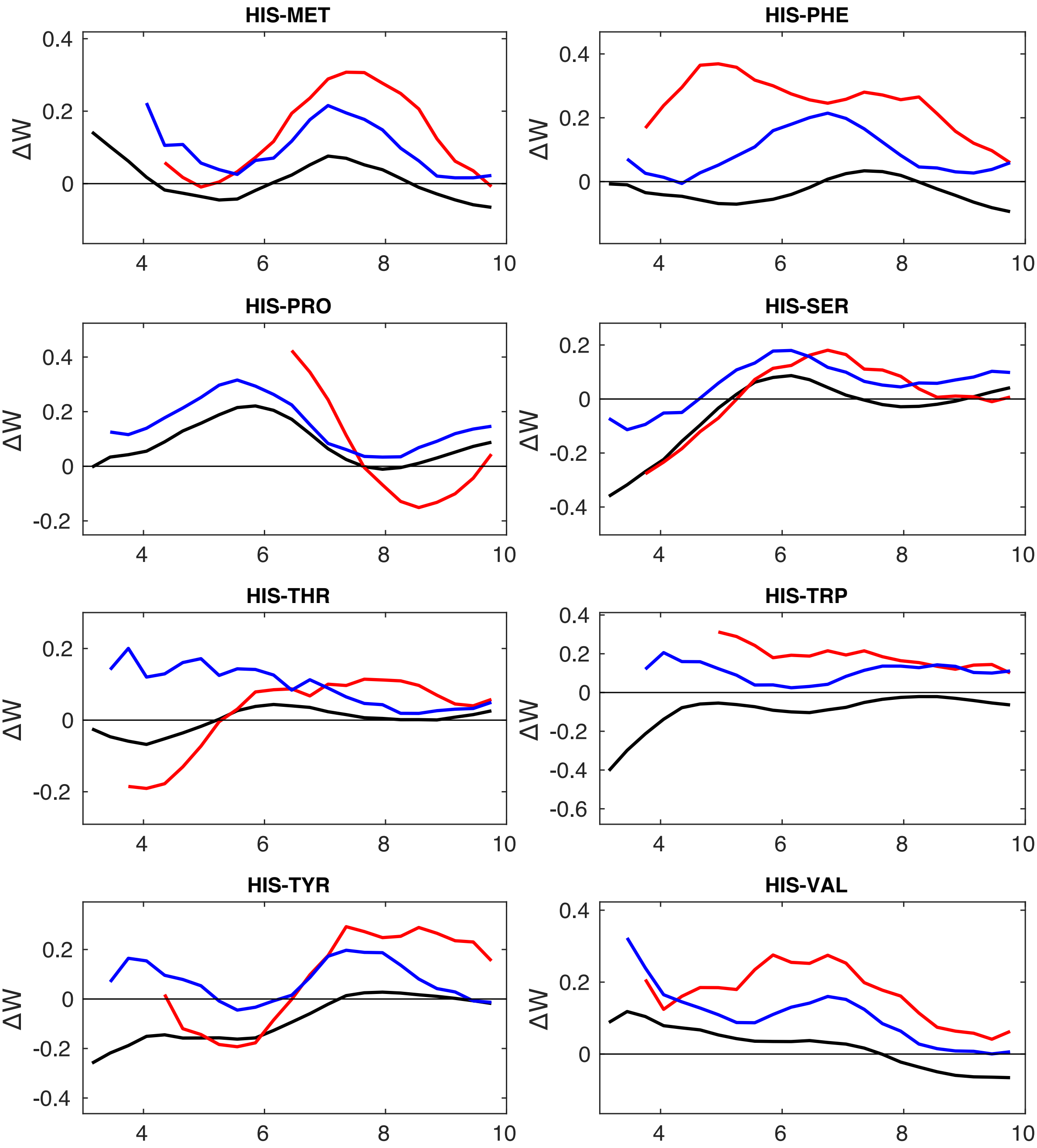
HIS-MET
HIS-PHE
HIS-PRO
HIS-SER
HIS-THR
HIS-TRP
HIS-TYR
HIS-VAL
ΔW

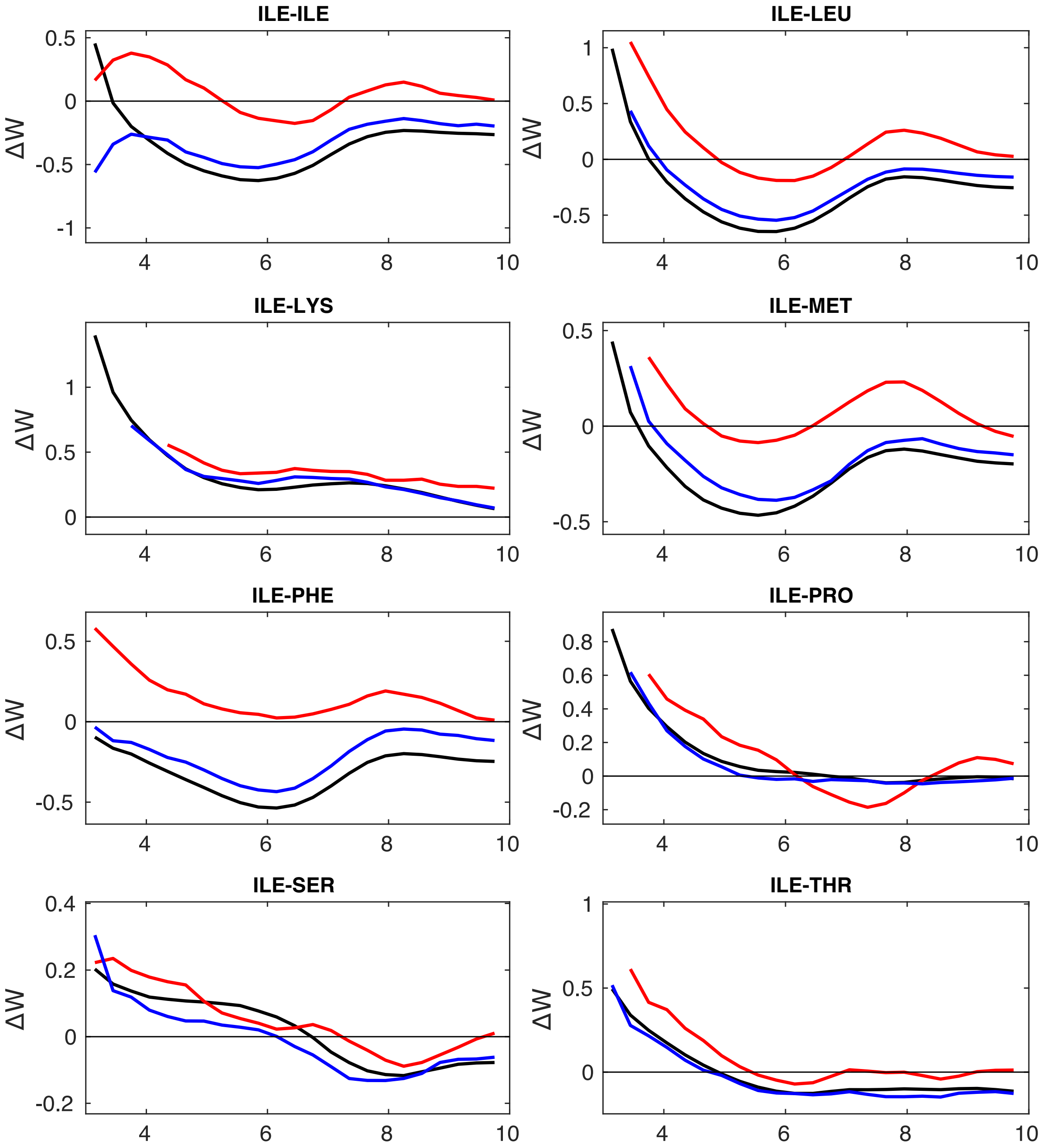
ILE-ILE
ILE-LEU
ILE-LYS
ILE-MET
ILE-PHE
ILE-PRO
ILE-SER
ILE-THR
ΔW

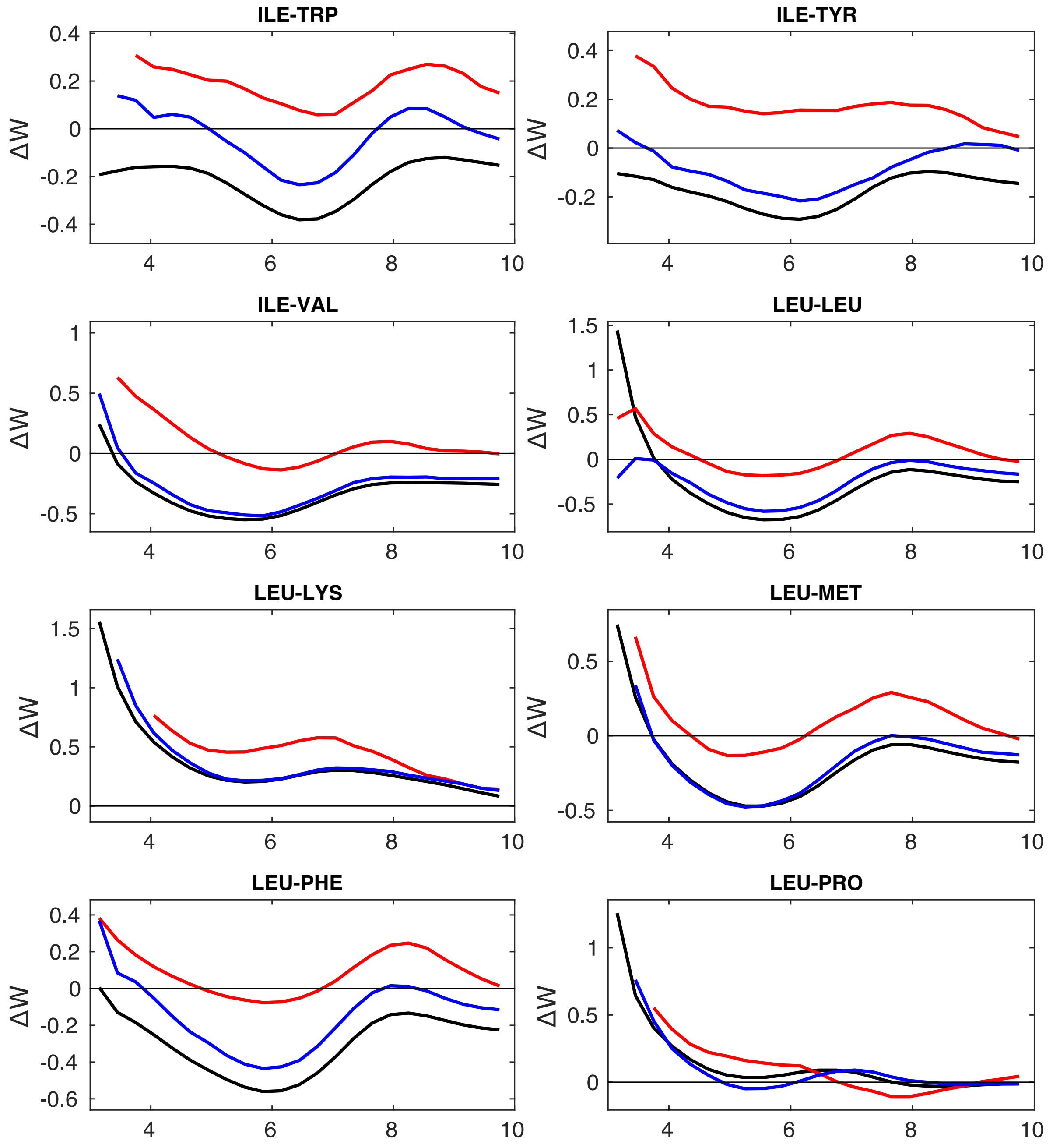
ILE-TRP
ILE-TYR
ILE-VAL
LEU-LEU
LEU-LYS
LEU-MET
LEU-PHE
LEU-PRO
ΔW

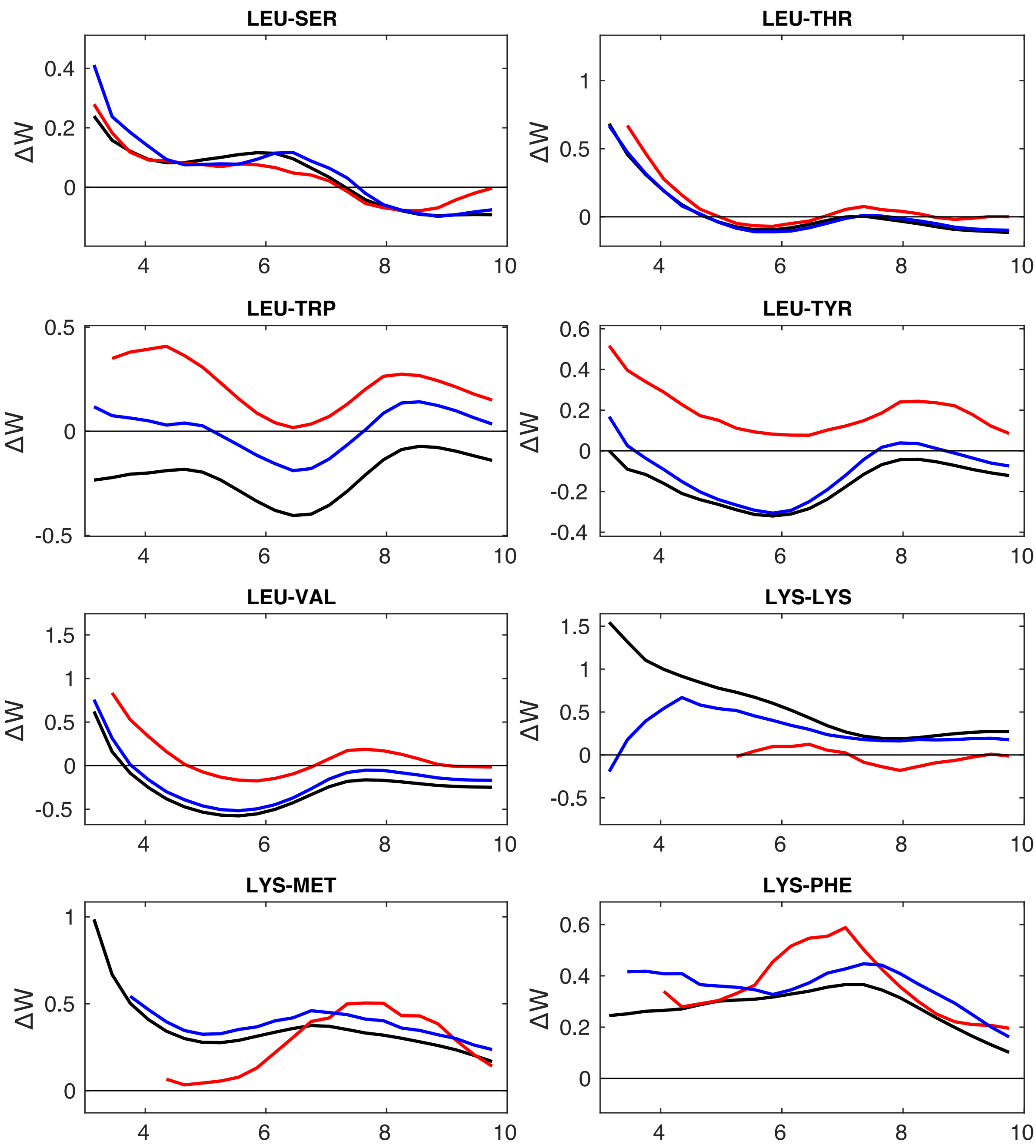
LEU-SER
LEU-THR
LEU-TRP
LEU-TYR
LEU-VAL
LYS-LYS
LYS-MET
LYS-PHE
ΔW
4
6
8
10

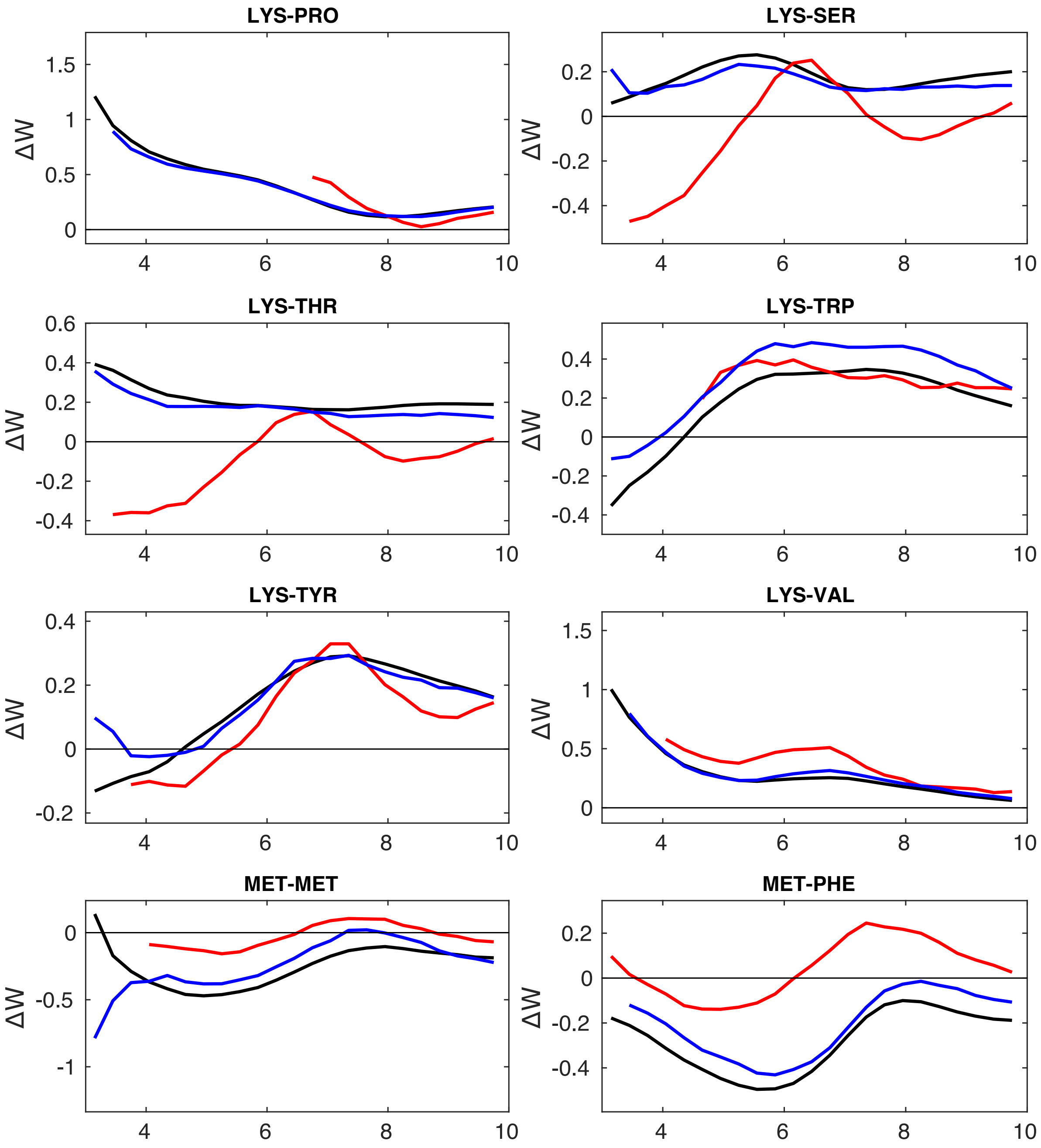
LYS-PRO
LYS-SER
LYS-THR
LYS-TRP
LYS-TYR
LYS-VAL
MET-MET
MET-PHE
ΔW
4
6
8
10

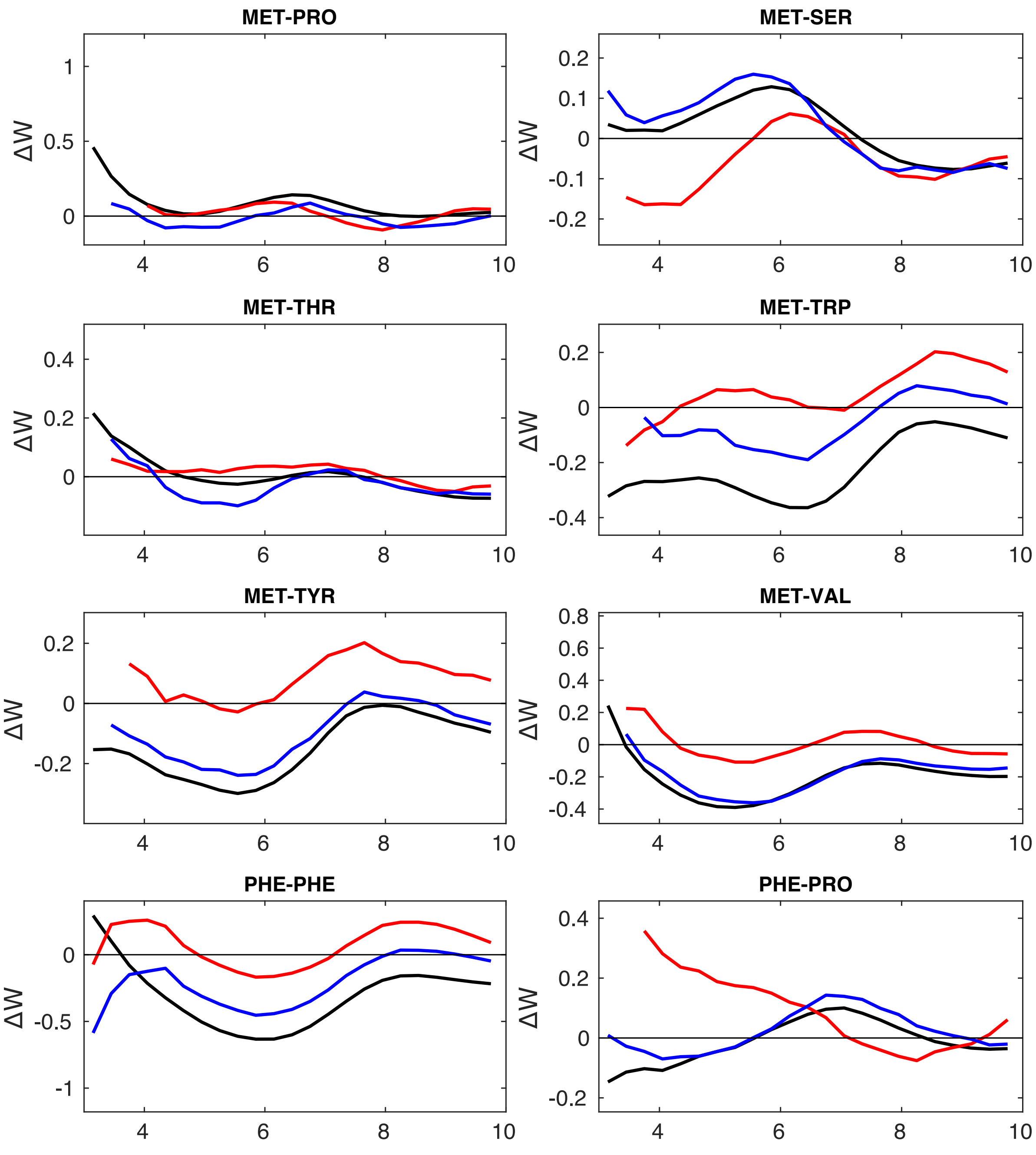
MET-PRO
MET-SER
MET-THR
MET-TRP
MET-TYR
MET-VAL
PHE-PHE
PHE-PRO
ΔW

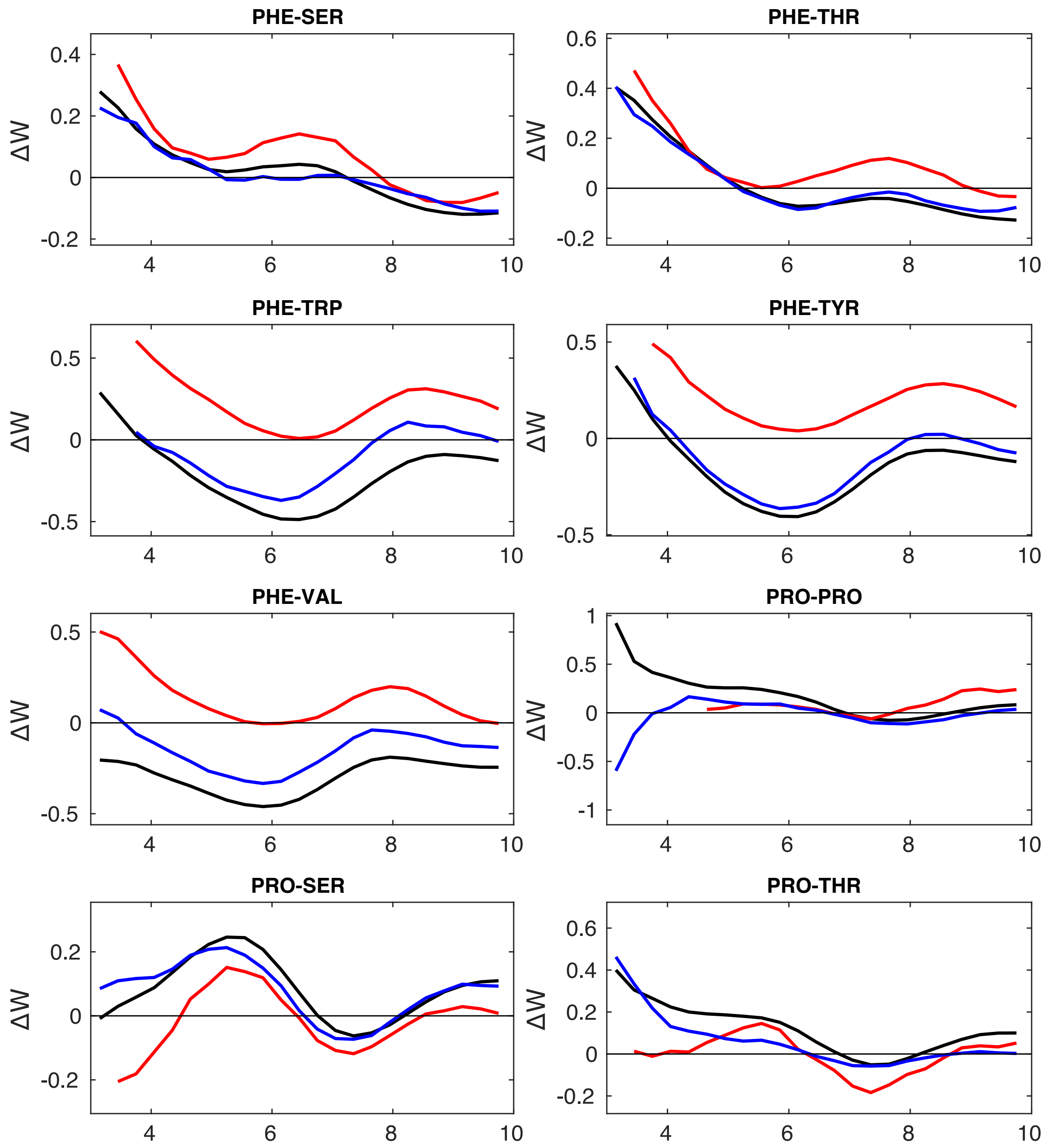

PHE-SER
PHE-THR
PHE-TRP
PHE-TYR
PHE-VAL
PRO-PRO
PRO-SER
PRO-THR
ΔW

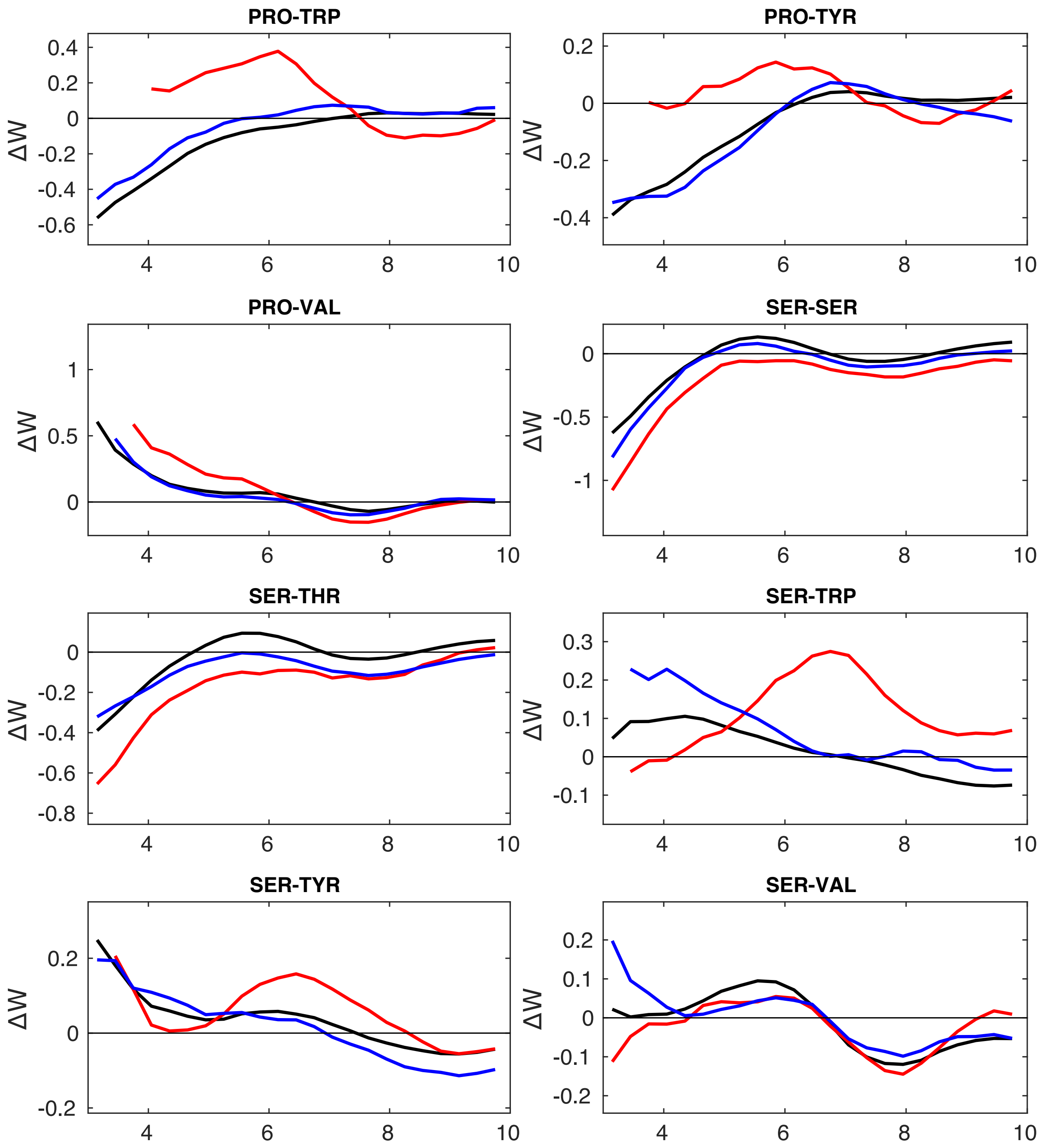
PRO-TRP
PRO-TYR
PRO-VAL
SER-SER
SER-THR
SER-TRP
SER-TYR
SER-VAL
ΔW
4
6
8
10

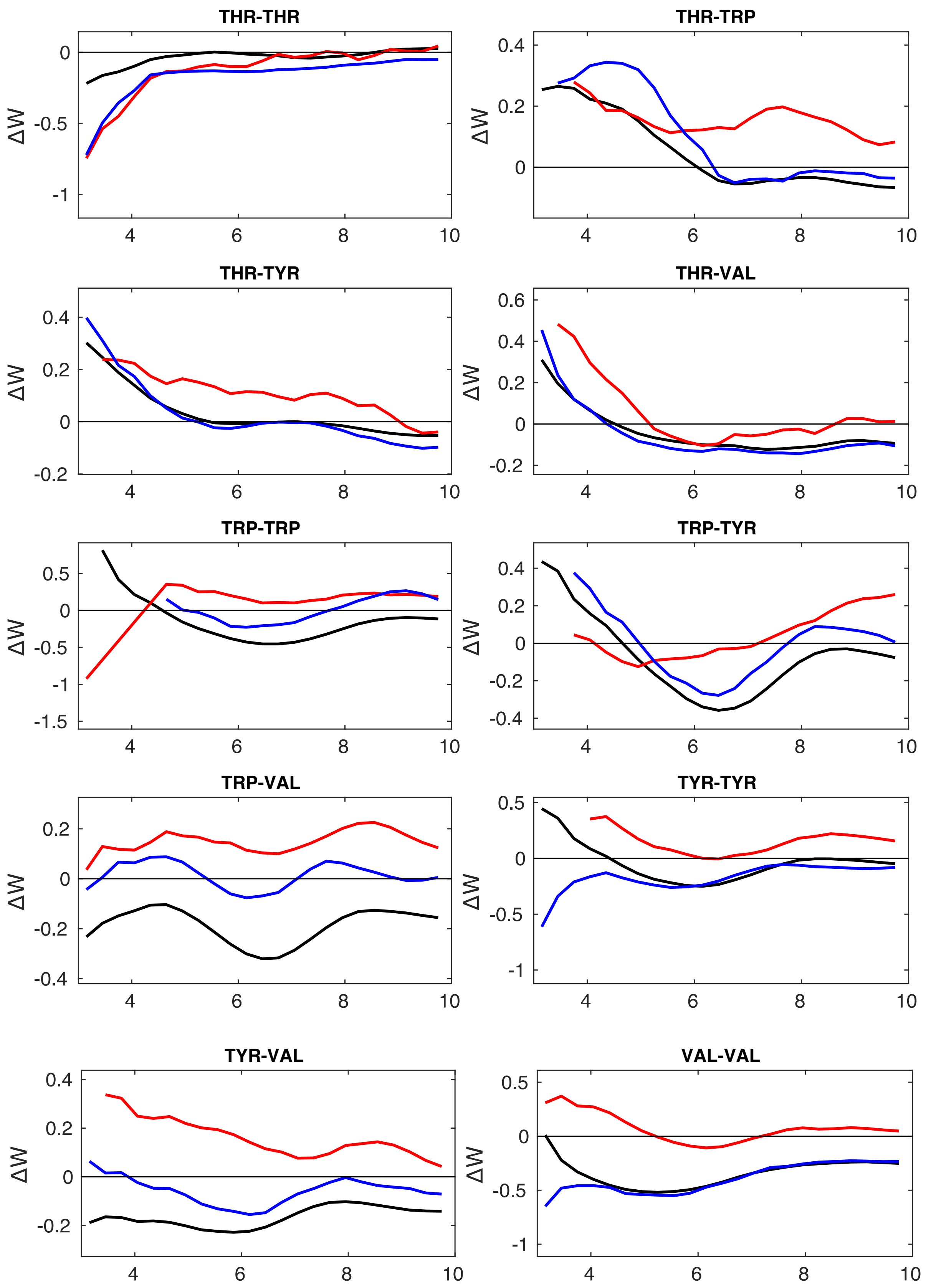
THR-THR
ΔW
0
-0.5
-1
4
6
8
10
THR-TRP
ΔW
0.4
0.2
0
4
6
8
10
THR-TYR
ΔW
0.4
0.2
0
-0.2
4
6
8
10
THR-VAL
ΔW
0.6
0.4
0.2
0
-0.2
4
6
8
10
TRP-TRP
ΔW
0.5
0
-0.5
-1
-1.5
4
6
8
10
TRP-TYR
ΔW
0.4
0.2
0
-0.2
-0.4
4
6
8
10
TRP-VAL
ΔW
0.2
0
-0.2
-0.4
4
6
8
10
TYR-TYR
ΔW
0.5
0
-0.5
-1
4
6
8
10
TYR-VAL
ΔW
0.4
0.2
0
-0.2
4
6
8
10
VAL-VAL
ΔW
0.5
0
-0.5
-1
4
6
8
10

Figure S4. Statistical "sds" potentials between amino acid groups as a function of the distance (in Å), derived from the datasets $\mathcal{D}^{EM}$ (blue lines), $\mathcal{D}^{TM}$ (red lines) and $\mathcal{D}^{GL}$ (dashed black lines). The numerical values are given in the Supplementary file Figure_S4.csv.

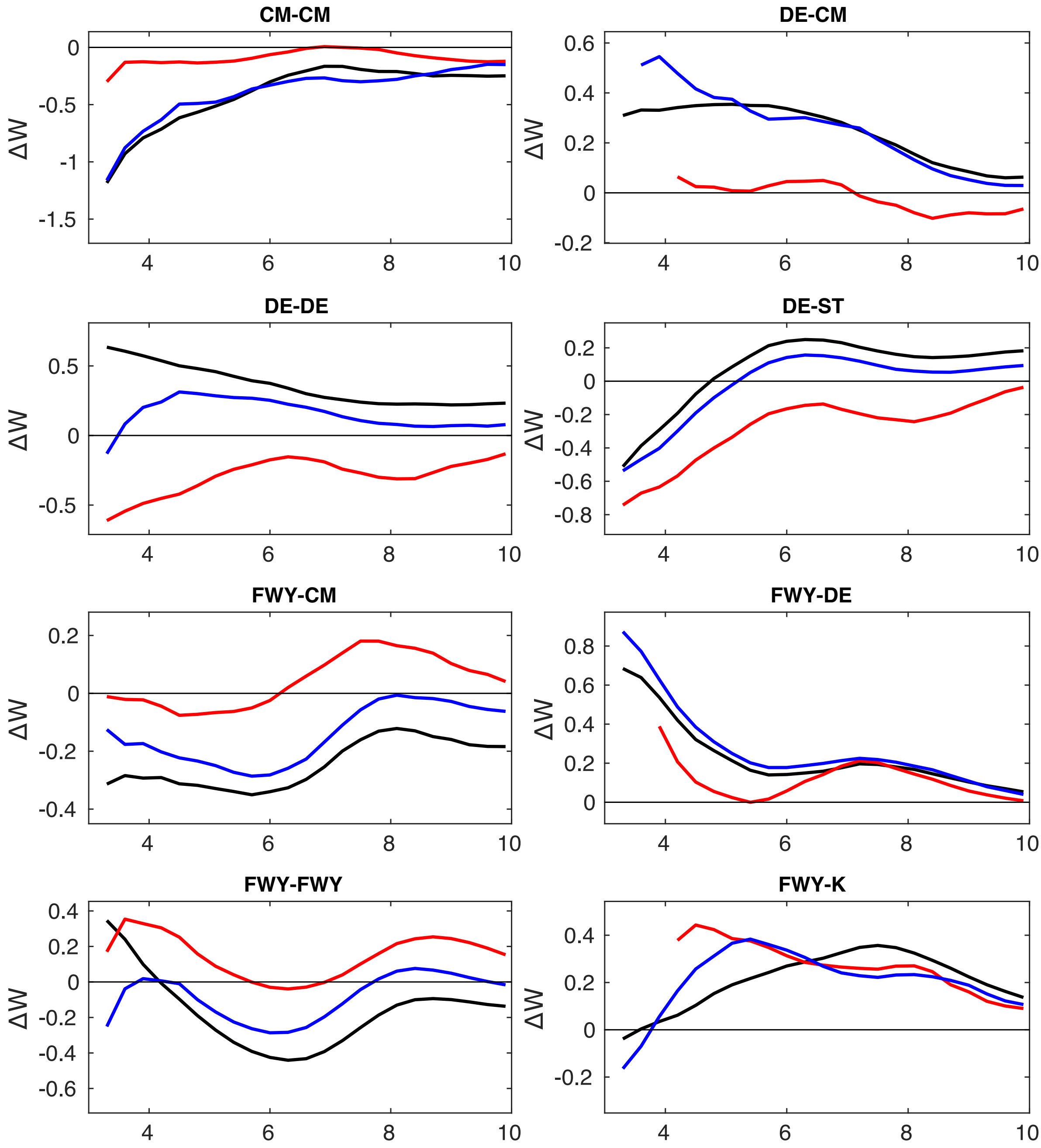

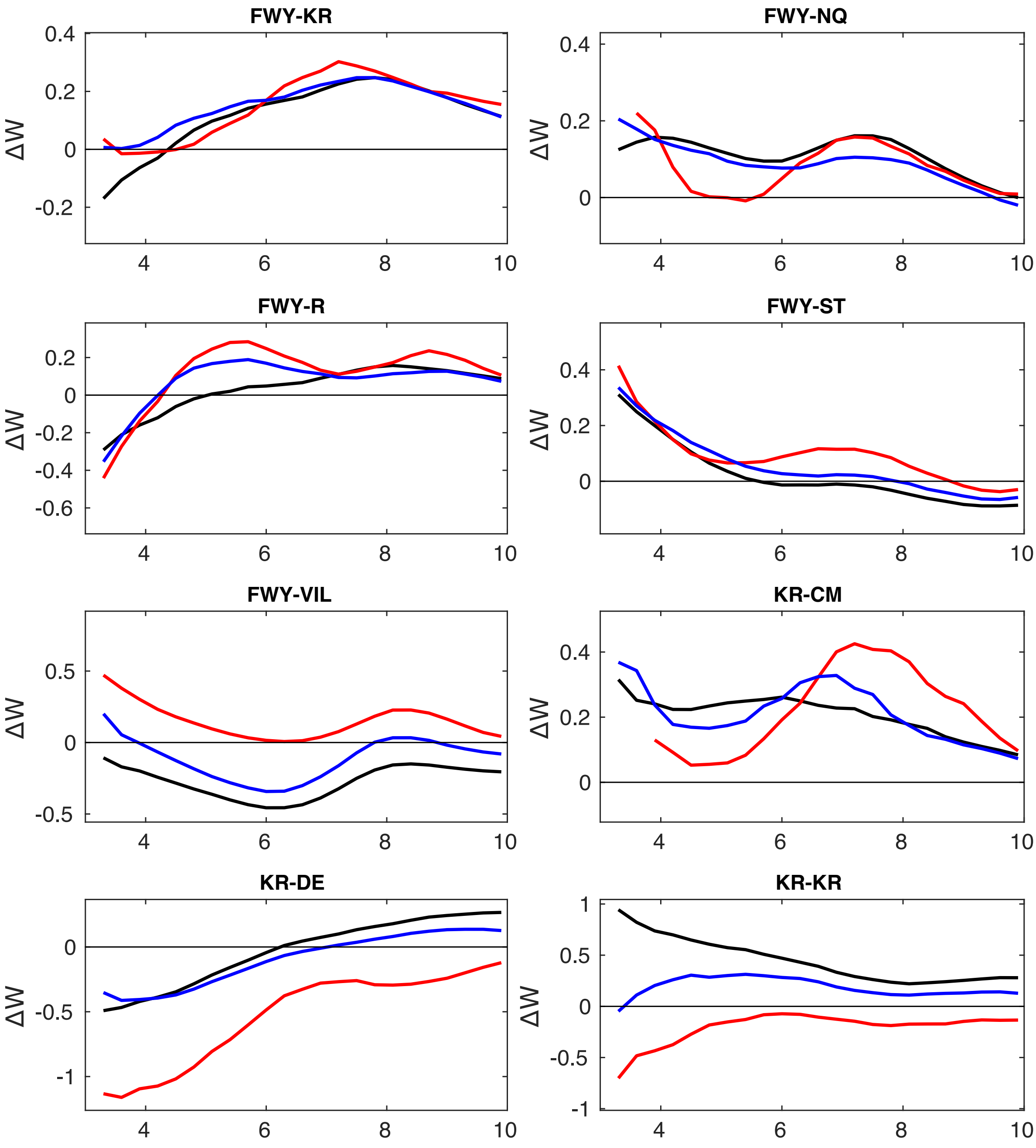
FWY-KR
FWY-NQ
FWY-R
FWY-ST
FWY-VIL
KR-CM
KR-DE
KR-KR
ΔW

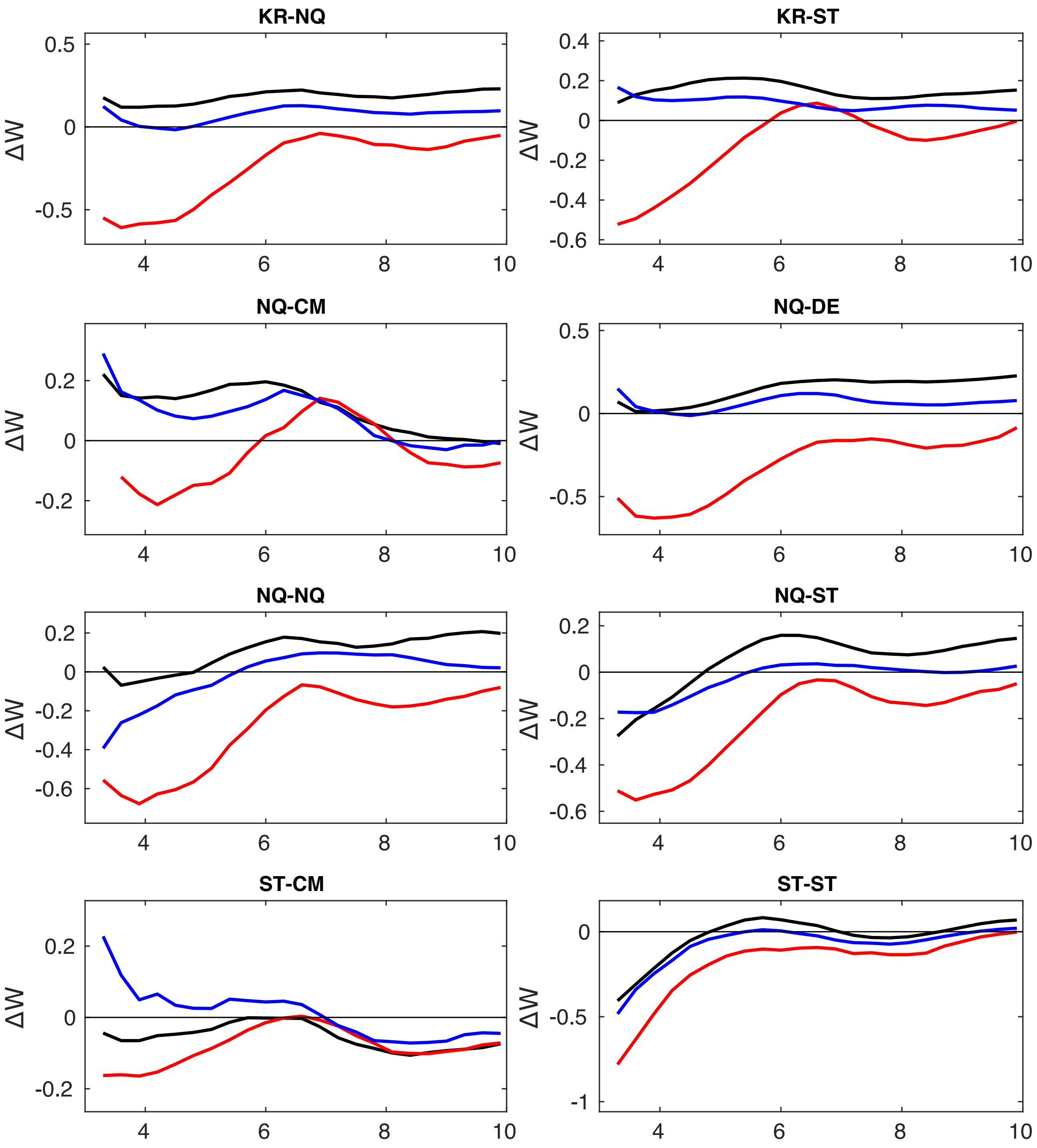
KR-NQ
KR-ST
NQ-CM
NQ-DE
NQ-NQ
NQ-ST
ST-CM
ST-ST
ΔW

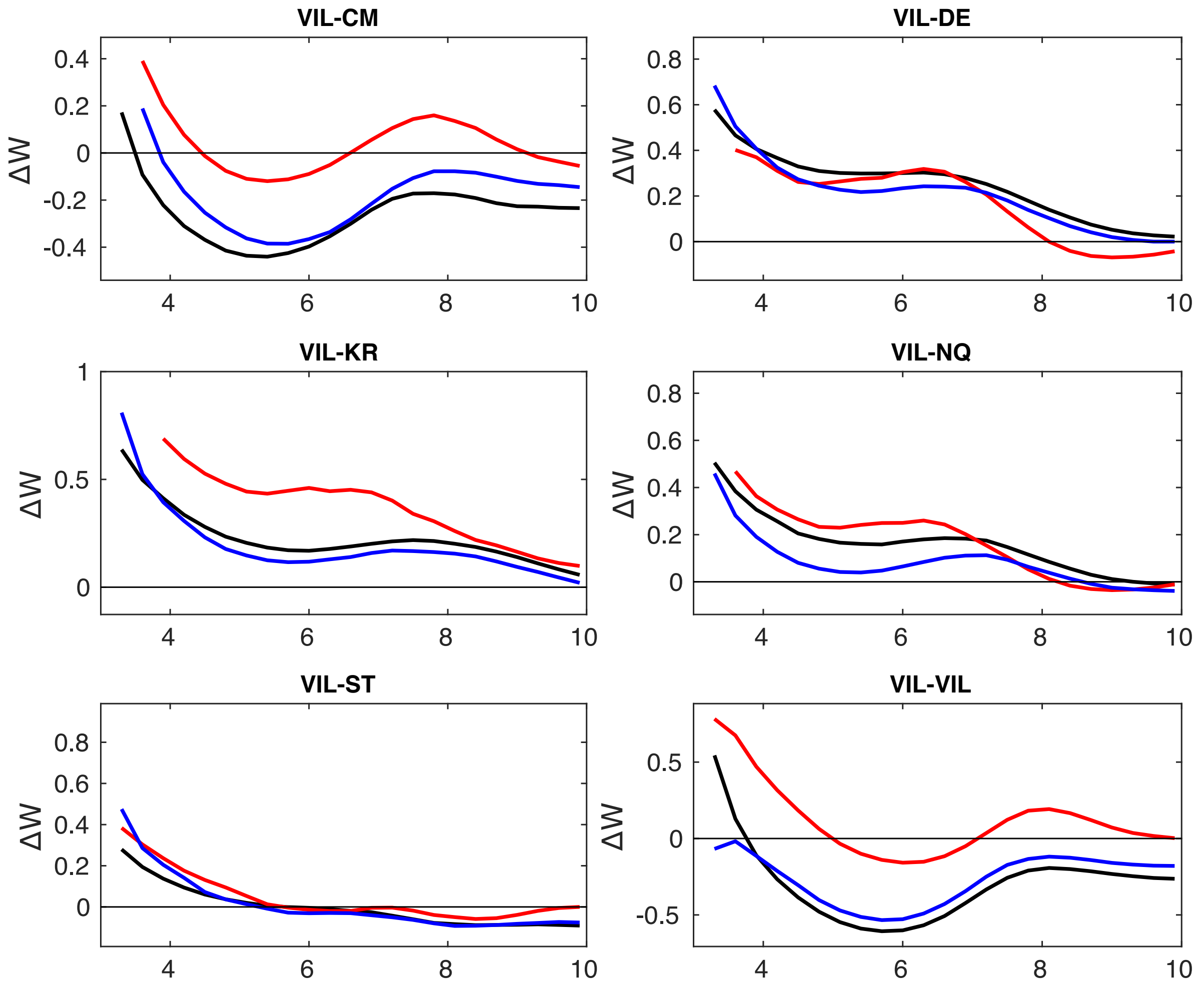
VIL-CM
VIL-DE
VIL-KR
VIL-NQ
VIL-ST
VIL-VIL
ΔW
0.4
0.2
0
-0.2
-0.4
0.8
0.6
1
0.5
-0.5
4
6
8
10

Figure S5. Statistical “sds” residue-residue potentials as a function of the distance (in Å), derived from the dataset $\mathcal{D}^{TM}$ (red lines), or separately from α-helical (orange lines) and β-barrel (magenta lines) transmembrane regions. The numerical values are given in the Supplementary file Figure_S5.csv.

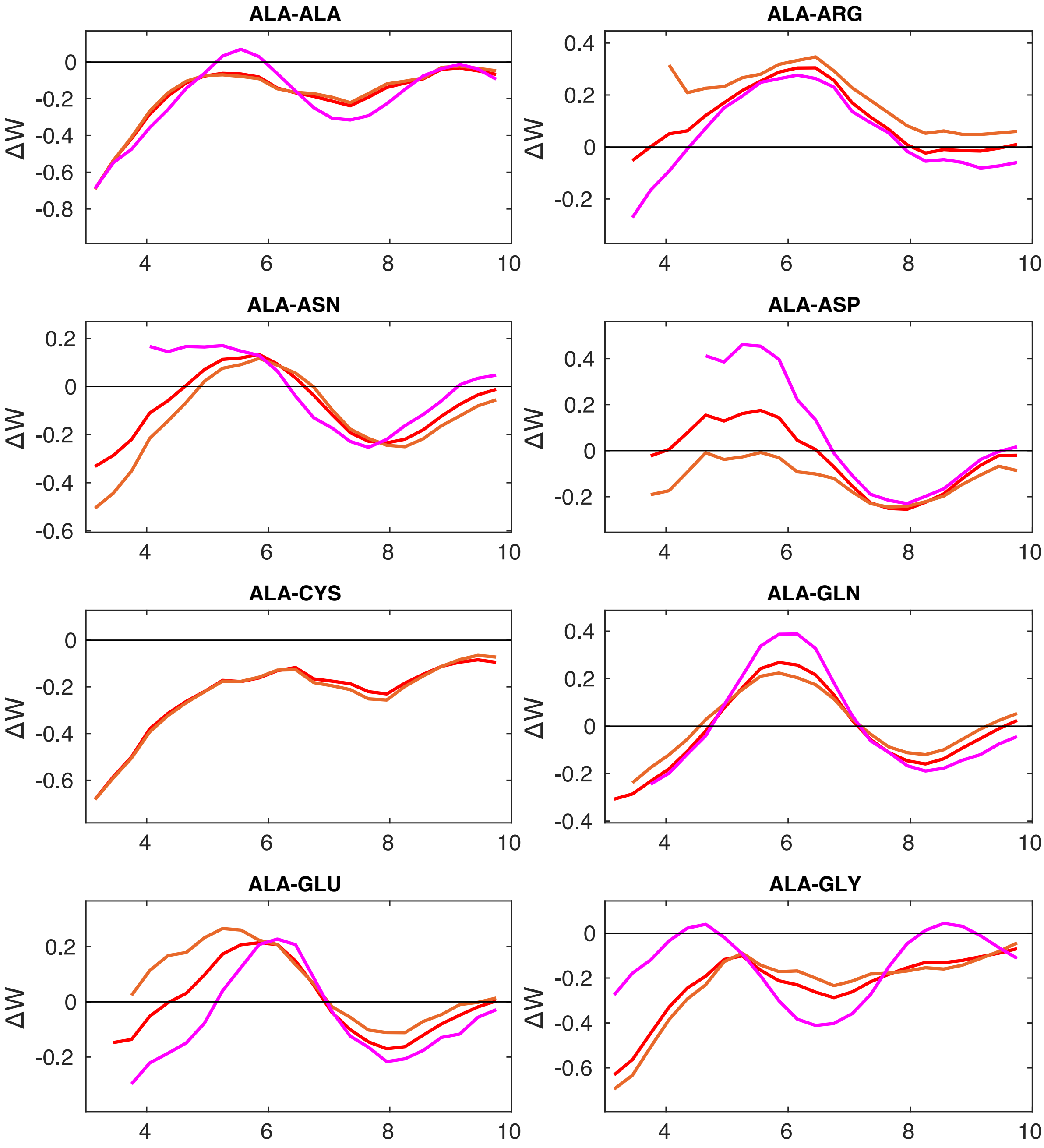

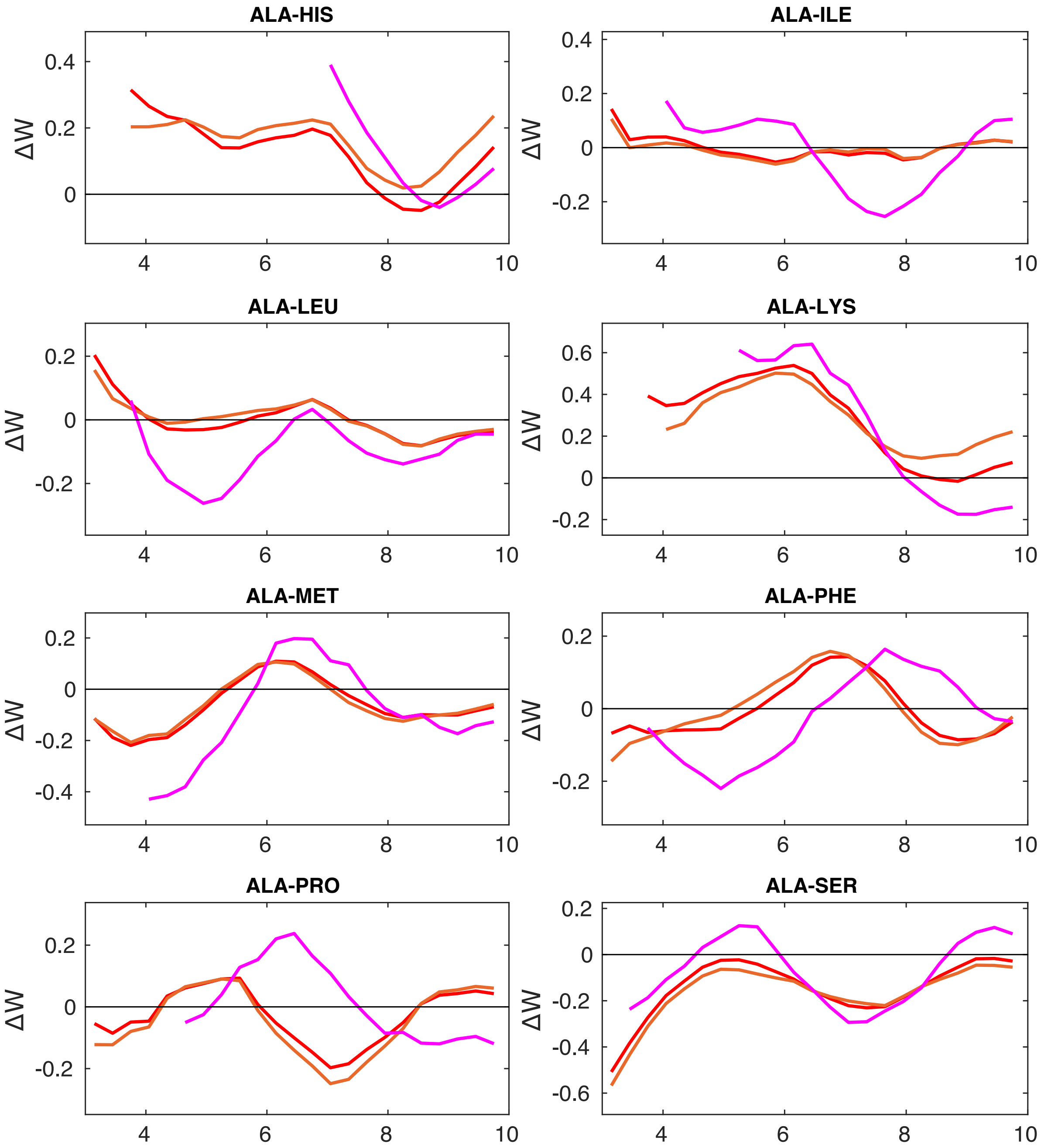
ALA-HIS
ALA-ILE
ALA-LEU
ALA-LYS
ALA-MET
ALA-PHE
ALA-PRO
ALA-SER
ΔW
4
6
8
10

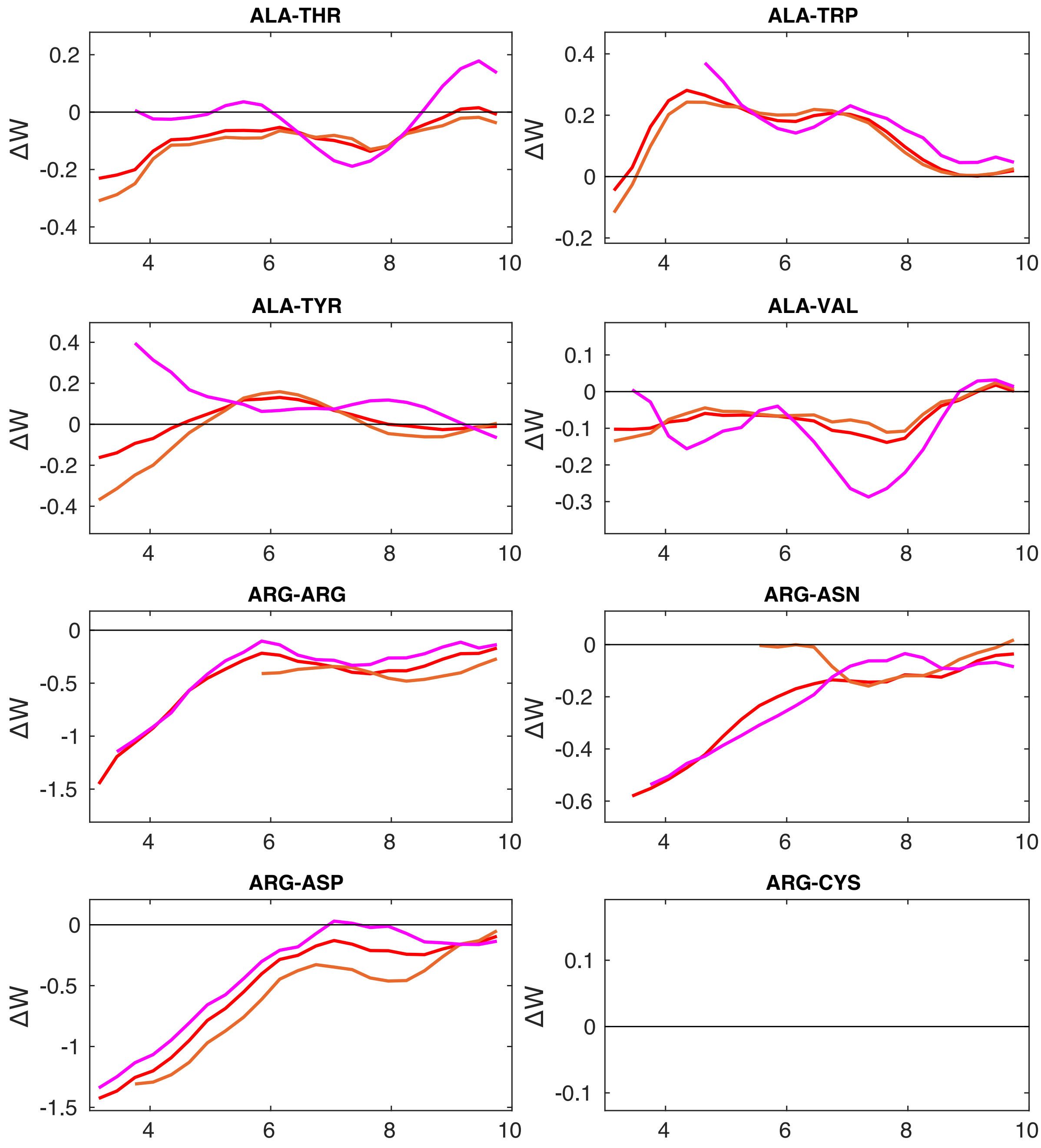
ALA-THR
ALA-TRP
ALA-TYR
ALA-VAL
ARG-ARG
ARG-ASN
ARG-ASP
ARG-CYS
ΔW
0.2
0
-0.2
-0.4
0.4
0.1
-0.1
-0.3
-0.5
-1
-1.5
-0.6
4
6
8
10

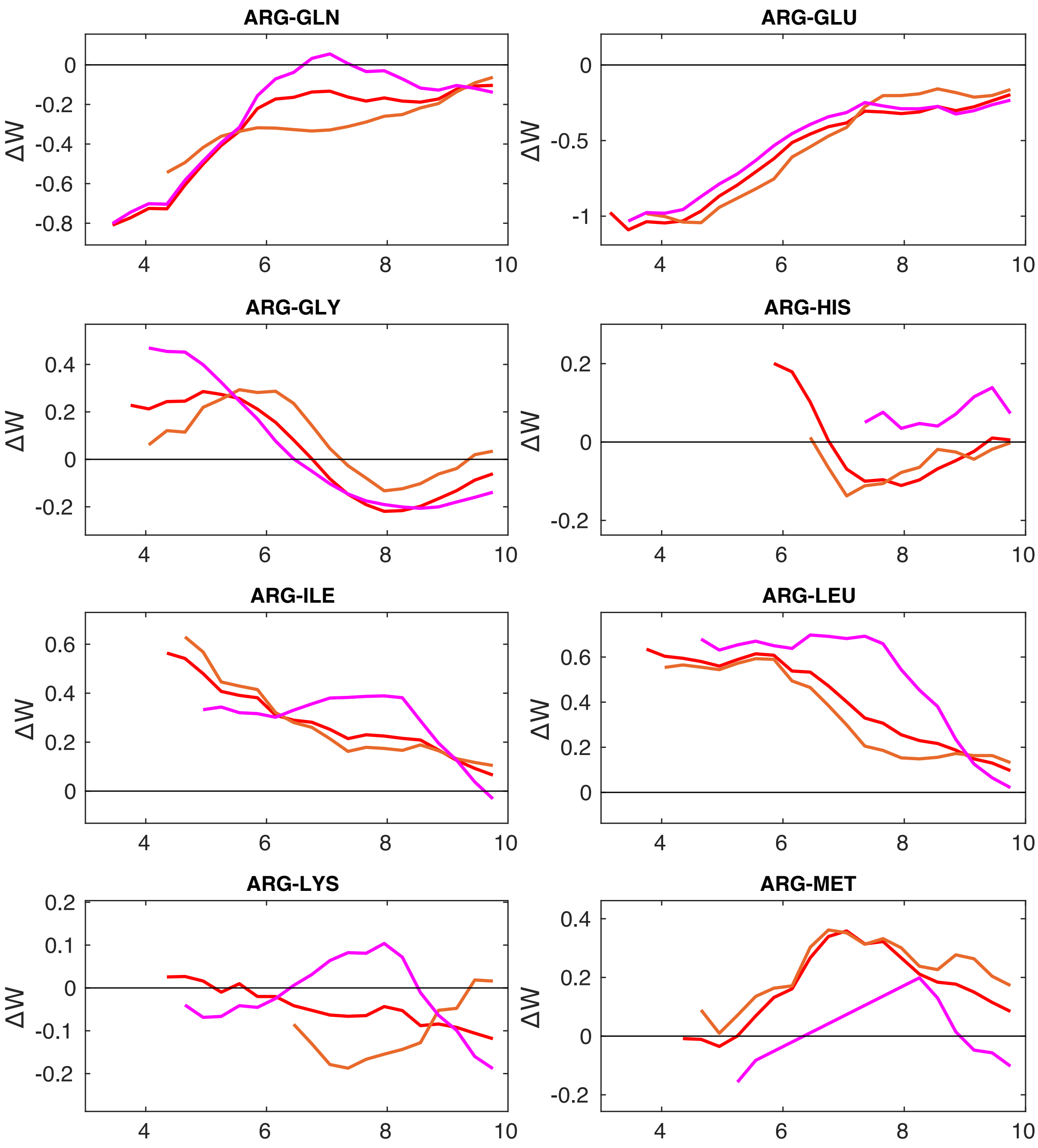
ARG-GLN
ARG-GLU
ARG-GLY
ARG-HIS
ARG-ILE
ARG-LEU
ARG-LYS
ARG-MET
ΔW

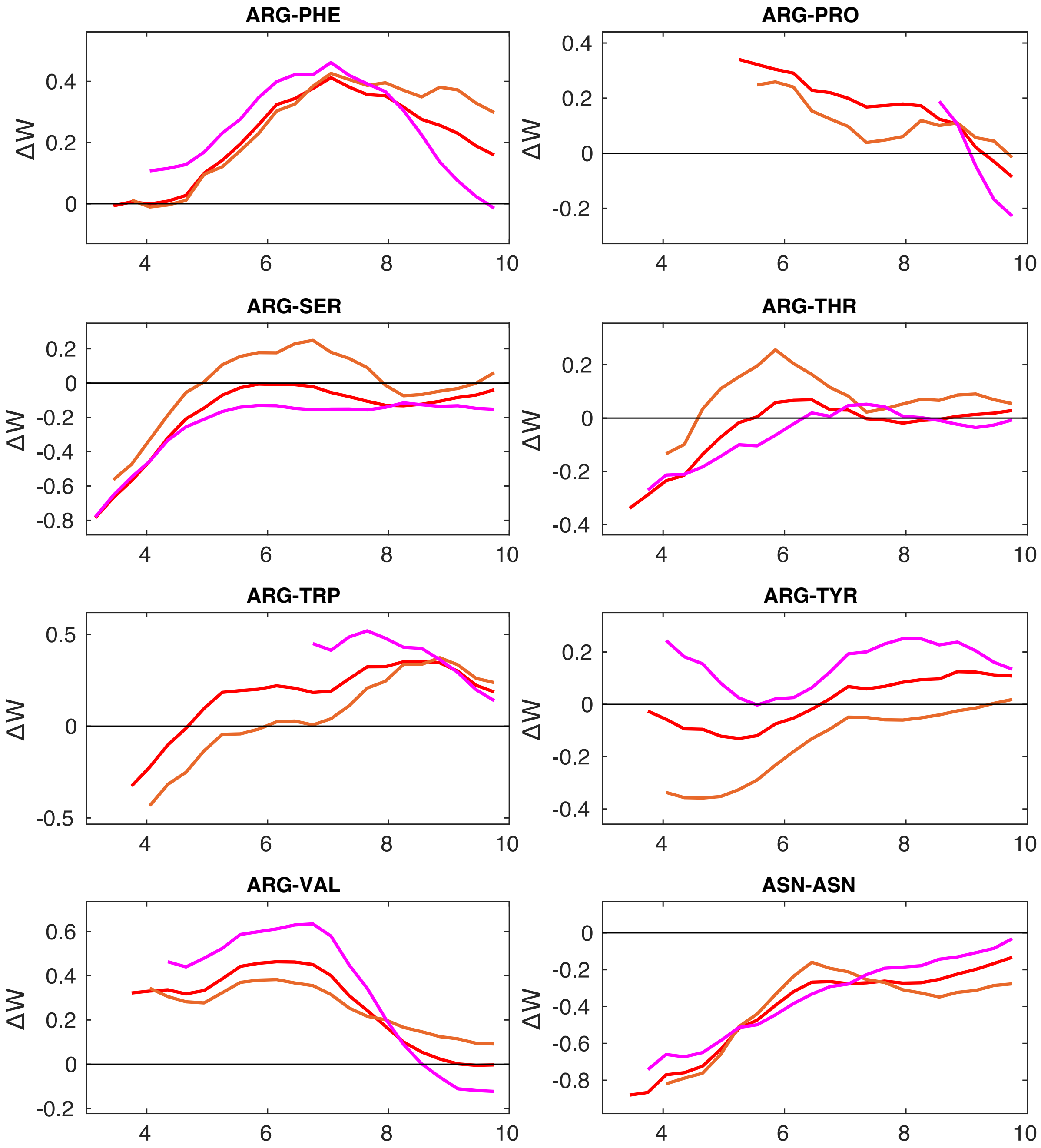
ARG-PHE
ARG-PRO
ARG-SER
ARG-THR
ARG-TRP
ARG-TYR
ARG-VAL
ASN-ASN
ΔW
4
6
8
10

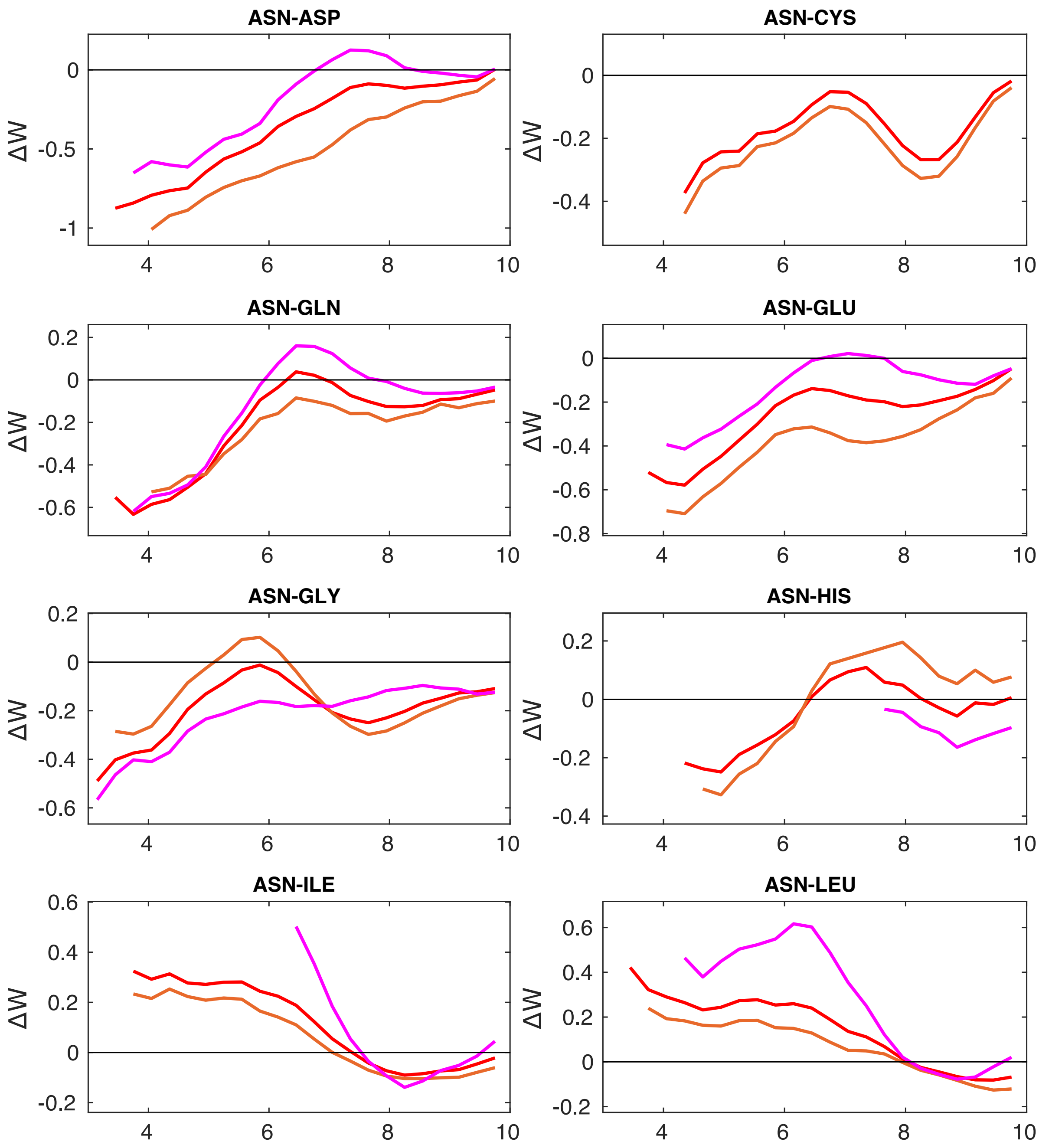
ASN-ASP
ASN-CYS
ASN-GLN
ASN-GLU
ASN-GLY
ASN-HIS
ASN-ILE
ASN-LEU
ΔW

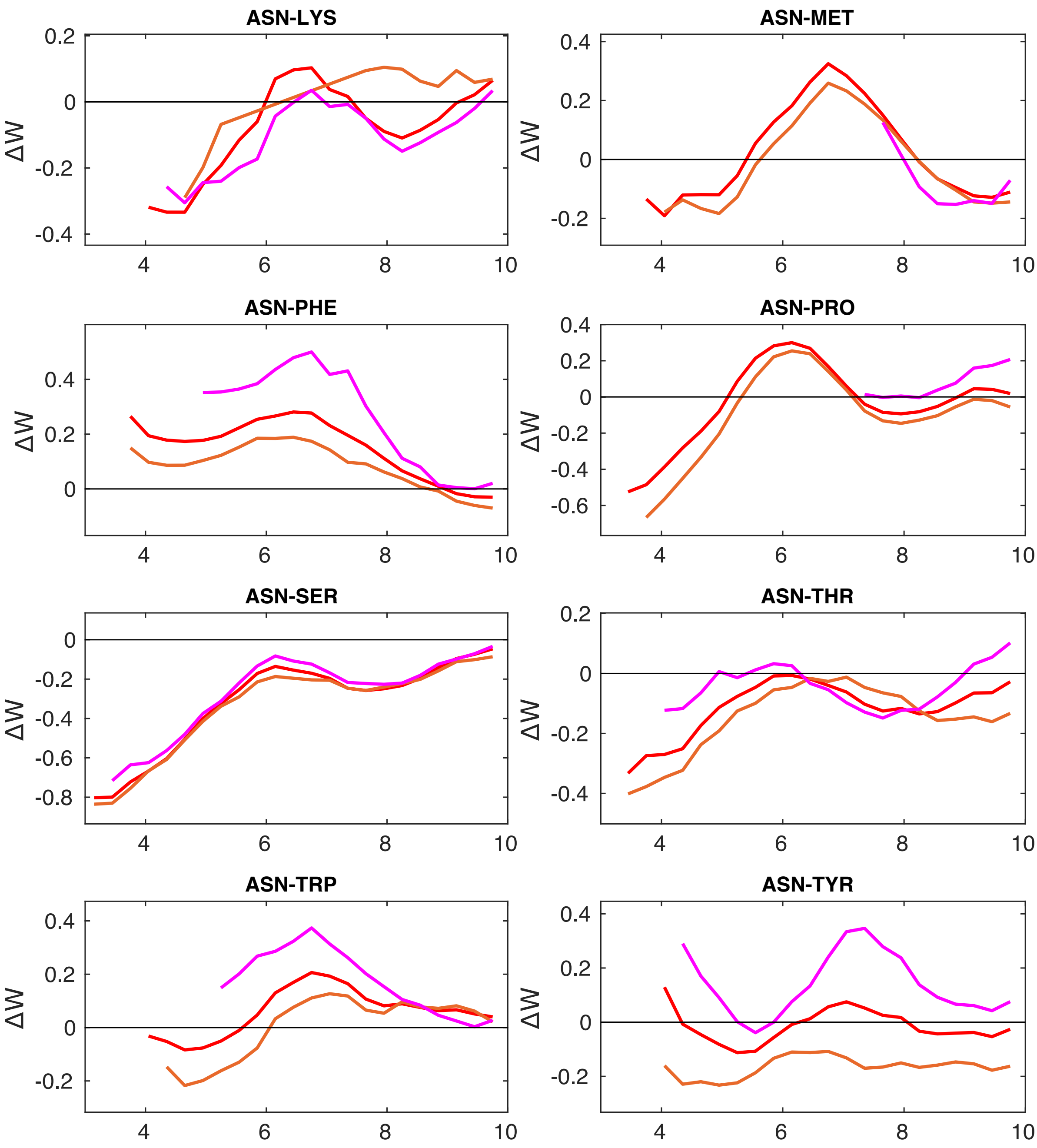
ASN-LYS
ASN-MET
ASN-PHE
ASN-PRO
ASN-SER
ASN-THR
ASN-TRP
ASN-TYR
ΔW
4
6
8
10

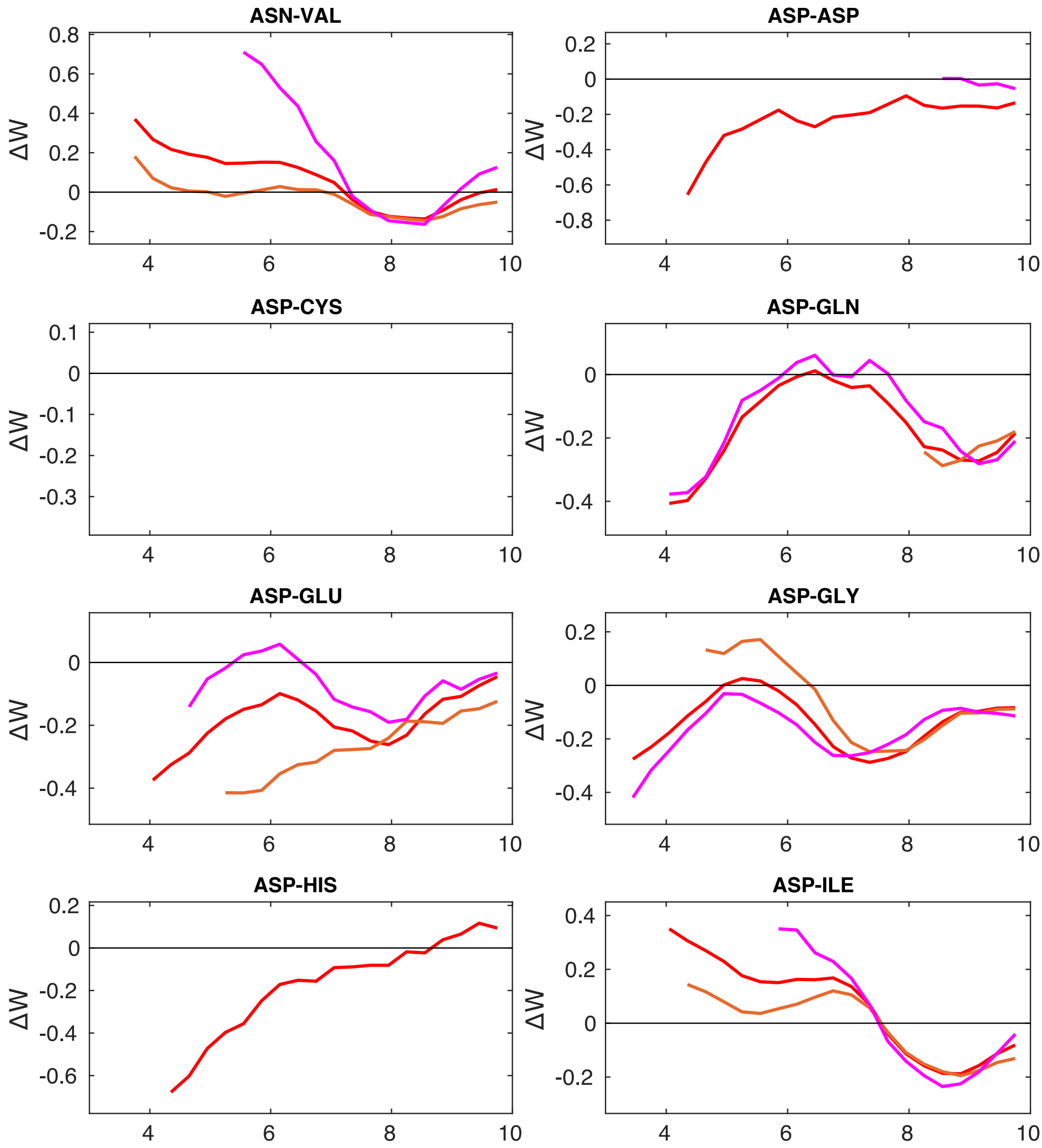
ASN-VAL
ASP-ASP
ASP-CYS
ASP-GLN
ASP-GLU
ASP-GLY
ASP-HIS
ASP-ILE
ΔW

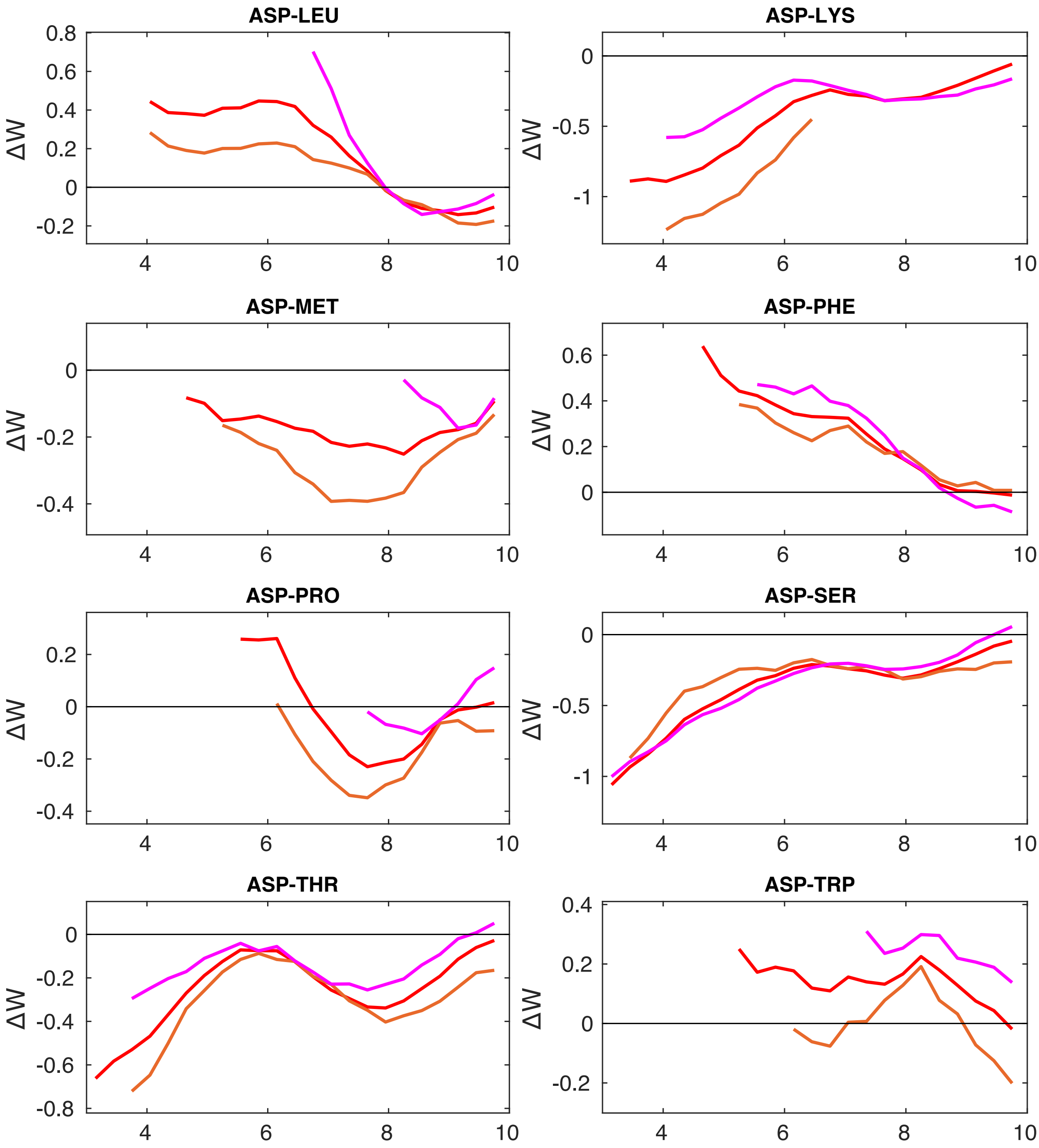
ASP-LEU
ASP-LYS
ASP-MET
ASP-PHE
ASP-PRO
ASP-SER
ASP-THR
ASP-TRP
ΔW

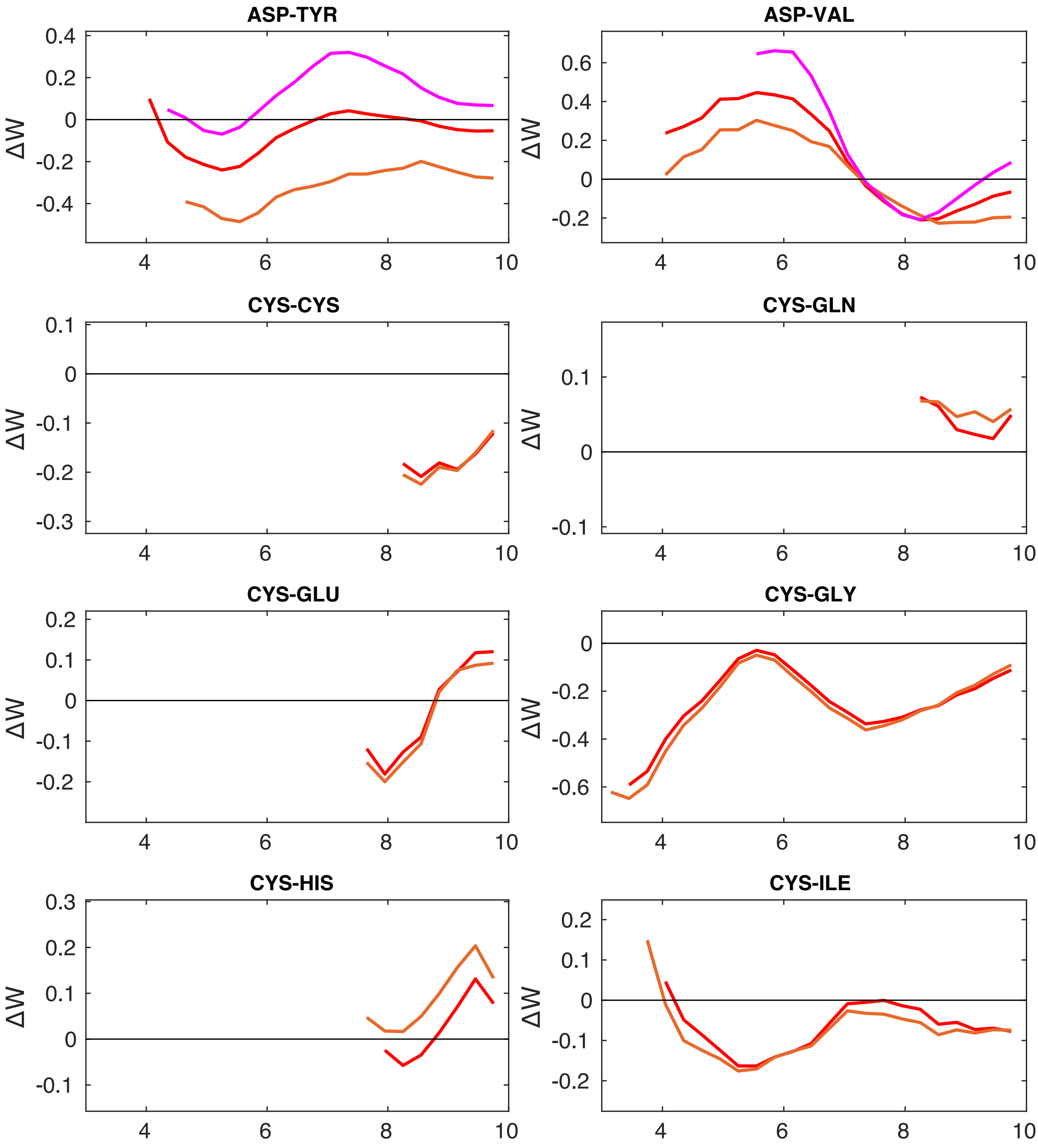
ASP-TYR
ASP-VAL
CYS-CYS
CYS-GLN
CYS-GLU
CYS-GLY
CYS-HIS
CYS-ILE
ΔW
4
6
8
10

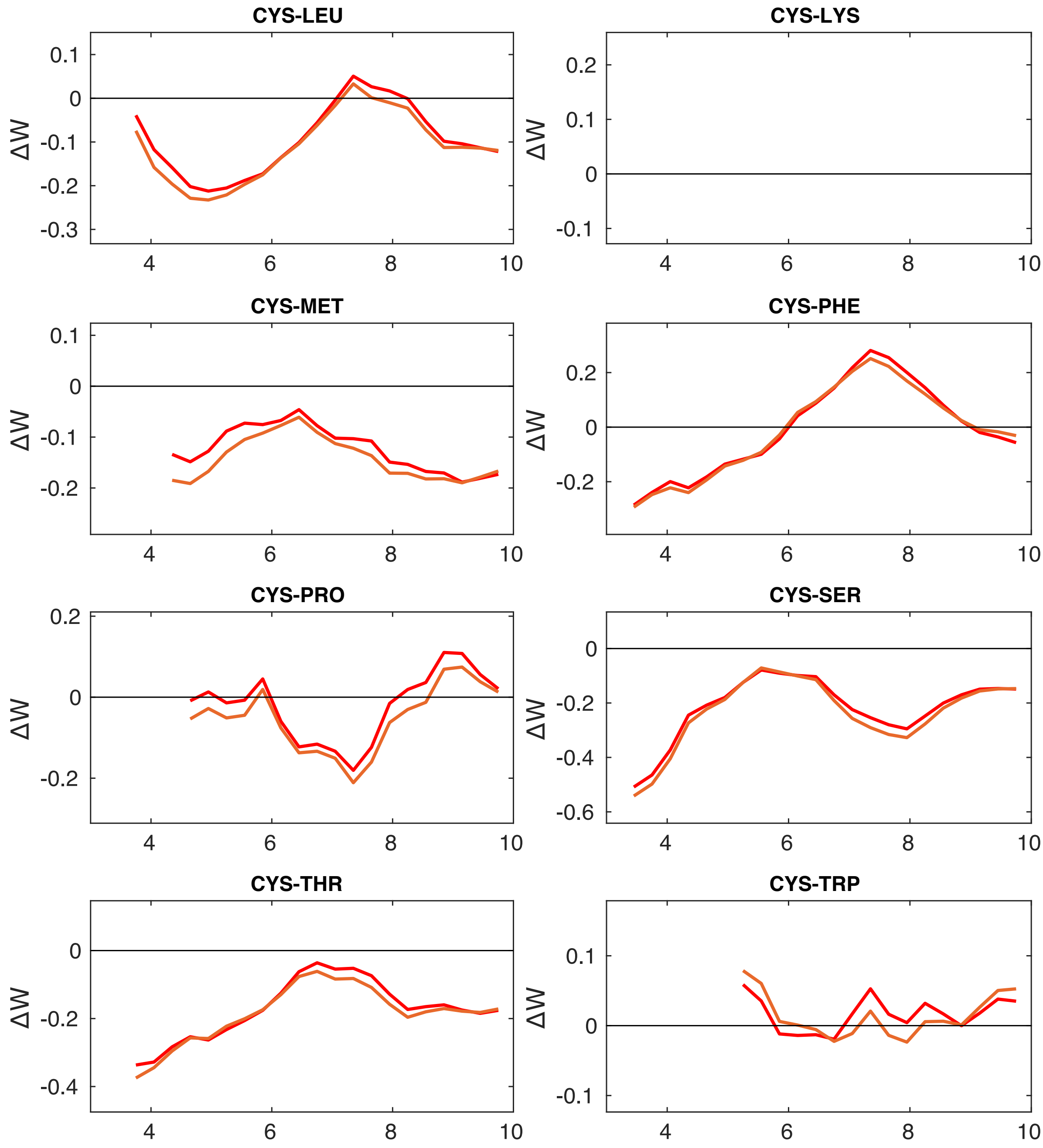
CYS-LEU
CYS-LYS
CYS-MET
CYS-PHE
CYS-PRO
CYS-SER
CYS-THR
CYS-TRP
ΔW
4
6
8
10

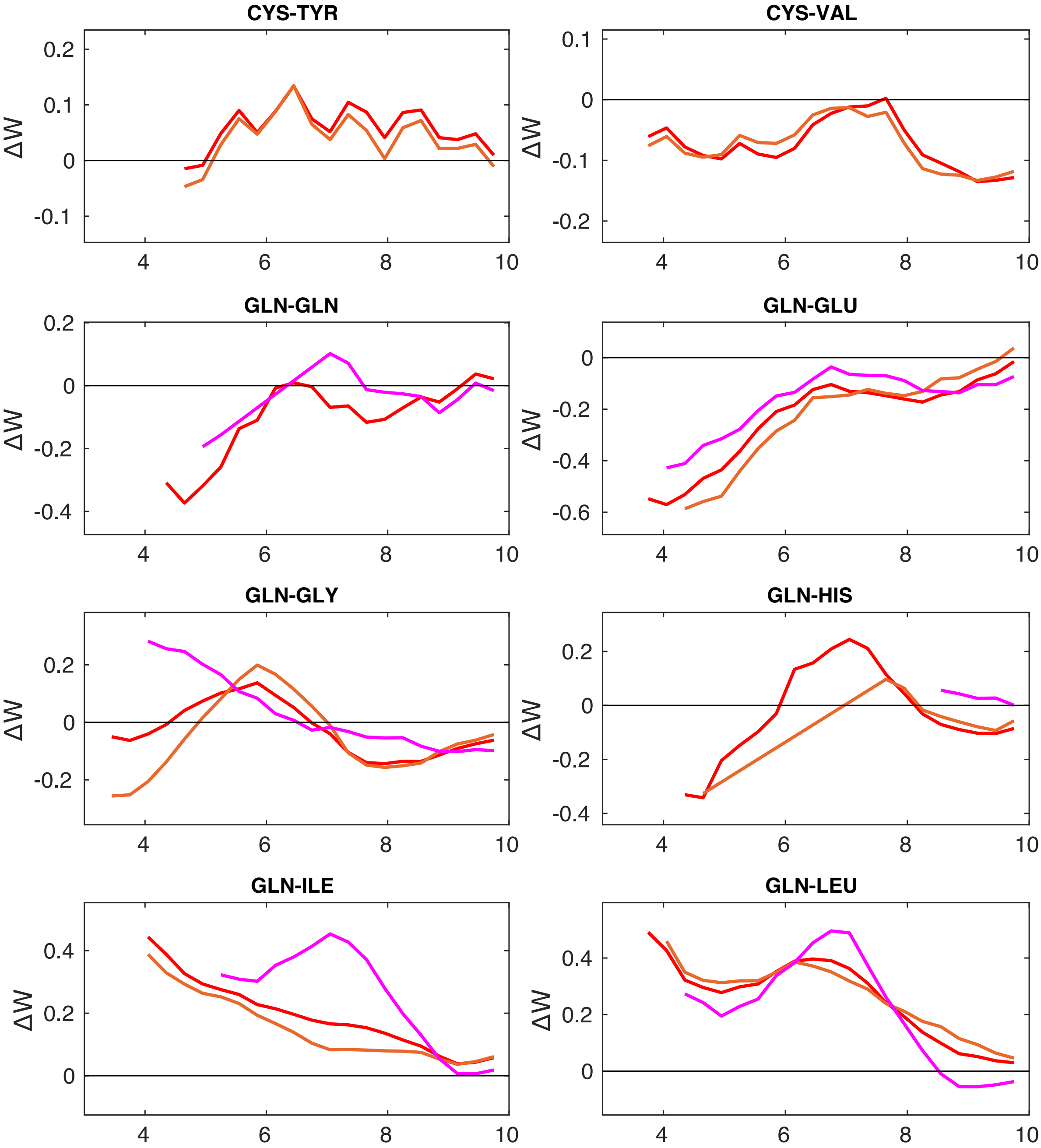
CYS-TYR
CYS-VAL
GLN-GLN
GLN-GLU
GLN-GLY
GLN-HIS
GLN-ILE
GLN-LEU
ΔW
4
6
8
10

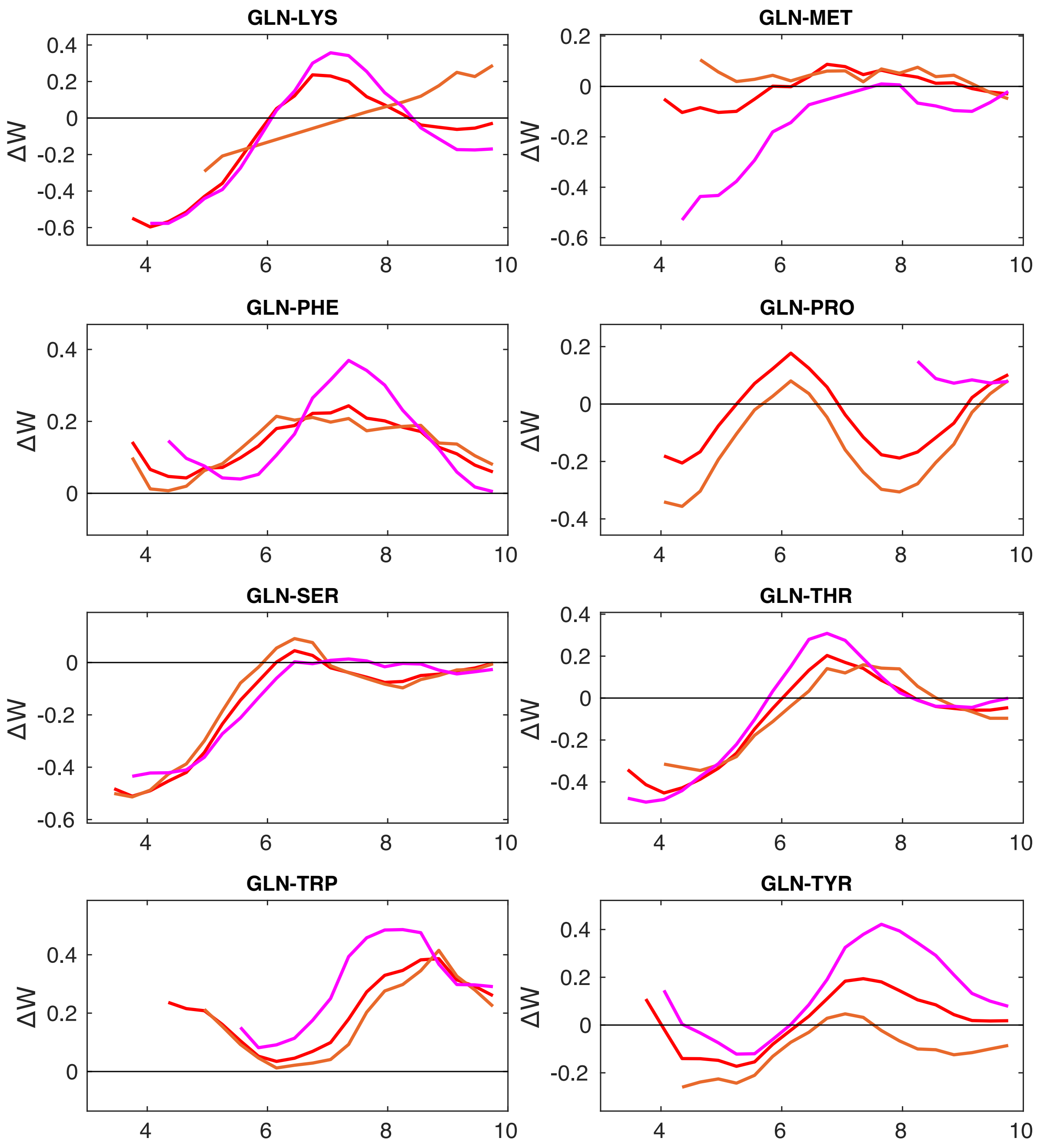
GLN-LYS
GLN-MET
GLN-PHE
GLN-PRO
GLN-SER
GLN-THR
GLN-TRP
GLN-TYR
ΔW

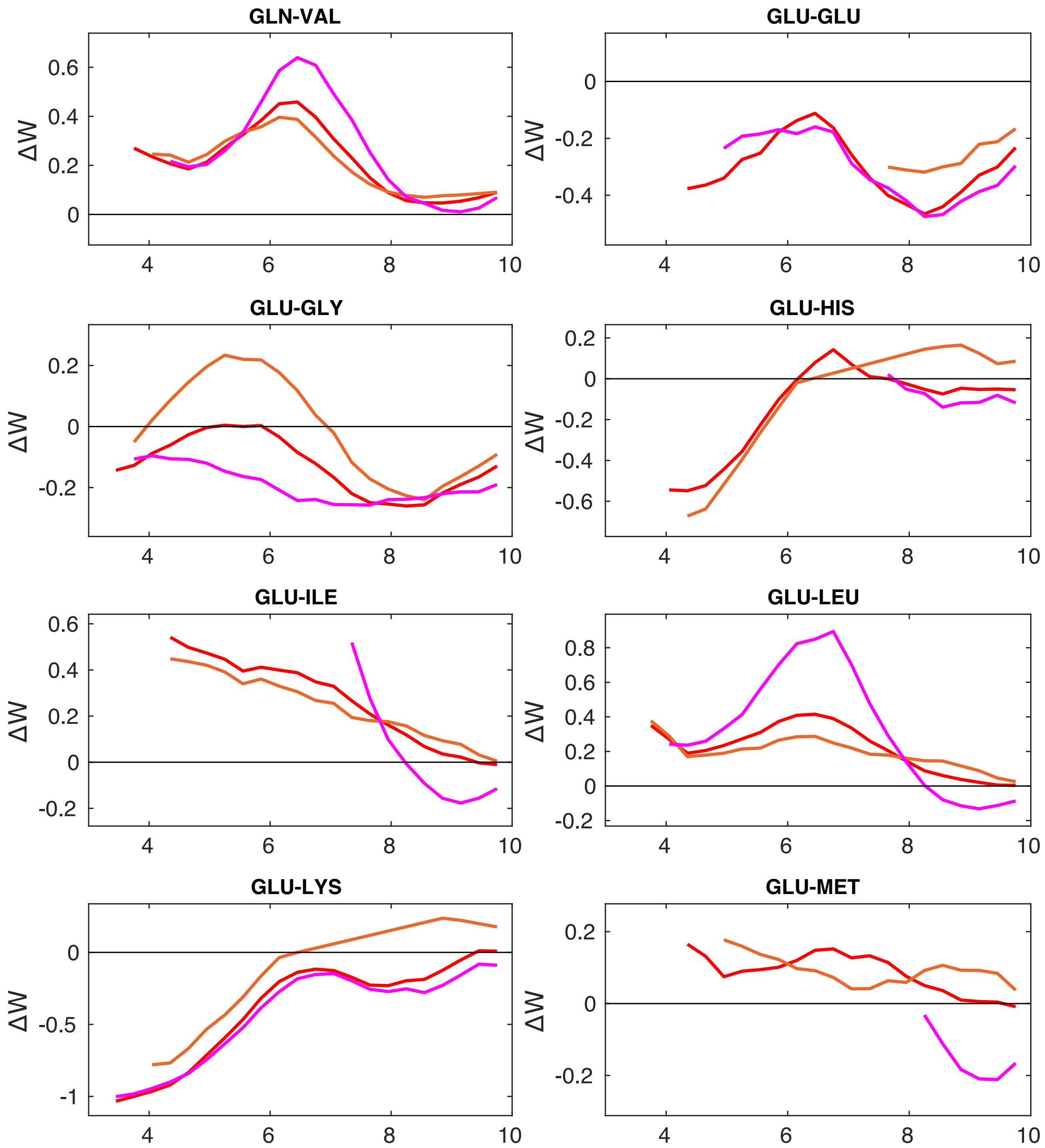
GLN-VAL
GLU-GLU
GLU-GLY
GLU-HIS
GLU-ILE
GLU-LEU
GLU-LYS
GLU-MET
ΔW

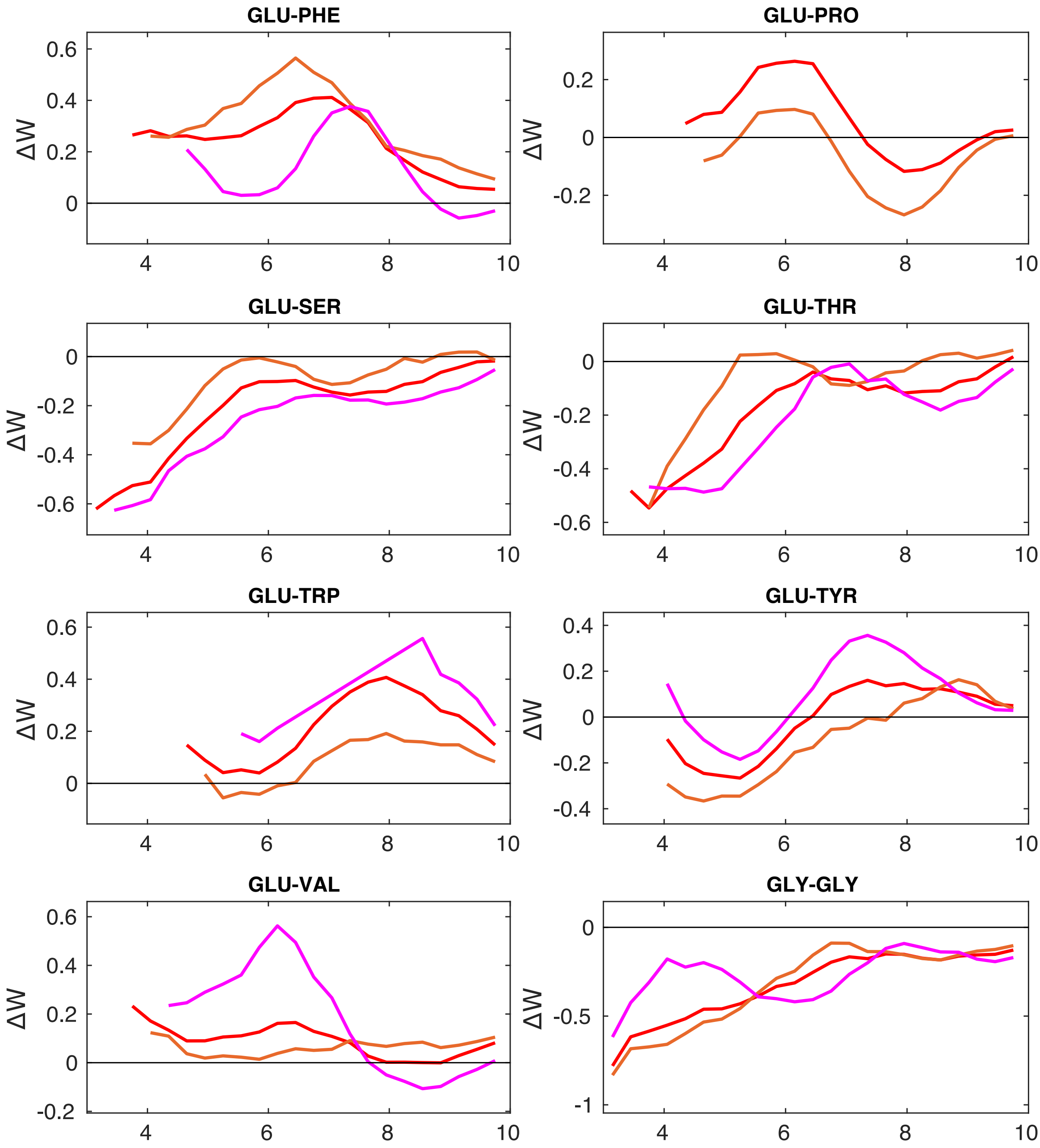
GLU-PHE
GLU-PRO
GLU-SER
GLU-THR
GLU-TRP
GLU-TYR
GLU-VAL
GLY-GLY
ΔW

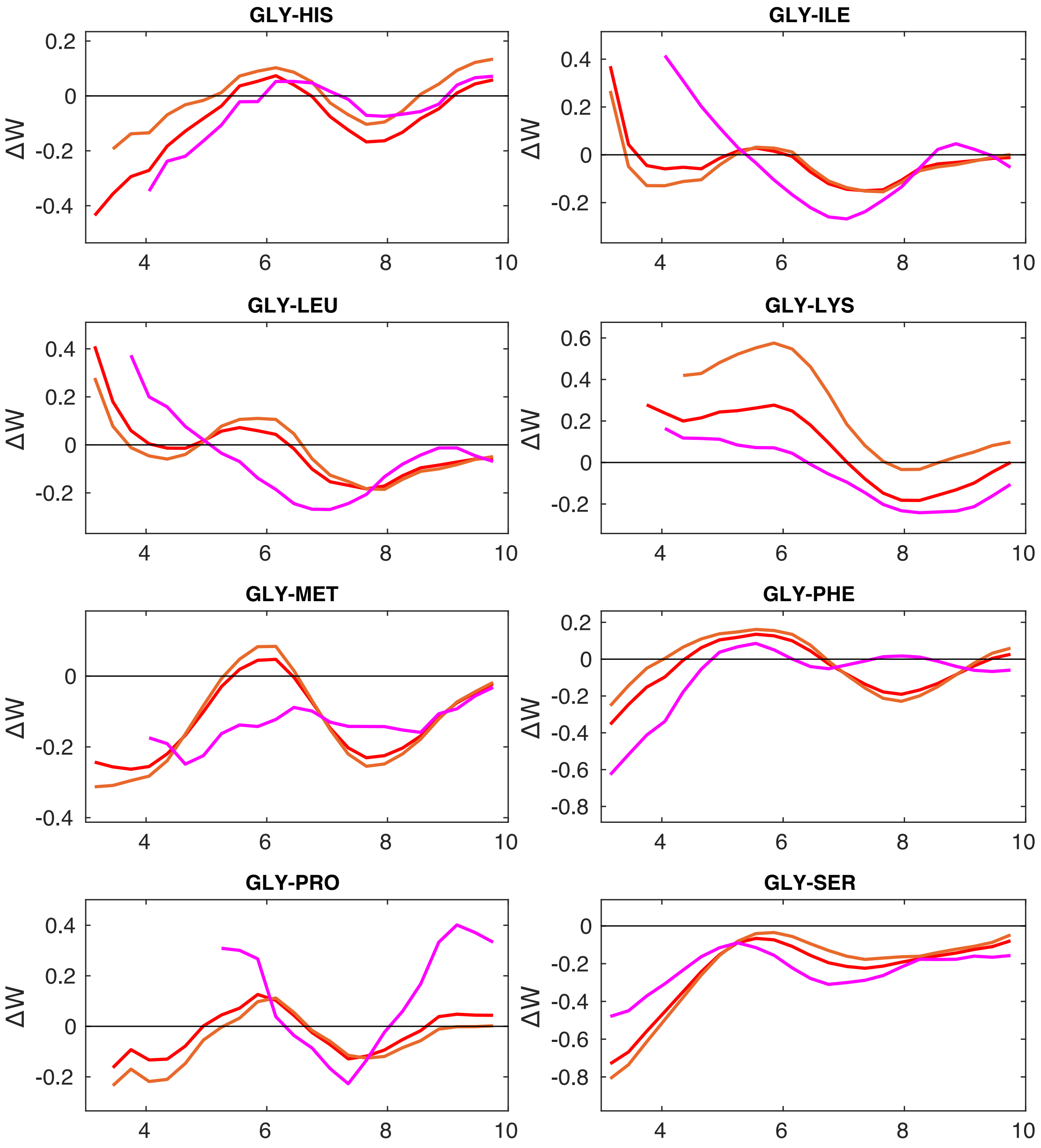
GLY-HIS
GLY-ILE
GLY-LEU
GLY-LYS
GLY-MET
GLY-PHE
GLY-PRO
GLY-SER
ΔW

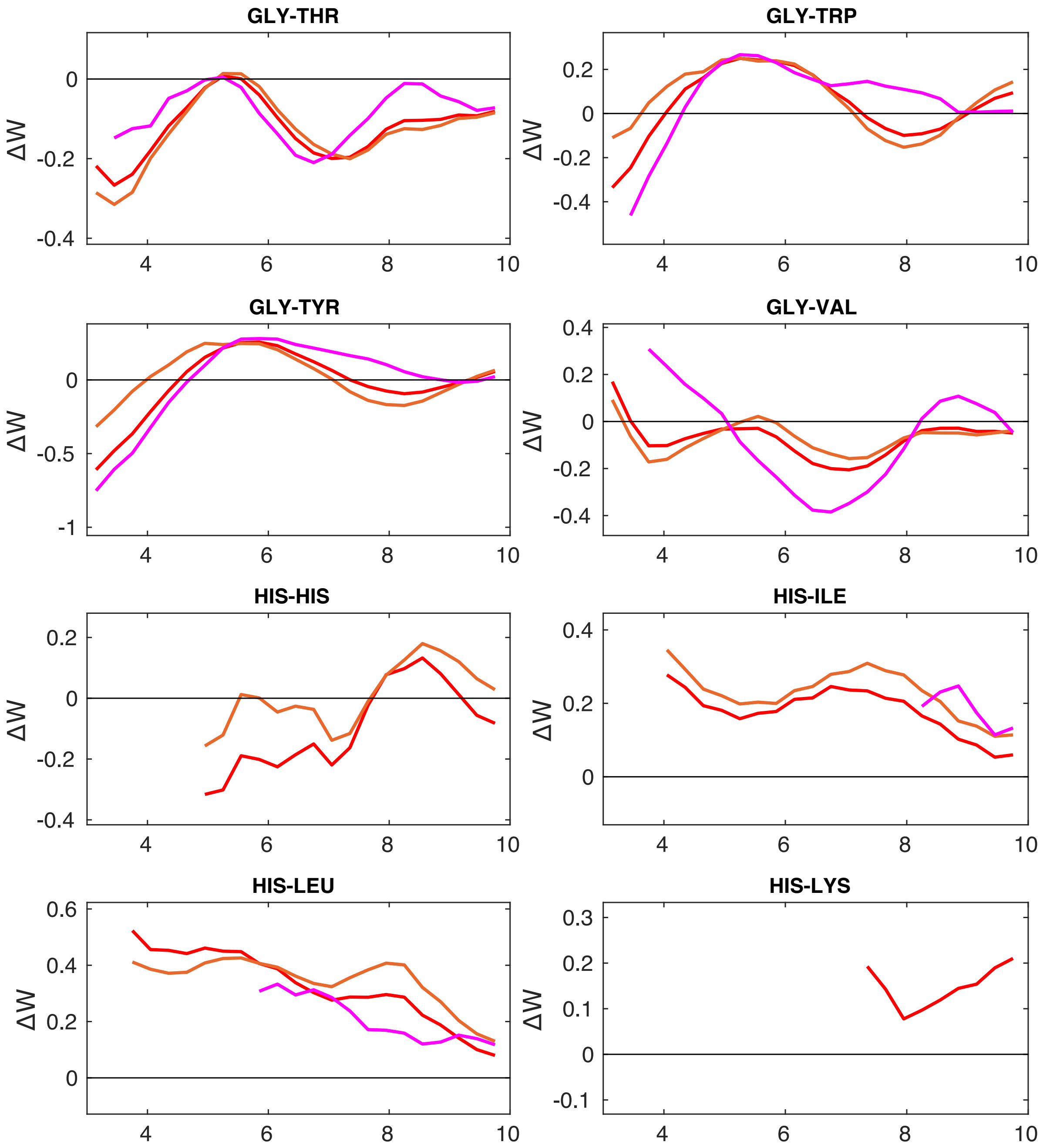
GLY-THR
GLY-TRP
GLY-TYR
GLY-VAL
HIS-HIS
HIS-ILE
HIS-LEU
HIS-LYS
ΔW
4
6
8
10

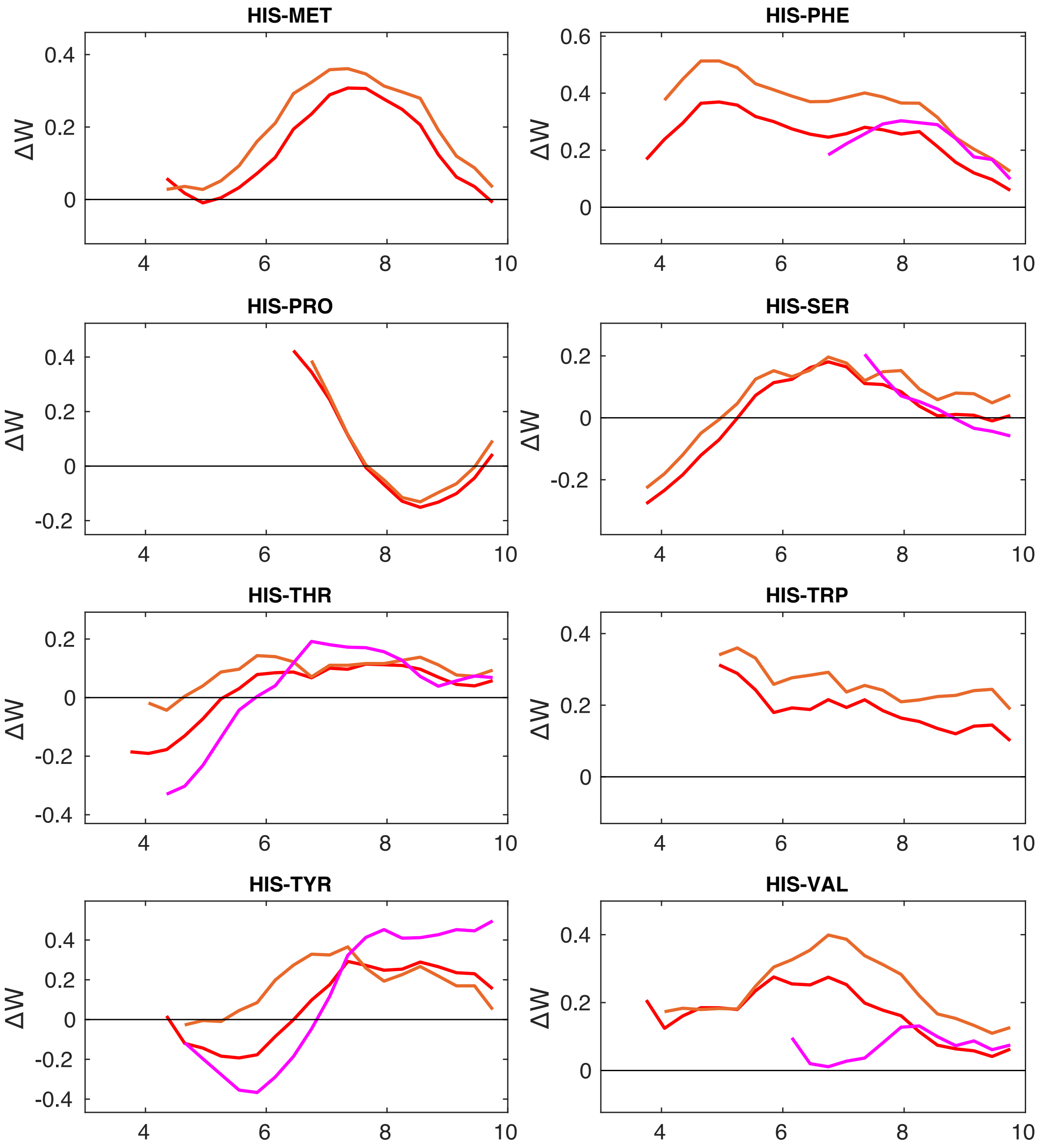
HIS-MET
HIS-PHE
HIS-PRO
HIS-SER
HIS-THR
HIS-TRP
HIS-TYR
HIS-VAL
ΔW
4
6
8
10

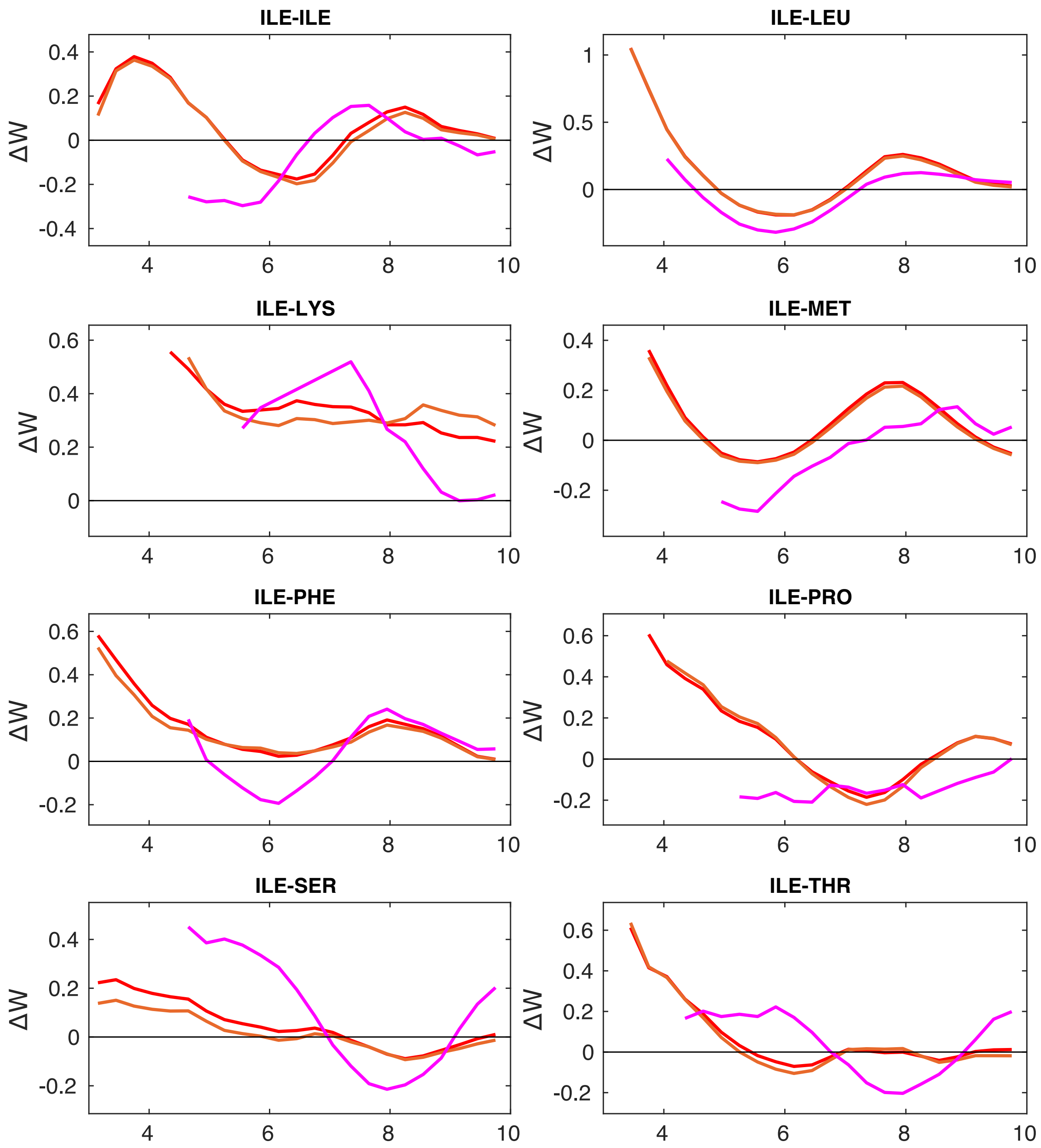
ILE-ILE
ILE-LEU
ILE-LYS
ILE-MET
ILE-PHE
ILE-PRO
ILE-SER
ILE-THR
ΔW

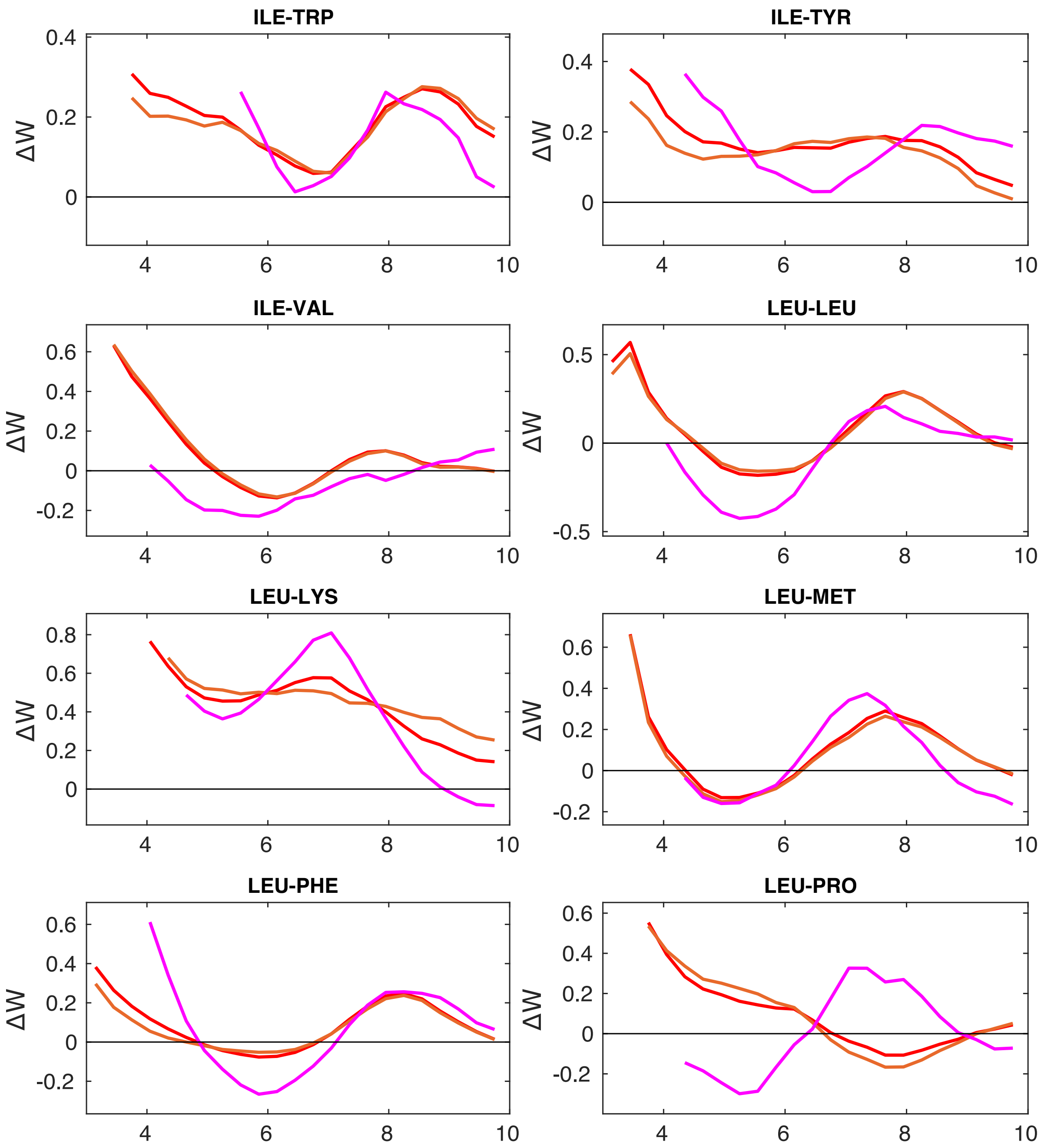
ILE-TRP
ILE-TYR
ILE-VAL
LEU-LEU
LEU-LYS
LEU-MET
LEU-PHE
LEU-PRO
ΔW

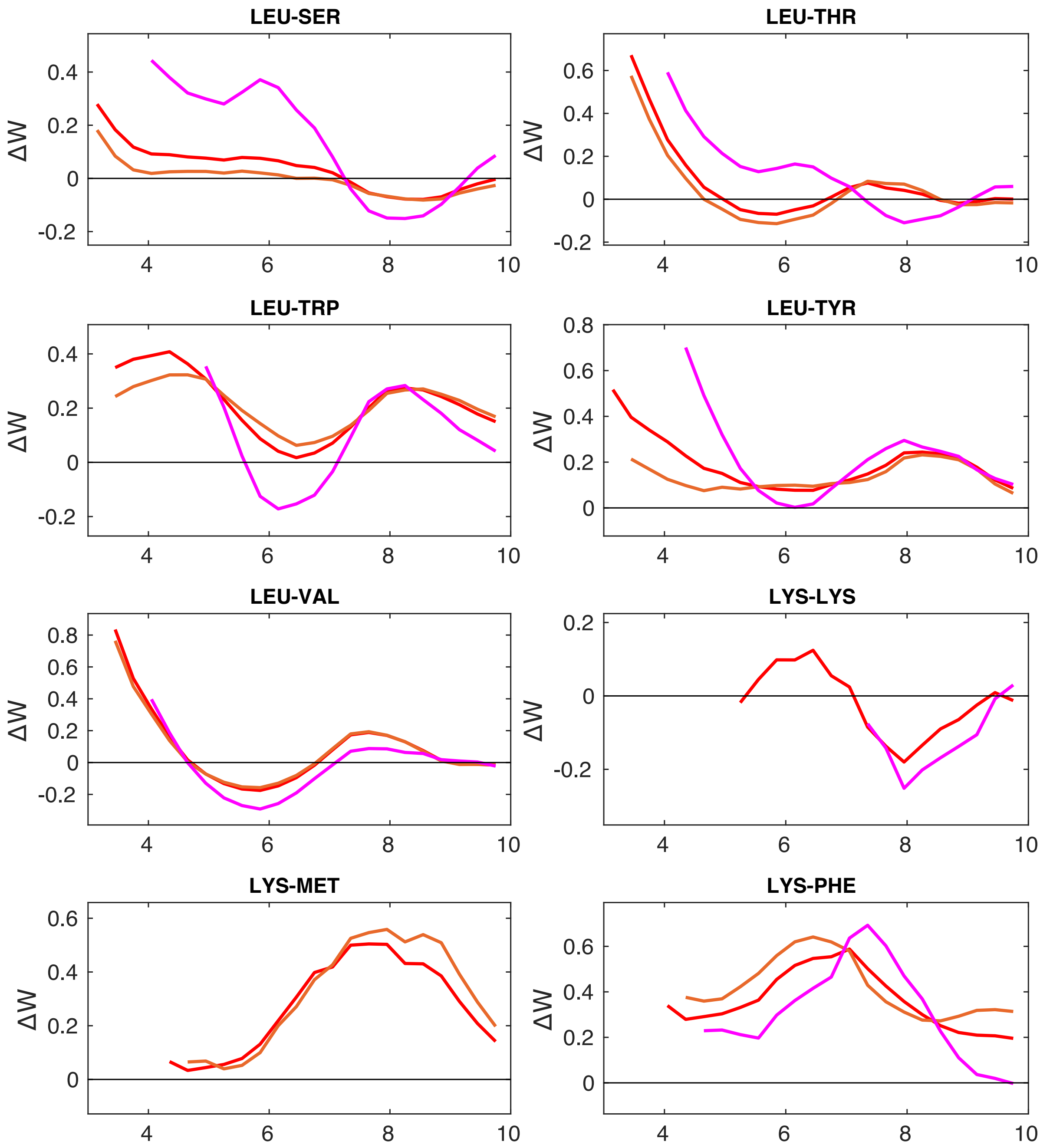
LEU-SER
LEU-THR
LEU-TRP
LEU-TYR
LEU-VAL
LYS-LYS
LYS-MET
LYS-PHE
ΔW

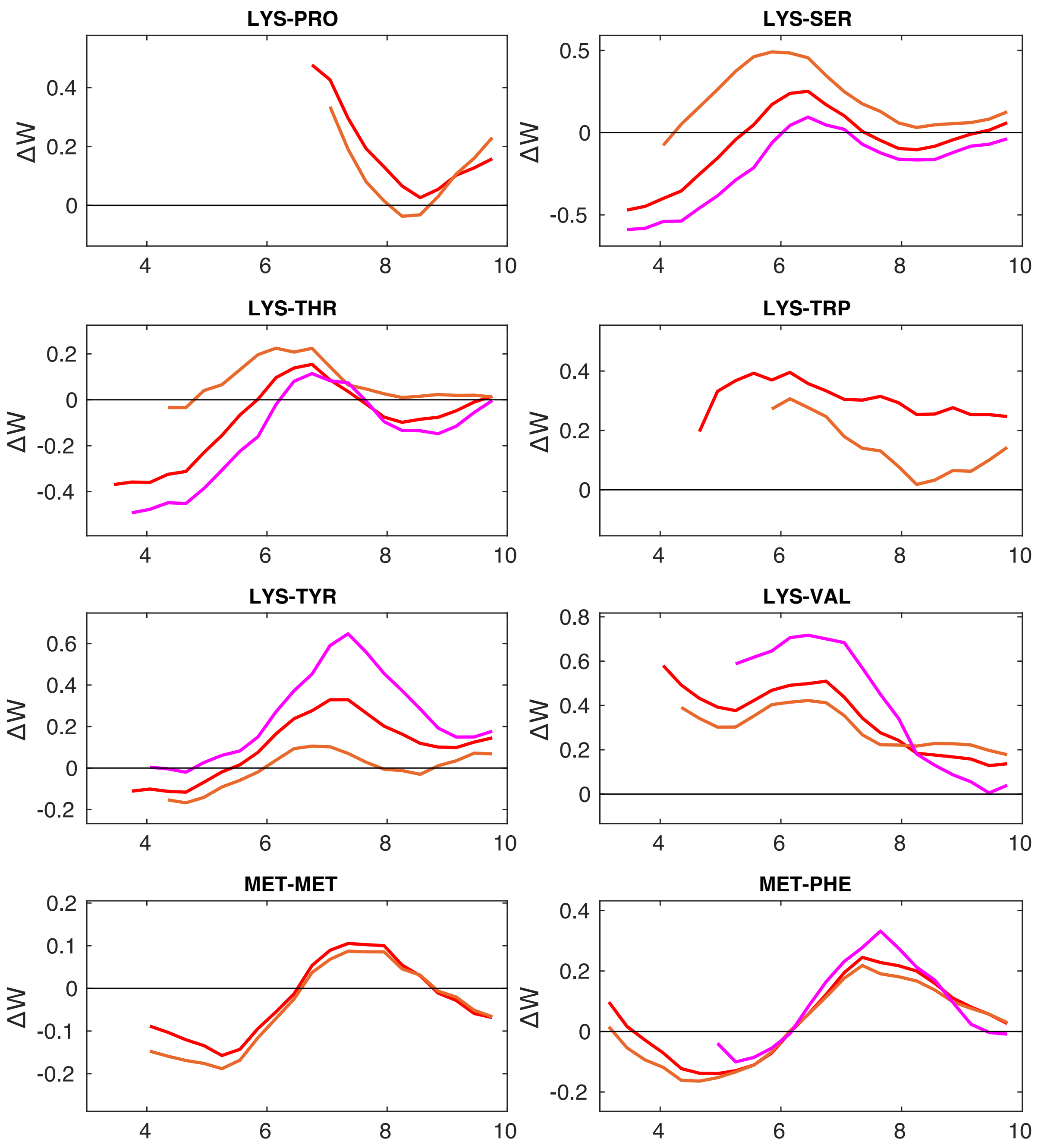

LYS-PRO
LYS-SER
LYS-THR
LYS-TRP
LYS-TYR
LYS-VAL
MET-MET
MET-PHE
ΔW

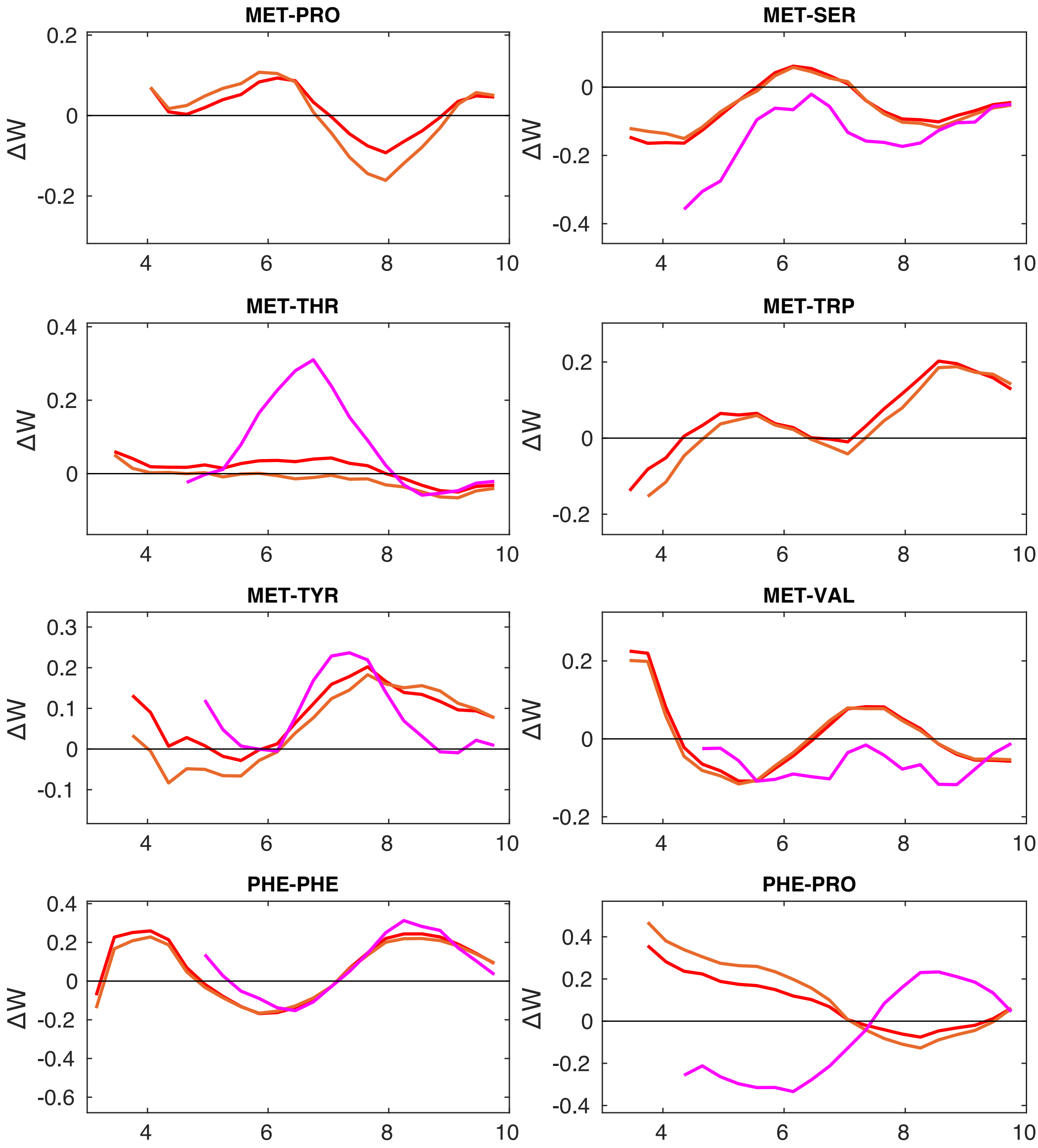
MET-PRO
MET-SER
MET-THR
MET-TRP
MET-TYR
MET-VAL
PHE-PHE
PHE-PRO
ΔW

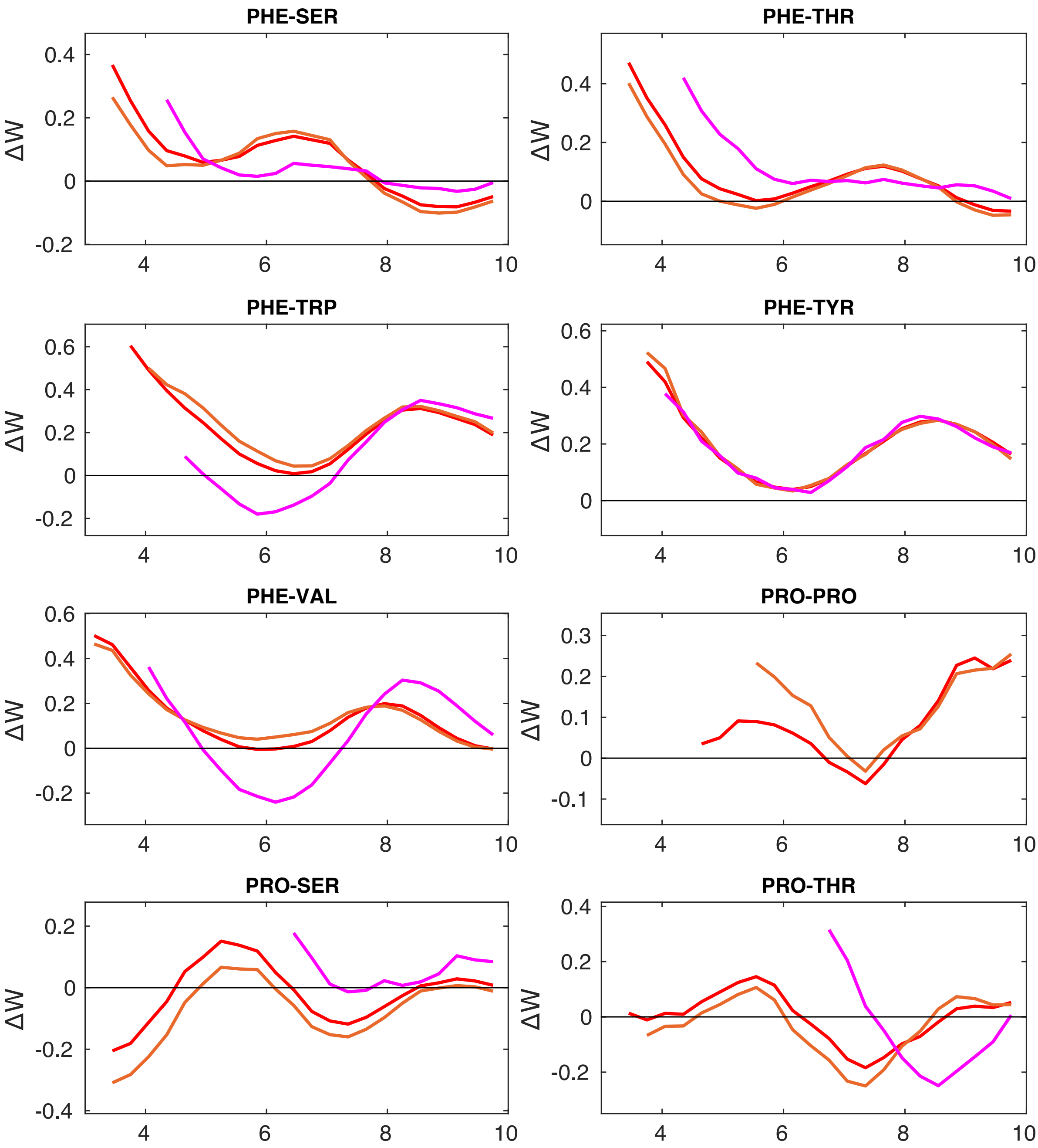
PHE-SER
PHE-THR
PHE-TRP
PHE-TYR
PHE-VAL
PRO-PRO
PRO-SER
PRO-THR
ΔW
4
6
8
10

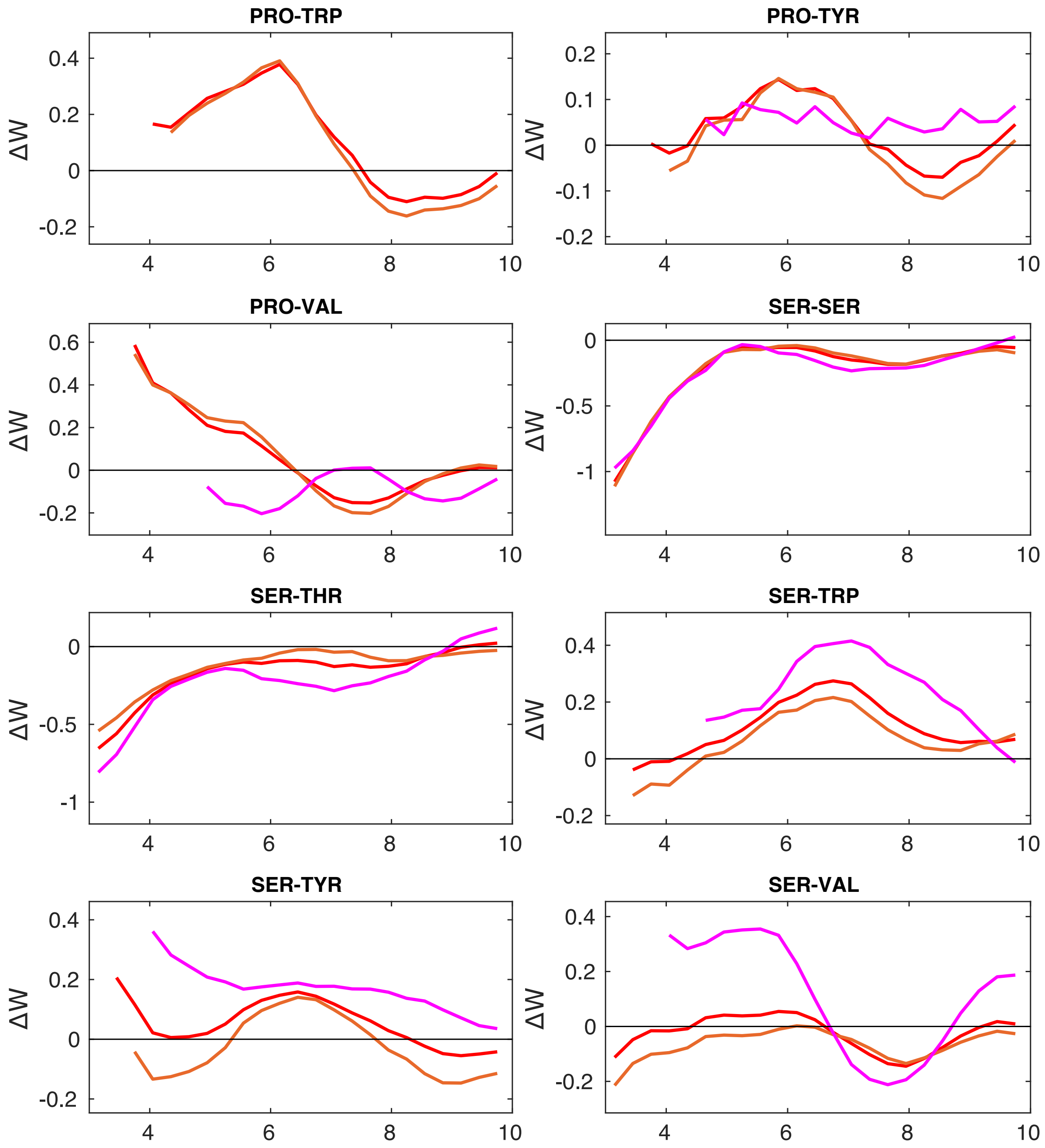

PRO-TRP
PRO-TYR
PRO-VAL
SER-SER
SER-THR
SER-TRP
SER-TYR
SER-VAL
ΔW

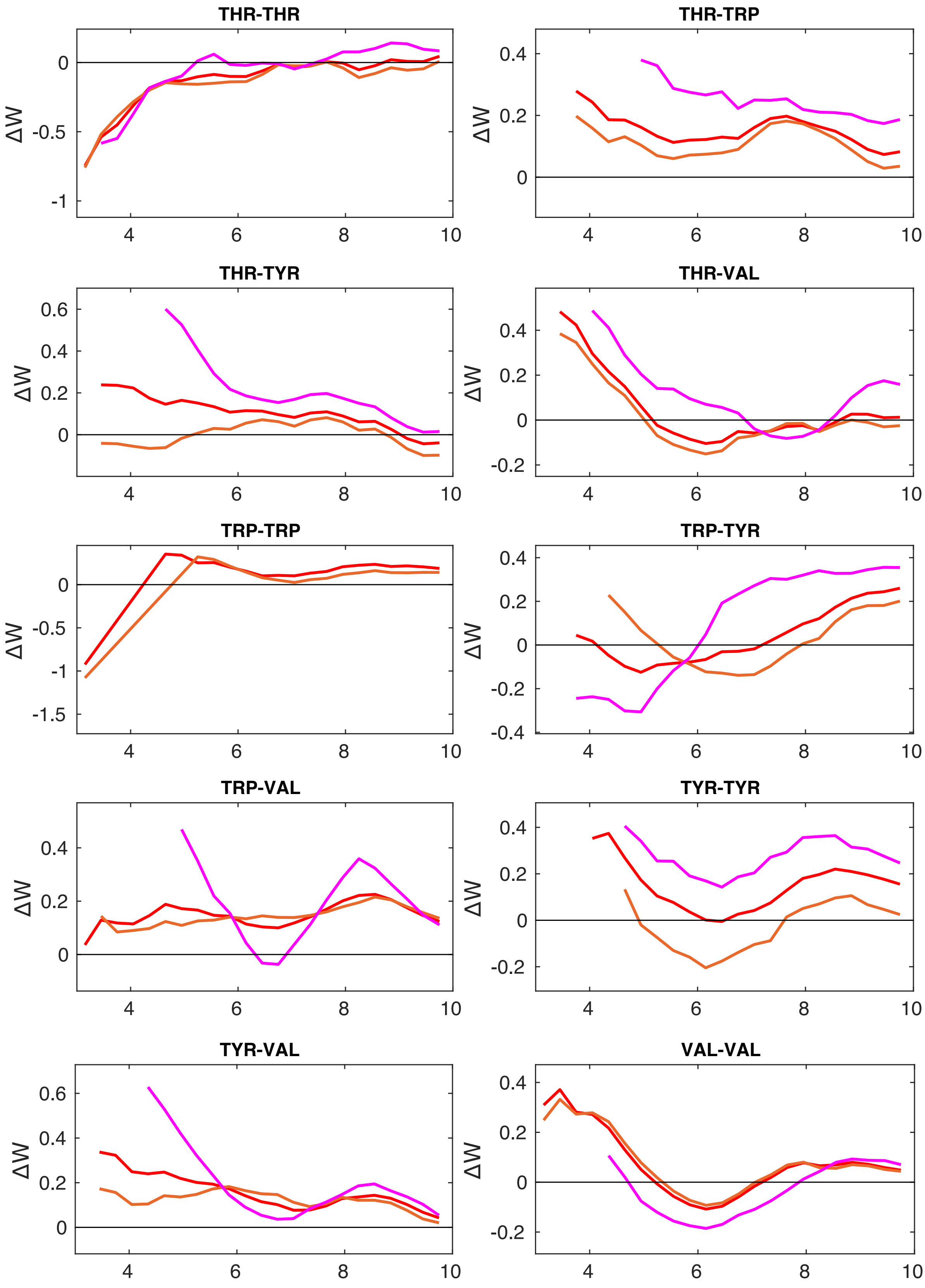
THR-THR
THR-TRP
THR-TYR
THR-VAL
TRP-TRP
TRP-TYR
TRP-VAL
TYR-TYR
TYR-VAL
VAL-VAL
ΔW
4
6
8
10

Figure S6. Statistical "sds" potentials between amino acid groups as a function of the distance (in Å), derived from the dataset $\mathcal{D}^{\mathrm{TM}}$ (red lines), or separately from α-helical (orange lines) and β-barrel (magenta lines) transmembrane regions. The numerical values are given in the Supplementary file Figure_S6.csv.

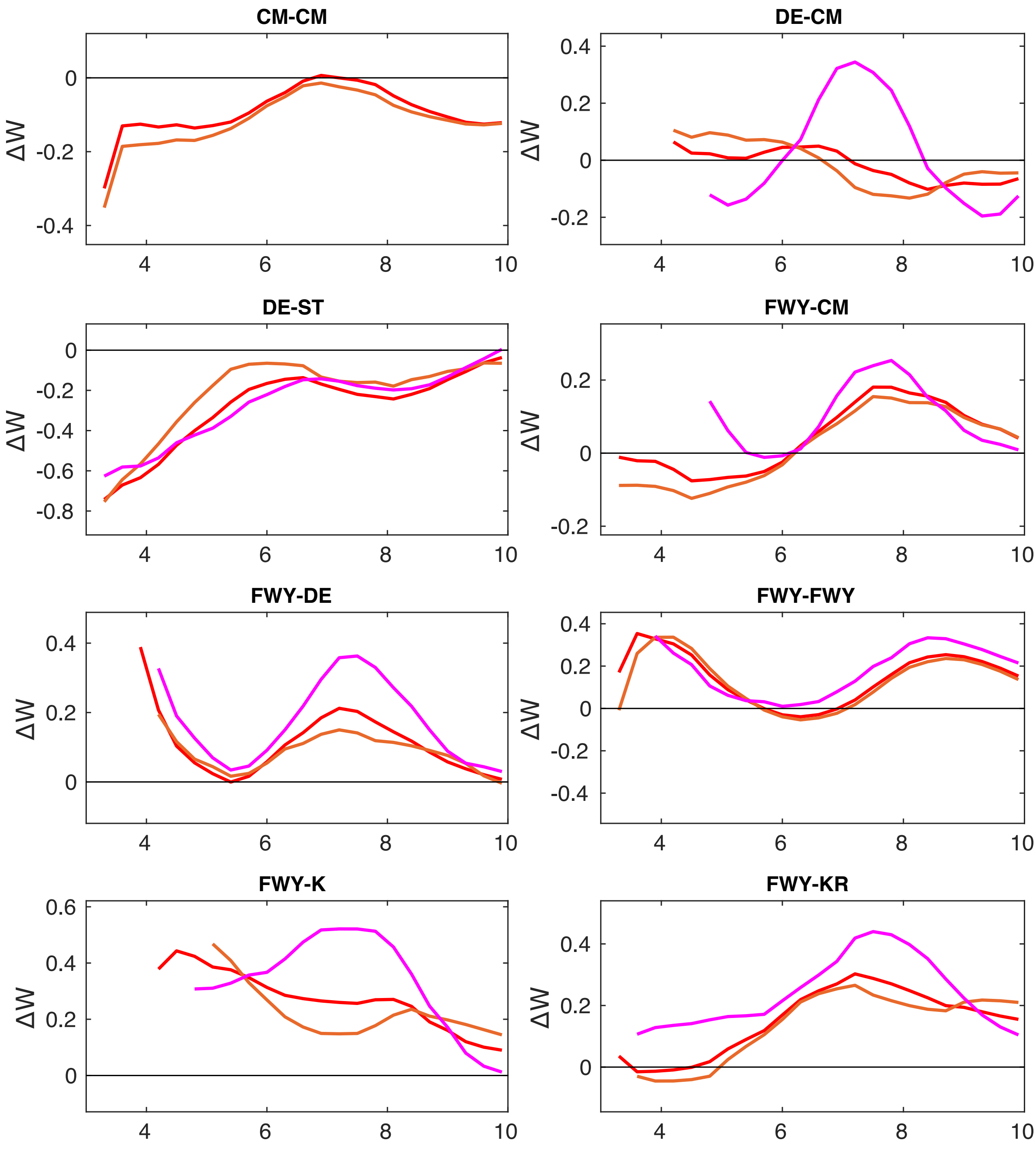

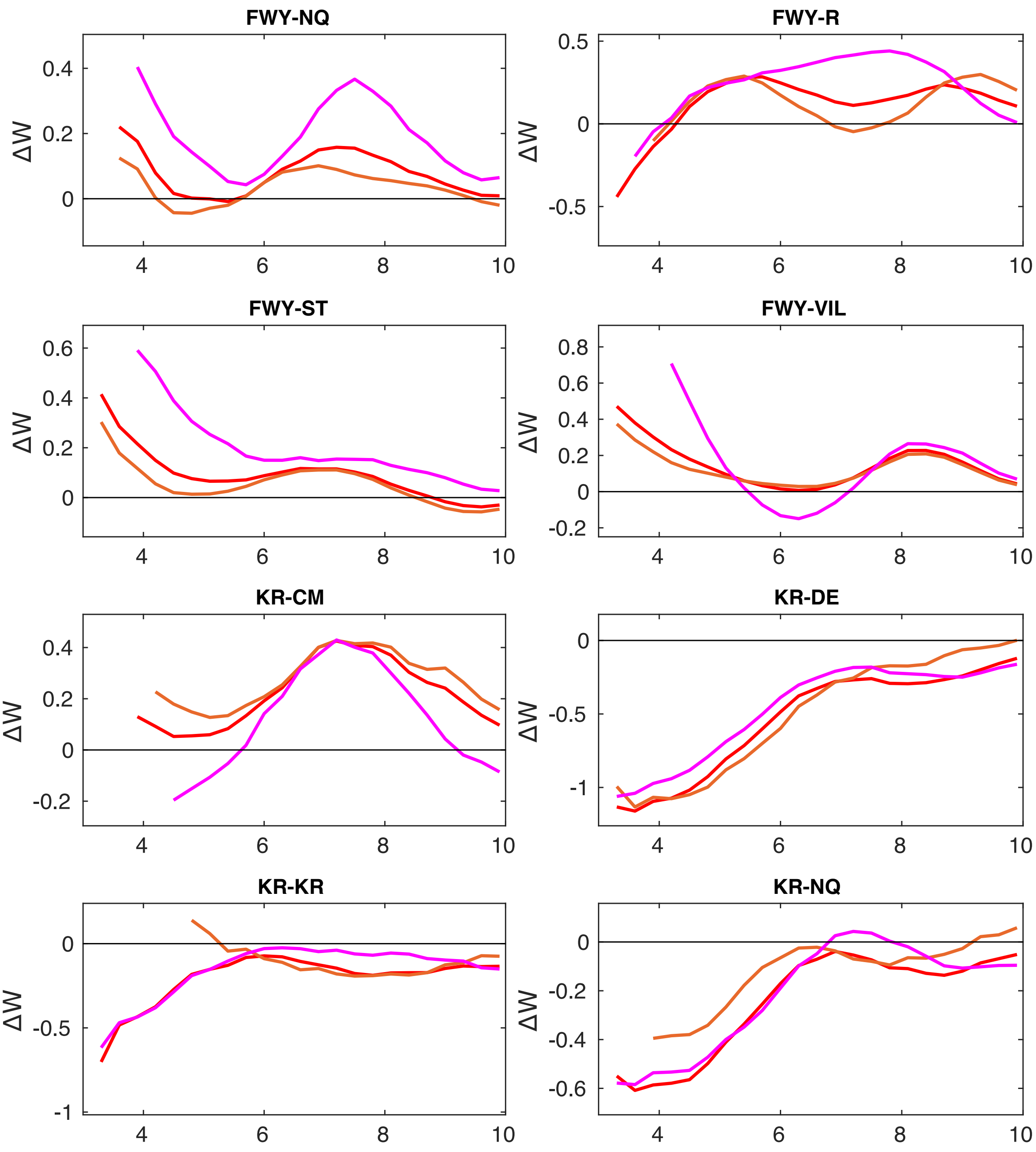
FWY-NQ
FWY-R
FWY-ST
FWY-VIL
KR-CM
KR-DE
KR-KR
KR-NQ
ΔW
0.4
0.2
0
0.5
-0.5
0.6
0.8
-0.2
-1
-0.4
-0.6
4
6
8
10

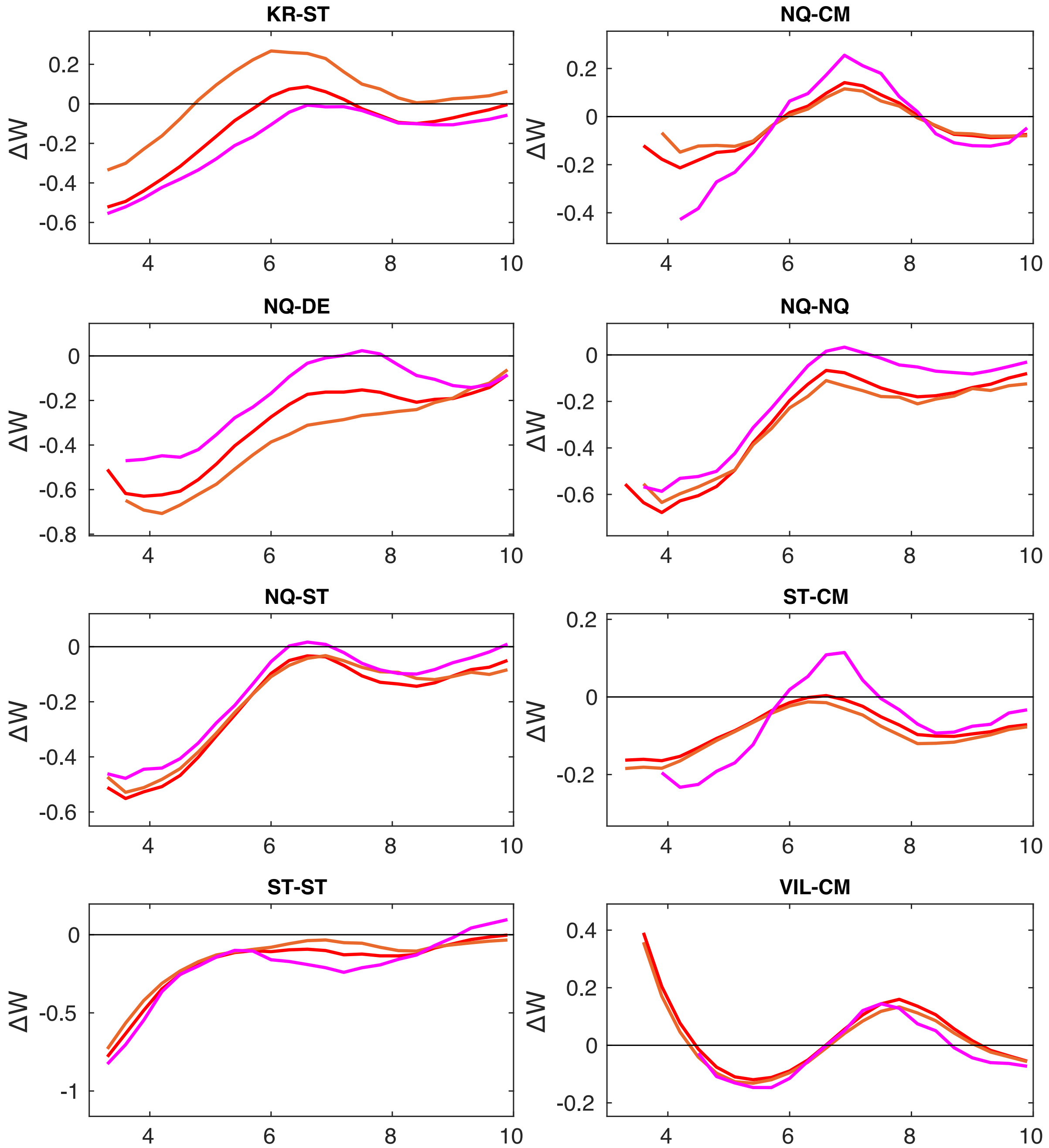
KR-ST
NQ-CM
NQ-DE
NQ-NQ
NQ-ST
ST-CM
ST-ST
VIL-CM
ΔW
4
6
8
10

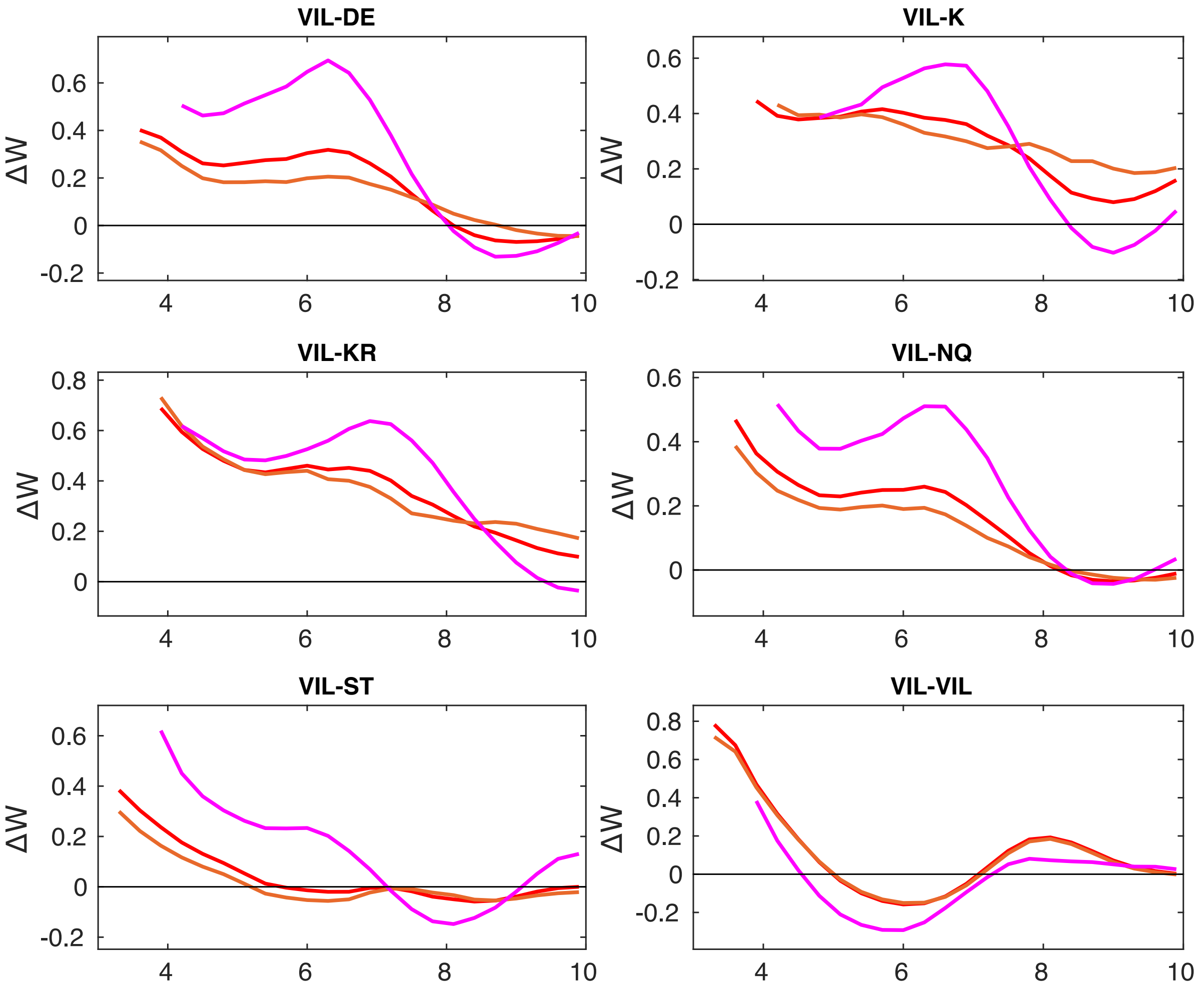
VIL-DE
VIL-K
VIL-KR
VIL-NQ
VIL-ST
VIL-VIL
ΔW
0.8
0.6
0.4
0.2
0
-0.2
4
6
8
10
